# Exo-Cleavable Linkers: Enhanced Stability and Therapeutic Efficacy in Antibody–Drug Conjugates

**DOI:** 10.1021/acs.jmedchem.4c01251

**Published:** 2024-10-16

**Authors:** Tomohiro Watanabe, Naoko Arashida, Tomohiro Fujii, Natsuki Shikida, Kenichiro Ito, Kazutaka Shimbo, Takuya Seki, Yusuke Iwai, Ryusuke Hirama, Noriko Hatada, Akira Nakayama, Tatsuya Okuzumi, Yutaka Matsuda

**Affiliations:** † Ajinomoto Co., Inc., 1-1, Suzuki-Cho, Kawasaki-Ku, Kawasaki-Shi, Kanagawa 210-8681, Japan; ‡ Ajinomoto Bio-Pharma Services, 11040 Roselle Street, San Diego, California 92121, United States

## Abstract

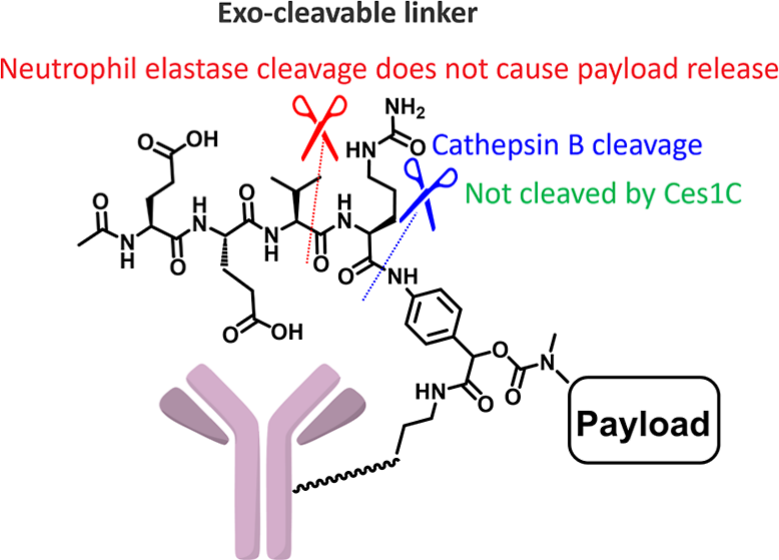

Antibody–drug conjugates (ADCs) combine cytotoxic payloads with monoclonal antibodies through chemical linkers. Finding linkers that both enhance circulatory stability and enable effective tumor payload release remains a challenge. The conventional valine-citrulline (Val-Cit) linker is associated with several inherent drawbacks, including hydrophobicity-induced aggregation, a limited drug–antibody ratio (DAR), and premature payload release. This study introduces an exolinker approach, repositioning the cleavable peptide linker at the exo position of the *p*-aminobenzylcarbamate moiety, as an advancement over conventional linear linkers. This design, which incorporates hydrophilic glutamic acid, addresses the limitations of the Val-Cit platform and improves the ADC in vivo profiles. In vitro and in vivo evaluations showed that exolinker ADCs reduced premature payload release, increased drug-to-antibody ratios, and avoided significant aggregation, even with hydrophobic payloads. Furthermore, the payload remained stably attached to the ADC even in the presence of enzymes like carboxylesterases and human neutrophil elastase, indicating the potential for a favorable safety profile.

## Introduction

Recent advances in targeted therapeutics have highlighted the need for precise drug delivery to maximize therapeutic indices while reducing systemic toxicity.^1^ Central to this shift is the antibody–drug conjugate (ADC), which is an intricate assembly of monoclonal antibodies (mAbs) and cytotoxic payloads.^2^ This hybrid system, fabricated using specialized chemical linkers, represents a significant advancement in disease intervention modalities, particularly in oncology and numerous other pathophysiological settings.^3^ Further, the innovative capability of this hybrid system is evidenced by the imprimatur of the U.S. Food and Drug Administration (FDA), which has endorsed a suite of 12 ADC entities for a range of hematological and solid malignancies. Furthermore, over 100 ADC constructs are currently undergoing rigorous clinical evaluation.^1−3^

The ability of mAbs to specifically target tumor cells is key to the ADC efficacy. mAbs enhance ADC potency, widen the therapeutic window, and improve treatment durability, offering advantages over traditional chemotherapy.^1−3^ However, it is important to emphasize that success in such endeavors is inseparable from the selected mAb or payload. Further, the linker plays a central role, influencing ADC properties such as structural homogeneity, pharmacokinetics (PK), and safety margins.^4^ Therefore, it is important to advance molecular acumen with respect to linker biology in realizing the potential of ADCs. Linkers have to be stable in the bloodstream and healthy tissues while efficiently delivering payloads to tumors. This complexity has led to extensive research in linker science, broadening its application beyond ADCs to other bioconjugates.^5^ Paradoxically, despite the apparent proliferation of linker choices,^5,6^ there is a significant monotony in clinical adoption. Several FDA-approved ADCs are based on the ubiquitous Val-Cit linker or its derived Val-Ala linkers.^7^

The mechanism of action of Val-Cit linkers depends on cathepsin B-mediated proteolysis following ADC endocytosis by target tumor cells, and these processes ensure immediate payload release.^5^ The stability of this well-established linker was further confirmed via robust stability assays in primate and human plasma models. However, it is associated with several limitations. The hydrophobic nature of the Val-Cit *p*-aminobenzylcarbamate (PAB) linker limits the amount of payload that can be used.^8^ In particular, common payload linkers, such as Mc-Val-Cit-PAB-MMAE, struggle with modest drug–antibody ratios (DAR = 3–4), and aspirations for higher ratios are thwarted by their hydrophobicity, which leads to aggregation. Additionally, enzymatic interference, which leads to premature linker cleavage and payload release, further weakens the Val-Cit chemistry. Notably, a landmark publication by Pfizer highlighted the vulnerability of the Val-Cit linker to carboxylesterase Ces1C, which results in premature payload detachment.^9^ Moreover, Zhao et al. revealed an additional issue associated with the aberrant cleavage of the Val-Cit bond involving human neutrophil elastase (NE), and this implies potential ADC-associated off-target toxicity, possibly leading to neutropenia.^10^ Several innovative strategies for overcoming the inherent drawbacks associated with the Val-Cit platform have been developed. Most of these strategies involve the use of hydrophilic polymer scaffolds, such as PEG,^11^ polysarcosine,^12^ cyclodextrins,^13^ peptides,^14^ and polyacetals,^15^ which are used to mitigate payload hydrophobicity. Additionally, the Tsuchikama group has developed linkers with hydrophilic moieties that resist noncathepsin B enzymes, featuring glutamic acid.^16,17^ These novel constructs, exemplified by the Ces1C-resistant Glu-Val-Cit (EVC)^16^ and NE-resistant Glu-Gly-Cit^17^ platforms herald a new era for linkers. Additionally, groundbreaking link format strategies involving tandem linkers,^18^ PEG-functionalizing PAB,^19^ and noncanonical amino acids have also been proposed.^6^ However, these strategies have some limitations. For example, the associated synthesis processes are complex and there exists a potential impact of immunogenicity on polymer molecules.^20^ Further, with respect to linear tripeptide linkers, the challenge of payload hydrophobicity still exists.

**Figure 1:**
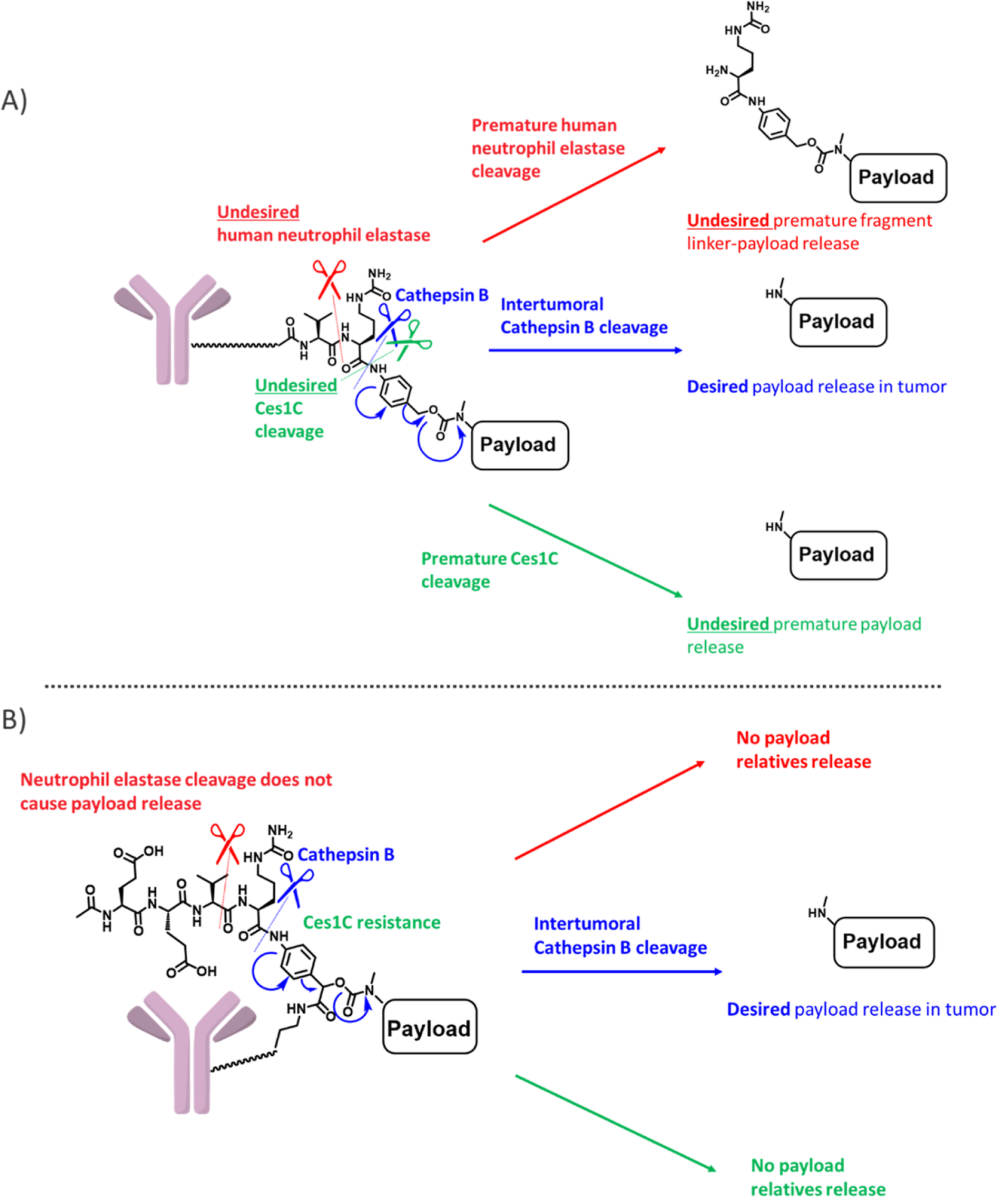
Comparison of Val-Cit PAB and exo-cleavable linkers. (A) Val-Cit PAB linker showing desired cathepsin B cleavage and undesired cleavage mediated by Ces1C and human NE. (B) Exo-cleavable linker showing desired cathepsin B cleavage and undesired cleavage resistance.

Therefore, in this study, we present a novel linker that aims to address the intrinsic drawbacks associated with the Val-Cit linker (Figure 1). We explored a novel design by introducing a cleavable peptide linker at the exo position (Figure 1B), rather than by using a conventional linear peptide linker. Although the introduction of a cleavable linker at the exo position has been reported in prior patents,^21,22^ these reports do not address the benefits of combining it with hydrophilic moieties (and the associated stability enhancement). Moreover, the biological evaluations in these prior reports are limited. Therefore, our approach repositions the peptide-cleavable linker Glu-Glu-Val-Cit (EEVC) or Glu-Val-Cit (EVC) at the exo position of the PAB moiety. This new exolinker strategy seems promising for effectively masking payload hydrophobicity by exploiting the hydrophilicity of tetrapeptides, including Val-Cit residues. Moreover, this Glu-containing linker not only offers resistance to Ces1C but also effectively prevents premature payload detachment mediated by human NE, a limitation observed with the benchmark linear EVC linker, which releases the payload under NE influence.

## Results and Discussion

### Evaluation of the Physical Properties of the Novel Exo-Cleavable Linkers

**Figure 2:**
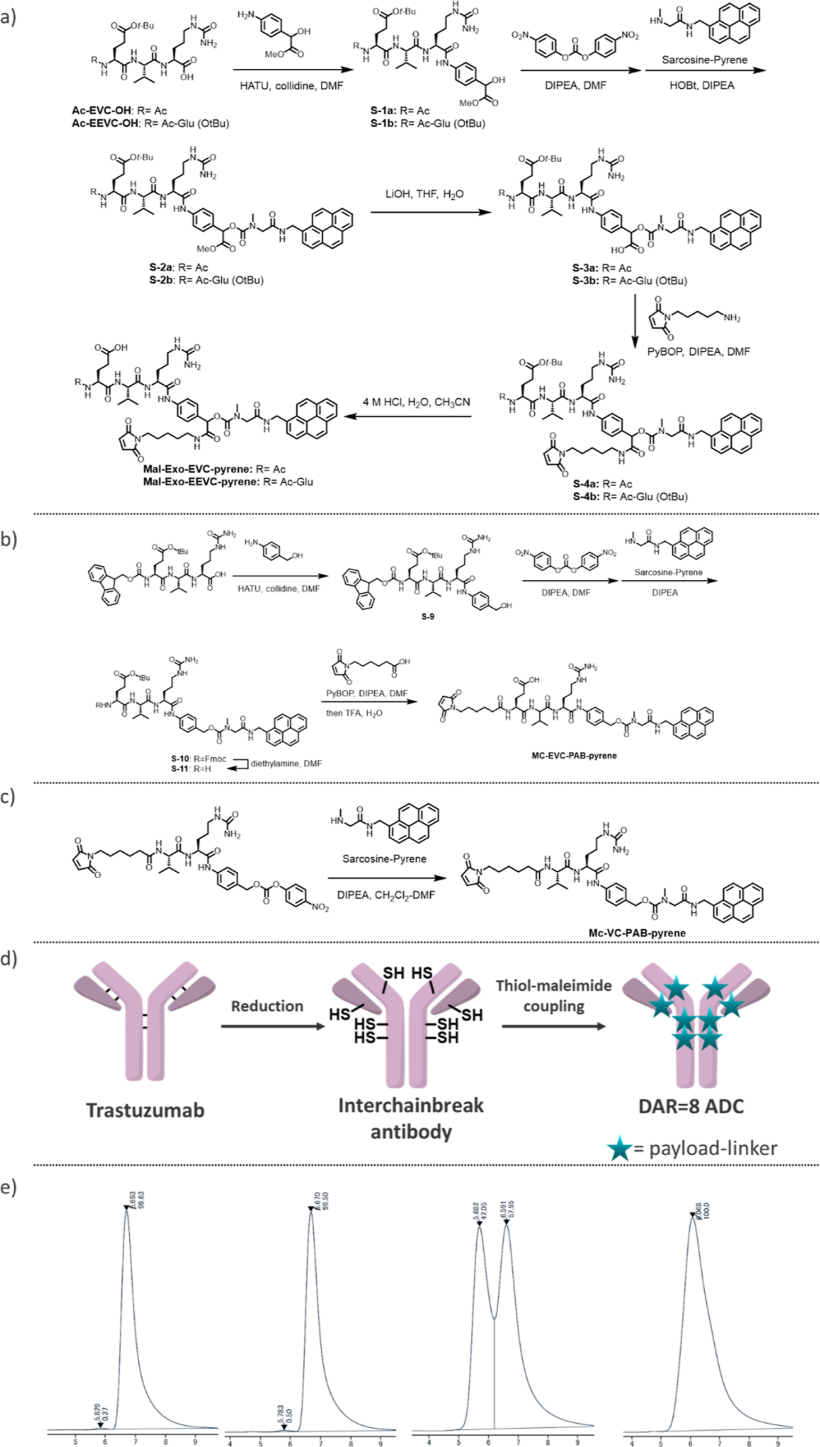
Comparison of the physical properties of Val-Cit PAB and exo-cleavable linkers. (A) Synthesis of Mal-Exo-EVC-pyrene and Mal-Exo-EEVC-pyrene. (B) Synthesis of Mc-linear-EVC-PAB-pyrene. (C) Synthesis of Mc-VC-PAB-pyrene. (D) Illustration of the synthesis of ADC with a DAR of 8. (E) SEC analysis of ADCs, Exo-EVC-pyrene ADC (left), Exo-EEVC-pyrene ADC (second left), linear-EVC-pyrene ADC (second right), and linear-VC-pyrene ADC (right).

To investigate the potential of the novel exolinker, we performed a comparative study using a high-DAR (8) ADC alongside the traditional Val-Cit linker (Figure 2 and Table 1). Considering the inherent risk of aggregation associated with the exolinker owing to its hydrophobic and planar structure, pyrene was chosen as the payload based on its facile synthesis process and amenability to straightforward fluorescence assays. Therefore, by leveraging a known sarcosine molecule,^16^ we successfully developed a pyrene-based payload and subsequently coupled it to the exolinker, Exo-EVC-PAB–OH. This was followed by the introduction of a maleimide molecule, which led to the synthesis of Mal-Exo-EVC-pyrene. The detailed synthetic routes are described in the Experimental Section. We also synthesized Mal-Exo-EEVC-pyrene. For validation, control molecules, namely, Mc-VC-PAB-pyrene and linear Mc-EVC-PAB-pyrene, were also prepared. ClogP and AlogP evaluations using a previously reported procedure^23^ highlighted the distinct hydrophilicity of the exolinker pyrenes. Using these payloads, ADCs with a DAR of 8 were synthesized via interchain-break conjugation. The work of Lyon et al. highlighted the existence of a correlation between ADC retention time during hydrophobic interaction chromatography (HIC) and systemic clearance, suggesting that accelerated retention times during HIC are indicative of favorable hydrophilic properties.^24^ Notably, the significantly faster retention dynamics of trastuzumab-*exo*-EVC-pyrene (ADC (1)) and trastuzumab-*exo*-EEVC-pyrene (ADC (2)) relative to those of linear trastuzumab-VC-pyrene (ADC (3)) and linear trastuzumab-EVC-pyrene (ADC (4)) highlighted their hydrophilic attributes. Further, analytical findings based on size-exclusion chromatography (SEC)^25^ revealed a strong disparity in aggregation, with ADC (3) and ADC (4) exhibiting pronounced aggregation, while aggregation profiles of ADC (1) and ADC (2) showed no alterations relative to that of native trastuzumab. In a Ces1C-containing mouse plasma environment, the exolinker exhibited commendable stability. The variation of the concentration of free pyrene-related compounds remained below 5% even after 4 days of incubation. This robustness was complemented by the retention of the cathepsin cleavage. Notably, even though *exo*-ADC (1) and linear-ADC (4) have nearly identical chemical formulas (EVC), subtle repositioning of the cleavable linker yielded significantly different results. This is consistent with our preliminary hypothesis that the masking effect inherent in the cleavable peptide site, in synergy with the structural proximity between the antibody and the payload, collectively enhances ADC properties.

**Table 1:** Summary of the Comparison of the Physical and Mouse Plasma Stabilities of Val-Cit PAB and Exo-Cleavable Linkers

antibody conjugates	linker configuration	linker-payload	Clog P of linker-payload	Alog P of linker-payload	HIC retention time of ADC (min)	DAR in HIC	aggregation in SEC (%)	released payload in mouse plasma (%)
trastuzumab					5.8		0.5	
ADC (1)	Exo-EVC	Mal-Exo-EVC-pyrene	2.21	2.06	9.7	8.0	0.4	3.5
ADC (2)	Exo-EEVC	Mal-Exo-EEVC-pyrene	1.06	1.31	9.1	7.4	0.5	2
ADC (3)	linear-VC	Mc-VC-PAB-pyrene	4.31	3.87	15.0	7.9	100	36
ADC (4)	linear-EVC	Mc-EVC-PAB-pyrene	3.17	3.12	14.9	7.8	42	7

### Application to Cytotoxic ADCs

**Figure 3:**
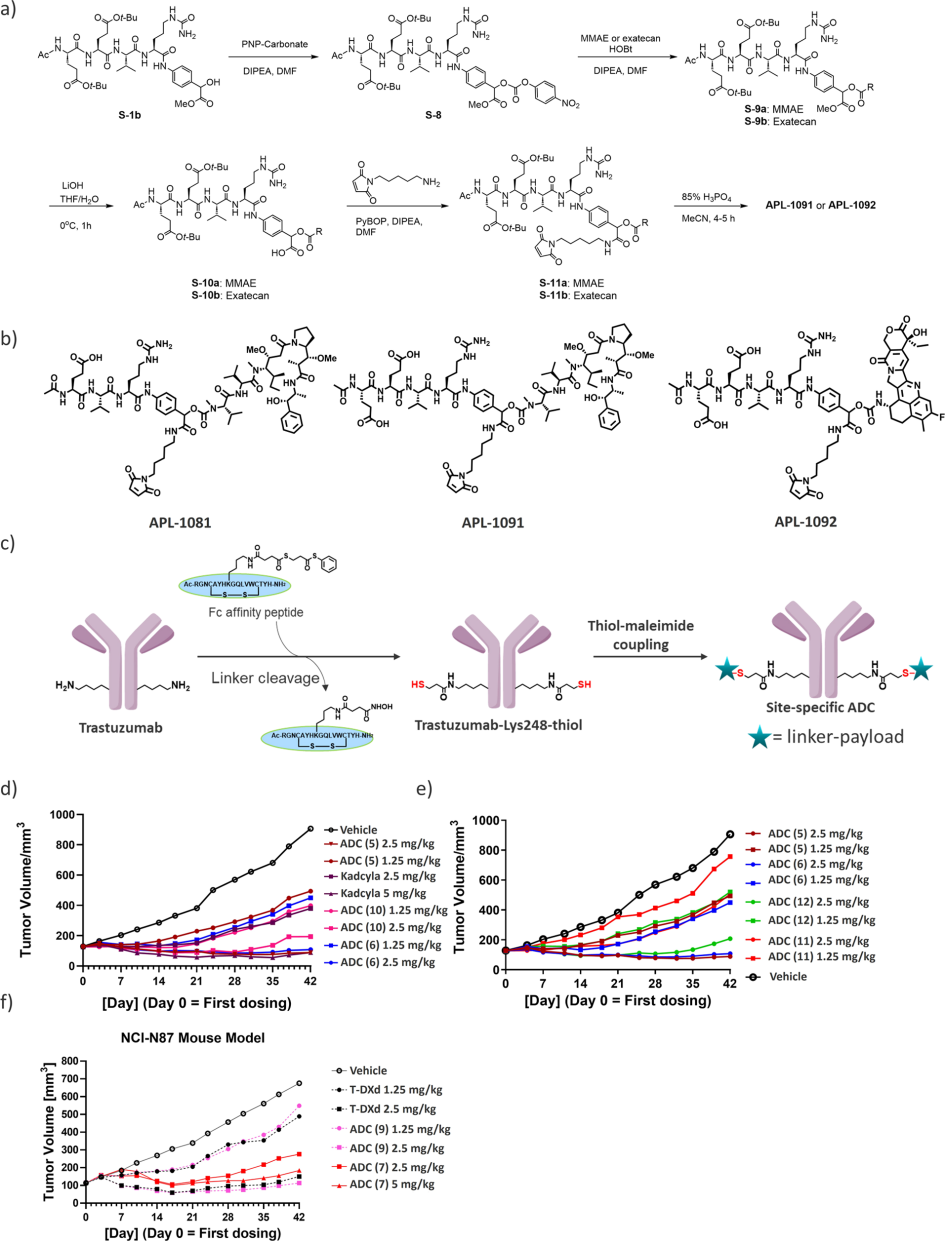
Application to cytotoxic payloads. (A) Synthesis of APL-1091 (Mal-Exo-EEVC-MMAE) and APL-1092 (Mal-Exo-EEVC-Exatecan). (B) Chemical structures of APL-1081 (Mal-Exo-EVC-MMAE), APL-1091 (Mal-Exo-EEVC-MMAE), and APL-1092 (Mal-Exo-EEVC-Exatecan). (C) Schematic overview of AJICAP site-specific technology to produce DAR = 2 ADCs. (D) NCI–N87 in vivo xenograft studies of MMAE-based ADCs compared with Kadcyla, (E) NCI–N87 in vivo xenograft studies of MMAE-based ADCs (AJICAP DAR = 2) to compare the cleavable linker, and (F) NCI–N87 in vivo xenograft studies of Exatecan-based ADCs.

To further validate the potential of the novel exolinker, we conjugated it with highly cytotoxic payloads, MMAE and exatecan, which are widely recognized and commonly used in commercial ADCs (Figure 3 and Table 2). Thus, its use led to the synthesis of Mal-Exo-EVC-MMAE (APL-1081), Mal-Exo-EEVC-MMAE (APL-1091), and Mal-Exo-EEVC-Exatecan (APL-1092) (Figure 3A,B).

**Table 2:** Summary of the ADCs

antibody conjugates	conjugation method	linker configuration	linker-payload (payload)	ADC retention time in HIC (min)	DAR in HIC	aggregation in SEC (%)
trastuzumab				5.8		0.4
ADC (5)	AJICAP	Exo-EVC	APL-1081 (MMAE)	9.1	2.0	1.6
ADC (6)	AJICAP	Exo-EEVC	APL-1091 (MMAE)	8.7	2.0	1.4
ADC (7)	AJICAP	Exo-EEVC	APL-1092 (Exatecan)	8.0	2.0	1.0
ADC (8)	interchainbreak	Exo-EEVC	APL-1091 (MMAE)	11.3	7.8	0.5
ADC (9)	interchainbreak	Exo-EEVC	APL-1092 (Exatecan)	6.8	7.9	1.0
ADC (10)	interchainbreak	linear-VC	Mc-VC-PAB-MMAE	11.7	4.1	1.2
ADC (11)	AJICAP	linear-VC	Mc-VC-PAB-MMAE	10.8	1.9	1.8
ADC (12)	AJICAP	linear-EVC	Mc-EVC-PAB-MMAE	10.8	1.9	1.7
T-Dxd	interchainbreak	linear-GGFG	Deruxtecan	9.1	7.8	0.1

As recently demonstrated, site-specific conjugation tends to provide ADCs with a broader therapeutic window than their random counterparts.^26,27^ Thus, we used the second-generation AJICAP method involving an Fc affinity molecule to selectively convert native antibodies into site-specific ADCs (Figure 3C).^27^ Thus, we generated trastuzumab by introducing a thiol group at the Lys248 site. Subsequent conjugation of the Lys248 thiol with APL-1081, APL-1091, and APL-1092 resulted in a targeted DAR of approximately 2 (ADC (5): APL-1081 DAR = 2; ADC (6): APL-1091 DAR = 2; and ADC (7): APL-1092 DAR = 2). Notably, these three payload-linkers solubilized in the conjugation buffer without the need for the addition of typical cosolvents. This highlighted the universal applicability of the exolinker, even for antibodies that are potentially unstable in organic solvents. To further demonstrate the beneficial properties of the exolinker, ADCs with a DAR of 8 were synthesized from both APL-1091 and APL-1092 (ADC (8): APL-1091 DAR = 8; ADC (9): APL-1092 DAR = 8). All of these constructs exhibited acceptable HIC retention times and aggregation levels. Even though the known tendency of MMAE to aggregate was evident at a DAR of 8,^29^ APL-1091 did not exhibit this tendency, confirming its hydrophilic nature.

### In Vivo Xenograft Studies of the Novel Exolinker ADCs

In this study, we also evaluated the resulting ADCs using NCI–N87 xenograft mice (Figure 3D–F). Thus, we were able to compare our ADCs with key industry-standard ADCs. APL-1081 and APL-1091 were comparable to a trastuzumab ADC (ADC 10) with a DAR of 4 owing to stochastic interchain cysteine conjugation, which mirrors the molecular formats of commercial ADCs, such as Adcetris and Polivy.^7,30^ Additionally, these ADCs (5 and 6) exhibited higher in vivo efficacy than the clinical ADC Kadcyla (Figure 3D). Further, two site-specific ADCs (ADC 11 and ADC 12) were designed with traditional Mc-VC-PAB-MMAE and linear Mc-EVC-PAB-MMAE, which is known as the optimized version of the VC linker, as the payload linkers using the AJICAP second-generation method in a site-specific manner (Figure 3E and Table 3).^28^ This allowed for a clearer comparison of the linkers. Notably, APL-1081-based ADC 5 showed reasonable antitumor efficacy even at doses as low as 2.5 mg/kg, outperforming its counterparts, such as Mc-VC-PAB-MMAE-based ADC 11. This efficacy was comparable with that of the benchmark ADC 12 consisting of linear Mc-EVC-PAB-MMAE. These results indicate that the hydrophilic amino acid (Glu) contributes to the enhancement of ADC efficacy in both linear and exotype linkers. However, as previously mentioned, HIC analysis revealed that ADC 5, an Exo-EVC ADC, is more hydrophilic than the linear EVC-ADC (ADC 12), suggesting that ADC 5 is a more promising candidate. Furthermore, this highly hydrophilic APL-1091 was successfully applied to a DAR = 8 ADC (ADC8). Although it is generally challenging to apply MMAE to DAR8 due to its high hydrophobicity, we were able to obtain an ADC without any increase in aggregation. Further evaluation of this MMAE DAR8 instrument is currently underway.

**Table 3:** Study Arms in the NCI-N87 First Run

group	N	agent	mg/kg
1	10	Vehicle	
2	10	trastuzumab-emtansine (Kadcyla)	5
3	10	trastuzumab-emtansine (Kadcyla)	2.5
4	10	ADC 5 (AJICAP, APL-1081)	2.5
5	10	ADC 5 (AJICAP, APL-1081)	1.25
6	10	ADC 6 (AJICAP, APL-1091)	2.5
7	10	ADC 6 (AJICAP, APL-1091)	1.25
8	10	ADC 10 (interchain-break, Mc-VC-PAB-MMAE)	2.5
9	10	ADC 10 (interchain-break, Mc-VC-PAB-MMAE)	1.25
10	10	ADC 11 (AJICAP, Mc-VC-PAB-MMAE)	2.5
11	10	ADC 11 (AJICAP, Mc-VC-PAB-MMAE)	1.25
12	10	ADC 12 (AJICAP, Mc-EVC-PAB-MMAE)	2.5
13	10	ADC 12 (AJICAP, Mc-EVC-PAB-MMAE)	1.25

In a parallel study, the APL-1092-based ADC was evaluated against trastuzumab-deruxtecan (T-DXd, Enhertu), a market-leading ADC with a DAR of 8 (Figure 3E and Table 4). The dose was adjusted to match the payload. Thus, we observed that the ADC conjugated to APL-1092, ADC 7, showed the most pronounced antitumor activity and demonstrated substantial therapeutic efficacy at a dose of 2.5 mg/kg. Further, it showed a dose-dependent tumor-inhibitory effect on NCI-N87 cells, and when normalized to the incorporated payload amount, it showed superior tumor inhibitory effects compared to those of trastuzumab-deruxtecan. Furthermore, ADC 9, with a DAR of 8, analogous to trastuzumab-deruxtecan, showed significant tumor growth inhibitory effects at the tested doses, 1.25 and 2.5 mg/kg (Experimental Section).

**Table 4:** Study Arms in the NCI-N87 Second Run

group	N	agent	mg/kg
1	10	vehicle	
2	10	trastuzumab-deruxtecan (enhertu)	2.5
3	10	trastuzumab-deruxtecan (enhertu)	1.25
4	10	ADC 7 (AJICAP, APL-1092)	10
5	10	ADC 7 (AJICAP, APL-1092)	5.0
6	10	ADC 7 (AJICAP, APL-1092)	2.5
7	10	ADC 9 (interchain-break, APL-1092)	2.5
8	10	ADC 9 (interchain-break, APL-1092)	1.25

These in vivo efficacy studies indicated that the exolinker produces efficacious ADCs owing to its increased stability in mouse plasma as well as the benckmark linear EVC linker. This efficacy was further enhanced when the exolinker was combined with second-generation AJICAP technologies.

### Rat Pharmacokinetics Studies of the Exolinker ADCs

**Figure 4:**
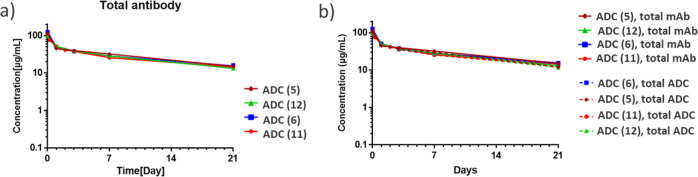
Pharmacokinetic study of exolinker-based ADCs in rats. (A) Analysis of total antibody using ELISA. (B) Combined trend of total antibody and total ADC. Trend in average DAR determined via LC–MS assay followed by multiplying the average DAR by the total antibody concentration to calculate total ADC.

In the field of rat PK studies, LC–MS was necessary. Historically, the gold standard methodology for PK studies has been the use of antipayload antibodies via ELISA. However, this exolinker introduces a unique design nuance based on the ability of the linker to mask the payload. This feature is associated with concerns regarding steric hindrance, which can prevent efficient recognition by antipayload antibodies. Therefore, to examine this concern, we performed an LC–MS-based assay for the total ADC measurement. In this study, we first focused on site-specific MMAE-based ADCs (ADC 5, 6, 11, and 12, Figure 4). Specifically, after dosing, blood samples were carefully collected from the test animals using biotinylated antihuman IgG-Fc fragment-coated beads. The samples were then split for the measurement of total antibody and ADC. For the total antibody measurement, we utilized the ELISA method with an anti-Fc antibody. For the total ADC measurement, we employed LC–Q-TOF MS analysis to determine the average DAR and the apparent ADC concentration, which was calculated by multiplying the average DAR by the total antibody concentration provided by ELISA. The results revealed nuanced interpretations of the DAR transitions derived from the Q-TOF MS deconvolution spectra. The high stability characteristics were comparable among all AJICAP-ADCs. Notably, having previously delineated the trajectory of total ADC for ADC (11) via conventional ELISA, we sought to bridge the assay methodologies. Thus, we subjected ADC (11) to an LC–MS assay (Supporting Information, Figure S42). Although minor variations were observed, with slightly elevated DAR values obtained for ADC (11) via a ligand binding assay, the divergence was minimal, and the peak DAR difference was only 0.2. These comparative analyses indicated that ADCs (5), (6), and (12) containing the hydrophilic amino acid (Glu) exhibited better payload linker retention than ADC (11) with the traditional Val-Cit linker.

Separately, APL-1092-based ADC (7) was also analyzed by this hybrid LC–MS method, showing that over 80% of its payload remained integrally bound at day 21 (Supporting Information, Figure S43). These results indicated that the exolinker was comparable with the known and optimized EVC linker, and this stability feature is commonly observed in multiple payload constructs.

### In Vitro Human Neutrophil Elastase Assay of Exolinker ADCs

**Figure 5:**
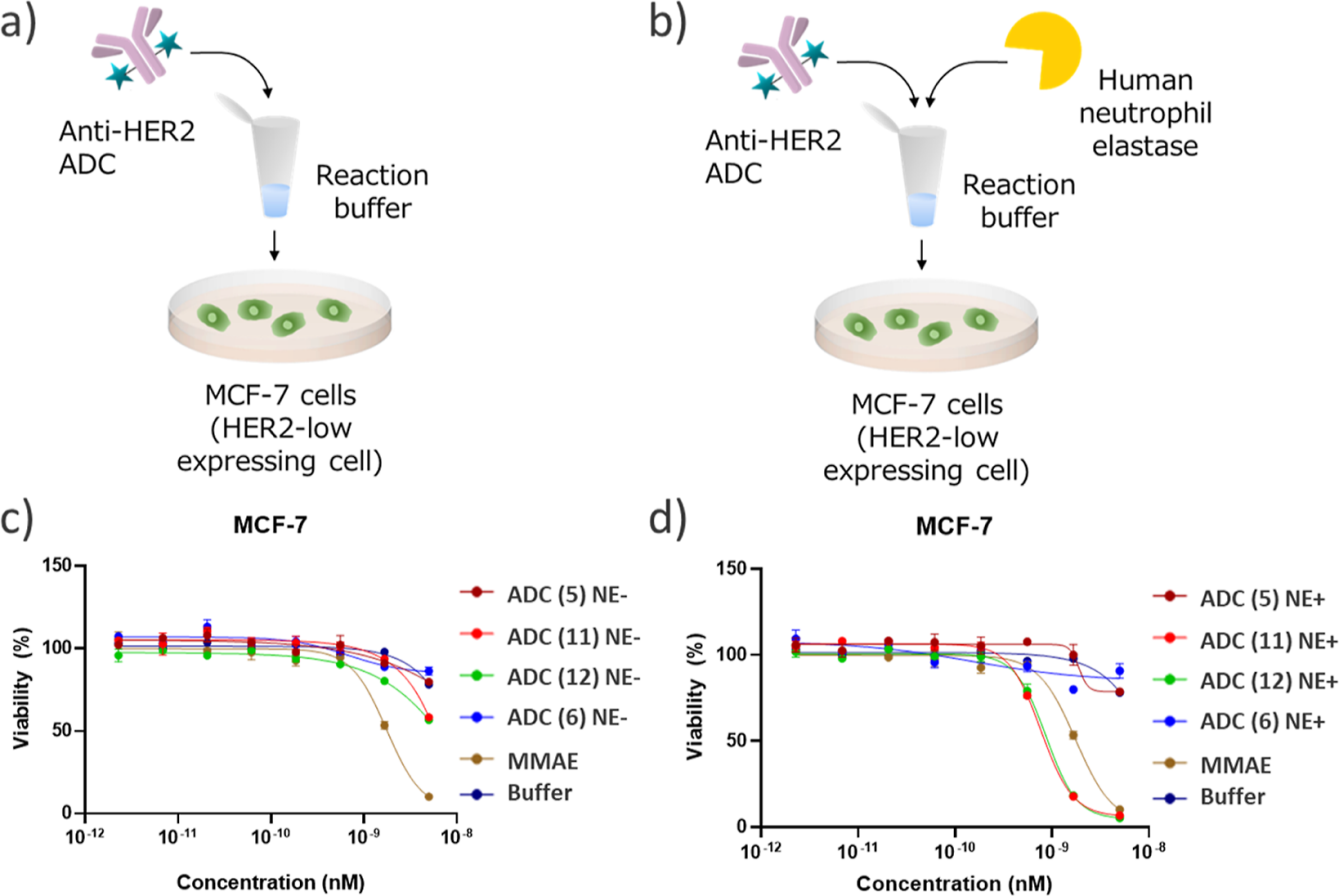
Evaluation of the off-target toxicity of ADCs using an in vitro cytotoxicity assay. (A,B) Schematic representation of the assay under (A) NE-depleted and (B) NE-pretreated conditions. anti-HER2 ADCs treated or not treated with NE in the NE reaction buffer and incubated with MCF-7 cells for 6 days. (C,D) Viability of cells incubated with 2.3 pM–5.0 nM of (C) not treated and (D) NE-treated ADCs and MMAE (5). Individual values and fitted curves are shown based on the results of triplicate experiments.

In the developing discourse regarding the Val-Cit linker, its undesired cleavage by human NE has emerged as a pivotal issue.^10,17^ Experimental evidence suggests that NE cleaves the peptide bond nestled between valine and citrulline in the Val-Cit linker. This enzymatic interaction triggers the conversion of the Val-Cit PAB payload to a Cit PAB payload,^17,31^ and this supposedly, results in off-target toxicity. In this study, we observed that even in the presence of NE-mediated cleavage, the exolinker remained attached to the payload linker. This inherent robustness suggested a significant decrease in payload detachment, thereby providing protection against potential adverse effects. Further, to test this hypothesis, an in vitro cytotoxicity assay was designed to detect off-target toxicity caused by NE (Figure 5A,B). In this setup, ADCs with the Val-Cit linker (ADC 11), linear EVC linker (ADC 12), exo-EVC linker (ADC 5), and exo-EEVC linker (ADC 6) were incubated with NE, while the control ADCs, which were untreated, were incubated with model cells. In HER2-positive model cells (SKBR-3),^32^ all the ADCs with the Val-Cit linker, EVC linker, or exolinkers showed cytotoxicity against SKBR-3 cells regardless of NE treatment (Supporting Information Figure S70). This indicated that our cell-based cytotoxicity assay was not affected by NE treatment. Next, we performed an assay using the HER2-negative MCF-7 cell line. MMAE without linkers was used as a benchmark. In the NE-free environment, the potencies of all of the entities, except that of MMAE, remained unchanged (Figure 5C). In contrast, in the NE-treated milieu, ADC 11 consisting of the Val-Cit linker displayed pronounced cytotoxic attributes, exhibiting an IC_50_ that was an order of magnitude greater than that of MMAE (Figure 5D). Moreover, ADC 12 consisting of a linear Glu-Val-Cit linker also showed cytotoxicity. It has been postulated that ADC 11 or 12, endowed with Mc-VC-PAB-MMAE or Mc-EVC-PAB-MMAE, respectively, releases Cit-PAB-MMAE upon exposure to NE. The resulting Cit-PAB–MMAE, potentially driven by the hydrophobic propensity of its Cit-PAB segment, may then exhibit enhanced intracellular penetration compared to MMAE. We further confirmed the differences in cleavage through Q-TOF/MS analysis, which clearly distinguishes between the cleaved and uncleaved forms. We analyzed both the linear and exo EVC-MMAE ADCs using Q-TOF/MS after NE cleavage (Figure S71, Supporting Information). The linear EVC-MMAE ADC (ADC 12) exhibited a significant MS shift, indicating the release of Cit-PAB-MMAE, while the exo-EVC-MMAE ADC (ADC 5) showed only a slight decrease in the MS number, suggesting that the terminal Ac-Glu-Val moiety was deattached by NE treatment but the cytotoxic MMAE remained attached to the antibody.

This trend clearly suggested the advantage of the exolinker over linear EVC-linker and highlighted the possibility that NE-induced off-target toxicity may have more pronounced deleterious effects than other premature payload release mechanisms, such as those driven by carboxylesterase.

## Conclusions

In summary, the introduction of exolinkers into ADCs suggests a promising shift in the field, addressing the fundamental issues associated with traditional Val-Cit linkers. Initial comparative studies showed that ADCs synthesized using the exolinker exhibited superior hydrophilic properties and significantly reduced aggregation. Further, the conjugation of the exolinker with established cytotoxic payloads such as MMAE and exatecan enhanced its potential, evidenced by the solubility of the constructs even in the absence of cosolvents. The universal applicability of the exolinker holds promise for the design of ADCs, including those that are typically hydrophobic. This potential was further validated via in vivo xenograft studies, in which the exolinker ADCs demonstrated enhanced antitumor efficacy, even at reduced doses, outperforming traditional ADCs. Specifically, ADCs with APL-1081, APL-1091, and APL-1092 payloads showed the ability to inhibit tumor growth more effectively than leading approved ADCs when normalized for the incorporated payloads. PK studies in rats provided insights into the transformative stability conferred by the exolinker in the ADC design. This exolinker design demonstrated the superior PK performance of exolinked ADCs in terms of payload retention relative to those with the traditional Val-Cit linker. In vitro assays using the exolinker also showed resistance to NE-mediated cleavage, confirming its position as a safer alternative. Overall, the exolinkers present a potential solution to the inherent issues associated with traditional linkers, offering improved therapeutic efficacy and safety profiles for ADCs.

## Experimental Section

### Compound Synthesis and Characterization

All solvents employed were commercially available anhydrous grade, and reagents were used as received unless otherwise noted. Compound purity of all compounds was assessed by HPLC and confirmed to be >95% purity. The LC–MS analysis was performed as previously reported. ^1^HNMR spectra were obtained with a Bruker 400 MHz Unity Avance400 MHz NMR instrument. Unless otherwise indicated, all protons were reported in DMSO-*d*_6_ solvent as parts per million (ppm) with respect to residual DMSO (2.50 ppm).

## Materials

Human IgG1 trastuzumab (Herceptin) was purchased from Roche Pharmaceutical Company (Switzerland). Trastuzumab-emutansine (Kadcyla) and trastuzumab deruxtecan (Enhertu) were purchased from WEP clinical (USA). Tri or tetra peptides for the exolinker were prepared by a solid-phase peptide synthesizer based on the previous report.^33^ The AJICAP peptide reagent was prepared based on the previous report.^34^ Trastuzumab-Mc-VC-PAB-MMAE (ADC 10), trastuzumab-AJICAP-Mc-VC-PAB-MMAE (ADC 11), and trastuzumab-linear-Mc-EVC-PAB-MMAE (ADC 12) were synthesized by the previously reported protocol.^28^ MC-VC-PAB-MMAE (CAS#: 646502-53-6) was purchased from NJ Biopharmaceuticals LLC (USA). MC-EVC-PAB-MMAE (CAS#: 2873452-49-2) was purchased from MedChemExpress (China). All other chemical reagents were purchased from Sigma-Aldrich (USA).

## Payload-Linker Synthesis

### Mal-Exo-EVC-Pyrene Synthesis

#### *tert*-Butyl (4*S*)-4-Acetamido-5-(((2*S*)-1-(((2*S*)-1-((4-(1-hydroxy-2-methoxy-2-oxoethyl)phenyl)amino)-1-oxo-5-ureidopentan-2-yl)amino)-3-methyl-1-oxobutan-2-yl)amino)-5-oxopentanoate (**S-1a**)

Ac-Glu(OtBu)-Val-Cit-OH (19.9 mg, 39.7 μmol) was dissolved in DMF (400 μL), and 1-[bis(dimethylamino)methylene]-1H-1, 2,3, triazolo[4,5-*b*]pyridinium 3-oxide hexafluorophosphate (18.1 mg, 47.6 μmol) and 2,4,6-trimethylpyridine (6.27 μL, 47.6 μmol) were added and stirred at room temperature (rt) for 10 min. Subsequently, *p*-amino-mandelic acid methyl ester^35^ (8.63 mg, 47.6 μmol) was added, and the mixture was stirred at rt for 21.5 h and then purified by reverse phase preparative chromatography. The fraction containing the product was collected, concentrated under reduced pressure to remove acetonitrile, and freeze-dried to obtain compound **S-1** (28.5 mg, quant).

^1^H NMR (400 MHz, DMSO-*d*_6_): δ 9.95 (s, 1H), 8.07 (d, *J* = 7.4 Hz, 1H), 7.99 (d, *J* = 8.0 Hz, 1H), 7.66 (d, *J* = 8.4 Hz, 1H), 7.50 (d, *J* = 8.4 Hz, 2H), 7.25 (d, *J* = 8.4 Hz, 2H), 5.92 (br s, 1H), 5.36 (br s, 2H), 5.01 (s, 1H), 4.34–4.29 (m, 1H), 4.26–4.20 (m, 1H), 4.14–4.10 (m, 1H), 3.53 (s, 3H), 3.00–2.83 (m, 2H), 2.18–2.13 (m, 2H), 1.94–1.89 (m, 2H), 1.84–1.23 (m, 17H), 0.79 (d, *J* = 6.8 Hz, 3H), 0.75 (d, *J* = 6.8 Hz, 3H).

MS (ESI) *m*/*z*: 665.30 [M + H]^+^.

##### *tert*-Butyl (4*S*)-4-Acetamido-5-(((2*S*)-3-methyl-1-(((2*S*)-1-((4-(5-methyl-3,6,9-trioxo-1-(pyren-1-yl)-7,10-dioxa-2,5-diazaundecan-8-yl)phenyl)amino)-1-oxo-5-ureidopentan-2-yl)amino)-1-oxobutan-2-yl)amino)-5-oxopentanoate (**S-2a**)

Compound **S-1a** (28.5 mg) was dissolved in DMF (430 μL) and stirred for 5 min at rt, and then bis(4-nitrophenyl carbonate 26.6 mg, 85.7 μmol) and DIPEA (11.1 μL, 64.4 μmol) were added, and the mixture was stirred at rt for 5 h. Then, after cooling with ice, sarcosine-pyrene^16^ (64.9 mg, 215 μmol), 1-hydroxybenzotriazole (8.7 mg, 64 μmol), and DIPEA (57.2 μL, 333 μmol) were added and stirred for 12 h. After the reaction, the crude product was purified by reversed phase preparative chromatography. Fractions containing the product were collected, concentrated under reduced pressure to remove acetonitrile, and freeze-dried to obtain pyrene **S-2a** (26.1 mg, 66%).

^1^H NMR (400 MHz, DMSO-*d*_6_): δ 10.09–10.07 (m, 1H), 8.63–8.60 (m, 1H), 8.33–7.65 (m, 13H), 7.60–7.57 (m, 2H), 7.37–7.32 (m, 2H), 5.94–5.91 (m, 1H), 5.72–5.70 (m, 1H), 5.37 (br s, 2H), 4.98–4.96 (m, 1H), 4.93–4.00 (m, 4H), 3.85–3.75 (m, 1H), 3.56–3.55 (m, 3H), 2.97–2.87 (m, 5H), 2.17–2.13 (m, 2H), 1.93–1.17 (m, 19H), 0.80–0.73 (m, 6H).

MS (ESI) *m*/*z*: 993.40 [M + H]^+^.

##### 2-(4-((*S*)-2-((*S*)-2-((*S*)-2-Acetamido-5-(*tert*-butoxy)-5-oxopentanamido)-3-methylbutanamido)-5-ureidopentanamido)phenyl)-2-((methyl(2-oxo-2-((pyren-1-ylmethyl)amino)ethyl)carbamoyl)oxy)acetic Acid (**S-3a**)

Pyrene **S-2a** (10.8 mg, 10.9 μmol) was dissolved in THF (700 μL) and water (400 μL) and stirred for 5 min under ice-cooling. 1 M lithium hydroxide aqueous solution (109 μL, 109 μmol) was added, and the mixture was stirred at rt for 1 h. After completion of the reaction, the pH was adjusted to about 6.0 using 0.1 M hydrochloric acid, and the product was purified by reverse phase preparative chromatography. Fractions containing the product were collected, concentrated under reduced pressure to remove acetonitrile, and freeze-dried to obtain pyrene **S-3a** (4.5 mg, 42%).

^1^H NMR (400 MHz, DMSO-*d*_6_): δ 13.05 (br s, 1H), 10.08–10.05 (m, 1H), 8.62–8.59 (m, 1H), 8.33–7.56 (m, 15H), 7.37–7.35 (m, 2H), 5.92 (br s, 1H), 5.61–5.60 (m, 1H), 5.36 (br s, 2H), 4.98–4.03 (m, 5H), 3.88–3.74 (m, 1H), 2.99–2.83 (m, 5H), 2.15–2.13 (m, 2H), 1.93–1.14 (m, 19H), 0.84–0.73 (m, 6H).

MS (ESI) *m*/*z*: 979.40 [M + H]^+^.

##### *tert*-Butyl (4*S*)-4-Acetamido-5-(((2*S*)-1-(((2*S*)-1-((4-(15-(2,5-dioxo-2,5-dihydro-1H-pyrrol-1-yl)-5-methyl-3,6,9-trioxo-1-(pyren-1-yl)-7-oxa-2,5,10-triazapentadecan-8-yl)phenyl)amino)-1-oxo-5-ureidopentan-2-yl)amino)-3-methyl-1-oxobutan-2-yl)amino)-5-oxopentanoate (**S-4a**)

Pyrene **S-3a** (3.7 mg, 3.8 μmol) was dissolved in DMF (400 μL) and cooled with ice, and DIPEA (1.9 μL, 11 μmol) and 1H-benzotriazole −1-yloxytripyrrolidinophosphonium hexafluorophosphate (2.9 mg, 5.6 μmol) were added. Next, *N*-(5-aminopentyl)maleimide hydrochloride (1.3 mg, 5.7 μmol) was added, and the mixture was warmed to rt and stirred for 2 h. After the completion of the reaction, the product was purified by reverse phase preparative chromatography. Fractions containing the product were collected, concentrated under reduced pressure to remove acetonitrile, and freeze-dried to obtain pyrene **S-4a** (1.3 mg, 29%).

^1^H NMR (400 MHz, DMSO-*d*_6_): δ 10.02–9.99 (m, 1H), 8.85–7.88 (m, 14H), 7.67–7.65 (m, 1H), 7.60–7.51 (m, 2H), 7.33–7.27 (m, 2H), 6.91–6.87 (m, 2H), 5.92–5.91 (m, 1H), 5.63–5.62 (m, 1H), 5.36 (br s, 2H), 5.07–4.92 (m, 2H), 4.35–3.76 (m, 5H), 3.18–3.14 (m, 1H), 2.99–2.83 (m, 7H), 2.17–2.13 (m, 2H), 1.95–1.89 (m, 1H), 1.85–1.72 (m, 4H), 1.66–1.45 (m, 3H), 1.40–1.17 (m, 15H), 1.05–1.01 (m, 2H), 0.83–0.73 (m, 6H).

MS (ESI) *m*/*z*: 1143.45 [M + H]^+^.

##### Mal-Exo-EVC-pyrene

1,4-Dioxane (380 μL) and 4 M HCl in 1,4-dioxane (95 μL, 380 μmol) were sequentially added to pyrene **S-4a** (2.2 mg, 1.9 μmol), and the mixture was stirred at rt for 4 h. After cooling with ice, DIPEA (71.8 μL, 418 μmol) was added, and the mixture was stirred at rt for 10 min. The reaction solution was purified by reversed-phase preparative chromatography; the fraction containing the product was collected and concentrated under reduced pressure to remove acetonitrile and then lyophilized to obtain Mal-Exo-EVC-pyrene (2.1 mg, 99%).

^1^H NMR (400 MHz, DMSO-*d*_6_): δ 12.06 (br s, 1H), 10.03–10.00 (m, 1H), 8.84–7.88 (m, 14H), 7.67–7.64 (m, 1H), 7.56–7.51 (m, 2H), 7.33–7.27 (m, 2H), 6.90–6.87 (m, 2H), 5.92–5.90 (m, 1H), 5.63–5.61 (m, 1H), 5.36 (br s, 2H), 5.08–4.96 (m, 2H), 4.35–3.76 (m, 5H), 3.18–3.14 (m, 1H), 2.97–2.83 (m, 7H), 2.20–2.16 (m, 2H), 1.93–1.88 (m, 1H), 1.81–1.78 (m, 4H), 1.69–1.53 (m, 3H), 1.35–1.17 (m, 6H), 1.08–1.01 (m, 2H), 0.81–0.73 (m, 6H).

MS (ESI) *m*/*z*: 1087.45 [M + H]^+^.

#### Mal-Exo-EEVC-pyrene Synthesis

##### *tert*-Butyl (6*S*,9*S*,12*S*,15*S*)-15-Acetamido-1-amino-12-(3-(*tert*-butoxy)-3-oxopropyl)-6-((4-(1-hydroxy-2-methoxy-2-oxoethyl)phenyl)carbamoyl)-9-isopropyl-1,8,11,14-tetraoxo-2,7,10,13-tetraazaoctadecan-18-oate (**S-1b**)

Ac-EEVC–OH (50.0 mg, 72.8 μmol) was dissolved in DMF (800 μL) and 1-[bis(dimethyl amino)methylene]-1H-1,2,3, triazolo[4,5-*b*]pyridinium 3-oxide hexafluorophosphate (33.2 mg, 87.4 μmol), 2,4,6-trimethylpyridine (11. Five μL, 87.4 μmol) was added and stirred at rt for 10 min. Subsequently, *p*-amino-mandelic acid methyl ester^35^ (15.8 mg, 87.4 μmol) was added, and the mixture was stirred at rt for 16 h, followed by purification by reverse phase preparative chromatography. Fractions containing the product were collected, concentrated under reduced pressure to remove acetonitrile, and lyophilized to obtain alcohol **S-11b** (54.0 mg, 87%).

^1^H NMR (400 MHz, DMSO-*d*_6_): δ 10.00 (s, 1H), 8.26–7.88 (m, 3H), 7.68–7.60 (m, 1H), 7.57 (d, *J* = 8.4 Hz, 2H), 7.32 (d, *J* = 8.4 Hz, 2H), 6.00–5.97 (m, 1H), 5.43 (br s, 2H), 5.08 (s, 1H), 4.40–4.37 (m, 1H), 4.32–4.19 (m, 3H), 3.60 (s, 3H), 3.09–2.90 (m, 2H), 2.25–2.18 (m, 4H), 2.03–1.53 (m, 10H), 1.46–1.36 (m, 20H), 0.86 (d, *J* = 6.8 Hz, 3H), 0.82 (d, *J* = 6.8 Hz, 3H).

MS (ESI) *m*/*z*: 850.40 [M + H]^+^.

##### *tert*-Butyl (6*S*,9*S*,12*S*,15*S*)-15-Acetamido-1-amino-12-(3-(*tert*-butoxy)-3-oxopropyl)-9-isopropyl-6-((4-(5-methyl-3,6,9-trioxo-1-(pyren-1-yl)-7,10-dioxa-2,5-diazaundecan-8-yl)phenyl)carbamoyl)-1,8,11,14-tetraoxo-2,7,10,13-tetraazaoctadecan-18-oate (**S-2b**)

Alcohol **S-1b** (50.3 mg, 59.2 μmol) was dissolved in DMF (650 μL) and stirred for 5 min under ice-cooling; then, bis(4-nitrophenyl) carbonate (54.0 mg, 178 μmol) and DIPEA (22.7 μL, 133 μmol) were added, and the mixture was stirred at rt for 5 h. Then, after ice cooling, sarcosin–pyrene^5^ (89.5 mg, 296 μmol), 1-hydroxybenzotriazole (12.0 mg, 88.8 μmol), and DIPEA (78.1 μL, 459 μmol) were added, and the mixture was brought to rt. The mixture was stirred for 18 h. After the reaction, the product was purified by reverse phase preparative chromatography. Fractions containing the product were collected, concentrated under reduced pressure to remove acetonitrile, and freeze-dried to obtain pyrene **S-22b** (34.0 mg, 57%).

^1^H NMR (400 MHz, DMSO-*d*_6_): δ 10.14–10.12 (m, 1H), 8.69–8.67 (m, 1H), 8.40–7.82 (m, 13H), 7.76–7.72 (m, 1H), 7.68–7.65 (m, 2H), 7.44–7.39 (m, 2H), 5.99 (br s, 1H), 5.79 (d, *J* = 6.4 Hz, 1H), 5.44 (br s, 2H), 5.05–4.97 (m, 2H), 4.44–4.08 (m, 4H), 3.93–3.80 (m, 1H), 3.63–3.62 (m, 3H), 3.05–2.92 (m, 5H), 2.25–2.14 (m, 4H), 2.00–1.28 (m, 30H), 0.87–0.80 (m, 6H).

MS (ESI) *m*/*z*: 1178.50 [M + H]^+^.

##### 2-(4-((2*S*,5*S*,8*S*,11*S*)-11-Acetamido-8-(3-(*tert*-butoxy)-3-oxopropyl)-5-isopropyl-16,16-dimethyl-4,7,10,14-tetraoxo-2-(3-ureidopropyl)-15-oxa-3,6,9-triazaheptadecanamido)phenyl)-2-((methyl(2-oxo-2-((pyren-1-ylmethyl)amino)ethyl)carbamoyl)oxy)acetic Acid (**S-3b**)

Pyrene **S-2b** (30.7 mg, 26.1 μmol) was dissolved in THF (2.25 mL) and water (0.75 mL) and stirred for 5 min under ice-cooling; then, lithium hydroxide monohydrate (5. Five mg, 0.13 mmol) was added, and the mixture was stirred at rt for 4 h. After completion of the reaction, the pH was adjusted to about 6.0 using 0.1 M hydrochloric acid, and the product was purified by reverse phase preparative chromatography. Fractions containing the product were collected, concentrated under reduced pressure to remove acetonitrile, and freeze-dried to obtain the pyrene **S-3b** mentioned above (17.2 mg, 57%).

^1^H NMR (400 MHz, DMSO-*d*_6_): δ 13.06 (br s, 1H), 10.13–10.10 (m, 1H), 8.69–8.66 (m, 1H), 8.40–7.85 (m, 13H), 7.77–7.73 (m, 1H), 7.67–7.64 (m, 2H), 7.44–7.42 (m, 2H), 5.99 (br s, 1H), 5.68–5.67 (m, 1H), 5.44 (br s, 2H), 5.05–4.96 (m, 2H), 4.46–4.10 (m, 4H), 3.92–3.81 (m, 1H), 3.05–2.90 (m, 5H), 2.25–2.14 (m, 4H), 2.03–1.29 (m, 30H), 0.88–0.80 (m, 6H).

MS (ESI) *m*/*z*: 1164.55 [M + H]^+^.

##### *tert*-Butyl (6*S*,9*S*,12*S*,15*S*)-15-acetamido-1-amino-12-(3-(*tert*-butoxy)-3-oxopropyl)-6-((4-(15-(2,5-dioxo-2,5-dihydro-1H-pyrrol-1-yl)-5-methyl-3,6,9-trioxo-1-(pyren-1-yl)-7-oxa-2,5,10-triazapentadecan-8-yl)phenyl)carbamoyl)-9-isopropyl-1,8,11,14-tetraoxo-2,7,10,13-tetraazaoctadecan-18-oate (**S-4b**)

Pyrene **S-3b** (14.7 mg, 12.6 μmol) was dissolved in DMF (1.0 mL) and cooled with ice; DIPEA (4.29 μL, 25.2 μmol) and 1H-benzotriazol-1-yloxytripyrrolidinophosphonium hexafluorophosphate (9.8 mg, 19 μmol) were added. Next, *N*-(5-aminopentyl)maleimide hydrochloride (4.1 mg, 19 μmol) was added, and the mixture was warmed to rt and stirred for 3.5 h. After completion of the reaction, the product was purified by reverse phase preparative chromatography. Fractions containing the product were collected, concentrated under reduced pressure to remove acetonitrile, and freeze-dried to obtain pyrene **S-44b** (7.0 mg, 73%).

^1^H NMR (400 MHz, DMSO-*d*_6_): δ 10.08–10.05 (m, 1H), 8.92–7.95 (m, 15H), 7.75–7.73 (m, 1H), 7.63–7.59 (m, 2H), 7.40–7.34 (m, 2H), 6.97–6.94 (m, 2H), 6.00–5.98 (m, 1H), 5.70–5.69 (m, 1H), 5.43 (br s, 2H), 5.14–5.01 (m, 2H), 4.45–4.38 (m, 1H), 4.32–3.83 (m, 5H), 3.25–3.20 (m, 1H), 3.10–2.90 (m, 7H), 2.25–2.18 (m, 4H), 2.08–1.24 (m, 34H), 1.12–1.04 (m, 2H), 0.88–0.81 (m, 6H).

MS (ESI) *m*/*z*: 1328.60 [M + H]^+^.

##### Mal-Exo-EEVC-pyrene

1,4-Dioxane (920 μL) and a 4 M hydrogen chloride/dioxane solution (230 μL, 918 μmol) were sequentially added to pyrene **S-4b** (6.1 mg, 4.6 μmol), and the mixture was stirred at rt for 4 h. After cooling with ice, DIPEA (172 μL, 1.10 mmol) was added and stirred at rt for 10 min. The reaction solution was purified by reversed-phase preparative chromatography; the fraction containing the product was collected, concentrated under reduced pressure to remove acetonitrile, and then lyophilized to obtain Mal-Exo-EEVC-pyrene (3.7 mg, 65%).

^1^H NMR (400 MHz, DMSO-*d*_6_): δ 12.04 (br s, 2H), 10.01–9.98 (m, 1H), 8.83–7.89 (m, 15H), 7.71–7.69 (m, 1H), 7.56–7.51 (m, 2H), 7.33–7.26 (m, 2H), 6.90–6.87 (m, 2H), 5.91–5.90 (m, 1H), 5.64–5.62 (m, 1H), 5.36 (br s, 2H), 5.08–4.92 (m, 2H), 4.35–4.32 (m, 1H), 4.25–3.76 (m, 5H), 3.18–3.14 (m, 1H), 2.99–2.82 (m, 7H), 2.21–2.15 (m, 4H), 1.93–1.17 (m, 16H), 1.05–1.01 (m, 2H), 0.82–0.74 (m, 6H).

MS (ESI) *m*/*z*: 1216.45 [M + H]^+^.

##### Mc-EVC-PAB-pyrene synthesis

Fmoc-Glu(OtBu)-Val-Cit-OH (100 mg, 147 μmol) was dissolved in *N*,*N*-dimethylformamide (733 μL). To this solution were added 1-[bis(dimethylamino)methylene]-1H-1,2,3-triazolo[4,5-*b*]pyridinium 3-oxid hexafluorophosphate (HATU) (66.9 mg, 176 μmol) and 2,4,6-trimethylpyridine (23.0 μL, 176 μmol), and the mixture was stirred at room temperature for 10 min. Subsequently, (4-aminophenyl)methanol (21.7 mg, 176 μmol) was added, and the reaction was allowed to stir at rt for an additional 3 h. The mixture was then purified by using reverse-phase column chromatography. Fractions containing the product were collected, solvent was removed under reduced pressure, and the residue was lyophilized to afford the desired alcohol (**S-5**) (51.1 mg, 64.9 μmol).

MS (ESI) *m*/*z*: 1573.9 [M + H]^+^.

S-5 (20.0 mg, 25.4 μmol) was dissolved in DMF (254 μL). Subsequently, bis(4-nitrophenyl) carbonate (23.2 mg, 76.2 μmol) and *N*,*N*-diisopropylethylamine (DIPEA) (9.70 μL, 57.2 μmol) were added, and the mixture was stirred at rt for 2 h. The reaction mixture was then cooled on ice, and sardine-pyrene (23.1 mg, 76.2 μmol) and *N*,*N*-diisopropylethylamine (DIPEA) (16.0 μL, 95.3 μmol) were added, followed by stirring at rt for 1 h. The reaction mixture was purified using normal-phase column chromatography. Fractions containing the product were collected, the solvent was removed under reduced pressure, and the residue was lyophilized to obtain the described pyrene (S-6) (16.2 mg, 14.5 μmol).

MS (ESI) *m*/*z*: 1115.5 [M + H]^+^.

Pyrene (**S-6**) (15.0 mg, 13.4 μmol) was dissolved in DMf (134 μL). Diethylamine (40.0 μL, 403 μmol) was then added, and the mixture was stirred at rt for 3 h. After the completion of the reaction, it was purified using reverse-phase column chromatography. Fractions containing the product were collected, the solvent was removed under reduced pressure, and the residue was lyophilized to obtain the described pyrene (**S-7**) (9.00 mg, 10.1 μmol).

##### MS (ESI) *m*/*z*: 893.4 [M + H]^+^.

Pyrene (**S-7**) (8.00 mg, 8.96 μmol) was dissolved in DMF (89.6 μL). *N*,*N*-Diisopropylethylamine (3.00 μL, 17.9 μmol), and 1H-benzotriazol-1-yloxytripyrrolidinophosphonium hexafluorophosphate (6.99 mg, 13.4 μmol) were then added. Subsequently, 6-maleimidohexanoic acid (2.84 mg, 13.4 μmol) was added, and the mixture was stirred at rt for 3 h. Acetonitrile (276 μL) was added followed by the addition of TFA (11.3 μL, 1.6 mmol) under ice cooling, and the mixture was stirred at rt for 0.5 h. After completion of the reaction, it was purified using reverse-phase column chromatography. The fractions containing the product were collected and concentrated under a reduced pressure. After acetonitrile was removed, lyophilization yielded the described Mc-EVC-PAB-pyrene (0.94 mg, 0.91 μmol).

MS (ESI) *m*/*z*: 1030.5 [M + H]+.

##### Mc-VC-PAB-pyrene synthesis.

Mc-VC–PAB-PNP (15.5 mg, 0.021 mmol) was dissolved in dichloromethane (1 mL), and DMF (0.025 mL, 0.142 mmol), sarcosine-pyrene^5^ (7.6 mg, 0.025 mmol), and a DMF solution (0.5 mL) were added and stirred for 17 h. After purification by reversed-phase preparative chromatography, the fraction containing the product was collected, concentrated under reduced pressure to remove acetonitrile, and lyophilized to obtain Mc-VC-PAB-pyrene (7.3 mg, 38%)

MS (ESI) *m*/*z*: 901.4 [M + H]^+^.

#### APL-1091 (Mal-Exo-EEVC-MMAE) Synthesis

##### *tert*-Butyl (6*S*,9*S*,12*S*,15*S*)-15-acetamido-1-amino-12-(3-(*tert*-butoxy)-3-oxopropyl)-9-isopropyl-6-((4-(2-methoxy-1-(((4-nitrophenoxy)carbonyl)oxy)-2-oxoethyl)phenyl)carbamoyl)-1,8,11,14-tetraoxo-2,7,10,13-tetraazaoctadecan-18-oate (**S-8**)

Compound **S-1b** (140 mg, 0.165 mmol) and bis(4-nitrophenyl) carbonate (108 mg, 0.355 mmol) were weighed in a dry flask, dissolved in anhydrous DMF (4 mL), and treated with DIPEA (100 mL, 0.574 mmol). The flask was sealed with a rubber septum under a nitrogen balloon, and the reaction mixture was stirred at rt for 18 h. The DMF was evaporated off in vacuo, and the reaction mixture was dissolved in 1:1 MeCN:H_2_O with a trace of formic acid. The mixture was purified by reverse-phase flash chromatography on a C18 silica gel column (gradient elution, 20–90% MeCN in H_2_O, 0.05% FA both phases), and the cleanest product fractions were combined, partially concentrated in vacuo, frozen on dry ice, and lyophilized to yield compound **S-8** as a white solid (130 mg, 0.128 mmol, 78% yield).

MS (ESI) *m*/*z*: 1015.6 [M + H]^+^.

##### Methyl (3*R*,4*S*,7*S*,10*S*)-14-(4-((2*S*,5*S*,8*S*,11*S*)-11-Acetamido-8-(3-(*tert*-butoxy)-3-oxopropyl)-5-isopropyl-16,16-dimethyl-4,7,10,14-tetraoxo-2-(3-ureidopropyl)-15-oxa-3,6,9-triazaheptadecanamido)phenyl)-4-((*S*)-*sec*-butyl)-3-(2-((*S*)-2-((1*R*,2*R*)-3-(((1*S*,2*R*)-1-hydroxy-1-phenylpropan-2-yl)amino)-1-methoxy-2-methyl-3-oxopropyl)pyrrolidin-1-yl)-2-oxoethyl)-7,10-diisopropyl-5,11-dimethyl-6,9,12-trioxo-2,13-dioxa-5,8,11-triazapentadecan-15-oate (**S-9a**)

**S-8** (85 mg, 0.084 mmol) and HOBT(20 mg, 0.13 mmol) were dissolved in anhydrous DMF (2 mL) and then treated with a solution of monomethylauristatin E (MMAE, 63 mg, 0.088 mmol) in anhydrous DMF (2 mL). The reaction was then treated with DIPEA (75 mL, 0.43 mmol), and the flask was purged with nitrogen, sealed with a rubber septum under a nitrogen balloon, and stirred at room temperature overnight. After 23 h, the reaction mixture was concentrated in vacuo and dissolved in 1:1 MeCN:H_2_O with trace FA, and the crude material purified by reverse-phase flash chromatography on a C18 silica gel column (gradient elution, 20–80% MeCN in H_2_O, 0.05% FA both phases). The cleanest product fractions by LC–MS were combined, partially concentrated in vacuo, frozen on dry ice, and lyophilized to yield **S-9a** as a white solid (94 mg, 0.059 mmol, 71% yield).

MS (ESI) *m*/*z*: 1593.6 [M + H]^+^.

##### (3*R*,4*S*,7*S*,10*S*)-14-(4-((2*S*,5*S*,8*S*,11*S*)-11-Acetamido-8-(3-(*tert*-butoxy)-3-oxopropyl)-5-isopropyl-16,16-dimethyl-4,7,10,14-tetraoxo-2-(3-ureidopropyl)-15-oxa-3,6,9-triazaheptadecanamido)phenyl)-4-((*S*)-*sec*-butyl)-3-(2-((*S*)-2-((1*R*,2*R*)-3-(((1*S*,2*R*)-1-hydroxy-1-phenylpropan-2-yl)amino)-1-methoxy-2-methyl-3-oxopropyl)pyrrolidin-1-yl)-2-oxoethyl)-7,10-diisopropyl-5,11-dimethyl-6,9,12-trioxo-2,13-dioxa-5,8,11-triazapentadecan-15-oic Acid (**S-10a**)

A solution of **S-9a** (94 mg, 0.059 mmol) in tetrahydrofuran (THF, 5 mL) and water (2 mL) was cooled to 0 °C under a nitrogen balloon. After a few minutes, 1.0 M aqueous lithium hydroxide (0.60 mL, 0.60 mmol) was added and the reaction mixture was stirred at 0 °C for 50 min. The reaction was acidified to ca. pH 5 with 5% aqueous hydrochloric acid and saturated sodium bicarbonate and then briefly concentrated in vacuo to remove the THF. MeCN (ca. 2 mL) was added, and the mixture was purified by reverse-phase flash chromatography on a C18 silica gel column (gradient elution, 20–90% MeCN in H_2_O, 0.05% FA both phases). The cleanest product fractions were combined, partially concentrated in vacuo, frozen on dry ice, and lyophilized to yield **S-10a** as a white solid (77 mg, 0.049 mmol, 83% yield).

MS (ESI) *m*/*z*: 1579.7 [M + H]^+^.

##### *tert*-Butyl (6*S*,9*S*,12*S*,15*S*)-15-Acetamido-1-amino-12-(3-(*tert*-butoxy)-3-oxopropyl)-6-((4-((3*R*,4*S*,7*S*,10*S*)-4-((*S*)-*sec*-butyl)-21-(2,5-dioxo-2,5-dihydro-1H-pyrrol-1-yl)-3-(2-((*S*)-2-((1*R*,2*R*)-3-(((1*S*,2*R*)-1-hydroxy-1-phenylpropan-2-yl)amino)-1-methoxy-2-methyl-3-oxopropyl)pyrrolidin-1-yl)-2-oxoethyl)-7,10-diisopropyl-5,11-dimethyl-6,9,12,15-tetraoxo-2,13-dioxa-5,8,11,16-tetraazahenicosan-14-yl)phenyl)carbamoyl)-9-isopropyl-1,8,11,14-tetraoxo-2,7,10,13-tetraazaoctadecan-18-oate (**S-11a**)

^1^H-Benzotriazol-1-yloxytripyrrolidinophosphonium hexafluorophosphate (PyBOP, 87 mg, 0.17 mmol) and *N*-(5-aminopentyl)maleimide HCl salt (35 mg, 0.16 mmol) were weighed to a flask containing **S-10a** (77 mg, 0.049 mmol), and all were dissolved in anhydrous DMF (3 mL). DIPEA (50 mL, 0.29 mmol) was added, the flask was sealed with a rubber septum under a nitrogen balloon, and the reaction was stirred at room temperature for 6 h. DMF was evaporated in vacuo, and the reaction was dissolved in 1:1 MeCN:H_2_O with a trace of FA. The mixture was purified by reverse-phase flash chromatography on a C18 silica gel column (gradient elution, 20–80% MeCN in H_2_O, 0.05% FA both phases), and the cleanest product fractions were combined, partially concentrated in vacuo, frozen on dry ice, and lyophilized to yield **S-11a** as a white solid (65 mg, 0.037 mmol, 76%).

MS (ESI) *m*/*z*: 1744.7 [M + H]^+^.

##### (6*S*,9*S*,12*S*,15*S*)-15-Acetamido-1-amino-6-((4-((3*R*,4*S*,7*S*,10*S*)-4-((*S*)-*sec*-butyl)-21-(2,5-dioxo-2,5-dihydro-1*H*-pyrrol-1-yl)-3-(2-((*S*)-2-((1*R*,2*R*)-3-(((1*S*,2*R*)-1-hydroxy-1-phenylpropan-2-yl)amino)-1-methoxy-2-methyl-3-oxopropyl)pyrrolidin-1-yl)-2-oxoethyl)-7,10-diisopropyl-5,11-dimethyl-6,9,12,15-tetraoxo-2,13-dioxa-5,8,11,16-tetraazahenicosan-14-yl)phenyl)carbamoyl)-12-(2-carboxyethyl)-9-isopropyl-1,8,11,14-tetraoxo-2,7,10,13-tetraazaoctadecan-18-oic Acid (**APL-1091**)

**S-11a** (65 mg, 0.037 mmol) was treated with MeCN (2 mL) and 85% aqueous phosphoric acid (1.00 mL, 14.6 mmol), the flask was sealed with a rubber septum under a nitrogen balloon, and the reaction mixture was stirred at ambient temperature. After 5 h, water (2 mL) was added, and the mixture was purified by reverse-phase flash chromatography on a C18 silica gel column (gradient elution, 20–80% MeCN in H_2_O, 0.05% FA both phases). The cleanest product fractions were combined, partially concentrated in vacuo, frozen on dry ice, and lyophilized to give APL-1091 as a white solid (49 mg, 0.030 mmol, 80% yield).

MS (ESI) *m*/*z*: 1631.6 [M + H]^+^.

The synthesis procedure for APL-1081 was followed by that of its sister compounds APL-1091 and Mc-Exo-EVC-pyrene.

#### APL-1092 (Mal-Exo-EEVC-Exatecan) Synthesis

##### *tert*-Butyl (6*S*,9*S*,12*S*,15*S*)-15-Acetamido-1-amino-12-(3-(*tert*-butoxy)-3-oxopropyl)-6-((4-(1-((((1*S*,9*S*)-9-ethyl-5-fluoro-9-hydroxy-4-methyl-10,13-dioxo-2,3,9,10,13,15-hexahydro-1*H*,12*H*-benzo[de]pyrano[3′,4’:6,7]indolizino[1,2-*b*]quinolin-1-yl)carbamoyl)oxy)-2-methoxy-2-oxoethyl)phenyl)carbamoyl)-9-isopropyl-1,8,11,14-tetraoxo-2,7,10,13-tetraazaoctadecan-18-oate (**S-9b**)

**S-8** (67 mg, 0.066 mmol) and HOBt (17 mg, 0.11 mmol) were dissolved in anhydrous DMF (2 mL) and then treated with a suspension of Exatecan mesylate (35 mg, 0.066 mmol) in anhydrous DMF (2 mL). The reaction was then treated with DIPEA (50 mL, 0.29 mmol), and the flask was purged with nitrogen, sealed with a rubber septum under a nitrogen balloon, and stirred at room temperature. After 4 h, the reaction mixture was concentrated in vacuo and dissolved in 1:1 MeCN:H_2_O with trace FA, and the crude material was purified by reverse-phase flash chromatography on a C18 silica gel column (gradient elution, 20–80% MeCN in H_2_O, 0.05% FA both phases). The cleanest product fractions by LC–MS were combined, partially concentrated in vacuo, frozen on dry ice, and lyophilized to yield **S-9b** as a white solid (57 mg, 0.043 mmol, 66% yield).

MS (ESI) *m*/*z*: 1311.7 [M + H]^+^.

##### 2-(4-((2*S*,5*S*,8*S*,11*S*)-11-Acetamido-8-(3-(*ter*-butoxy)-3-oxopropyl)-5-isopropyl-16,16-dimethyl-4,7,10,14-tetraoxo-2-(3-ureidopropyl)-15-oxa-3,6,9-triazaheptadecanamido)phenyl)-2-(1S,9S)-9-ethyl-5-fluoro-9-hydroxy-4-methyl-10,13-dioxo-2,3,9,10,13,15-hexahydro-1*H*,12*H*-benzo[de]pyrano[3′,4’:6,7]indolizino[1,2-b]quinolin-1-yl)carbamoyl)oxy)acetic Acid (**S-10b**)

A solution of **S-9b** (57 mg, 0.043 mmol) in THF (3 mL) and water (1.5 mL) was cooled to 0 °C under a nitrogen balloon. After a few minutes, 1.0 M aqueous lithium hydroxide (0.50 mL, 0.50 mmol) was added and the reaction was stirred at 0 °C for ca. 1 h. The reaction was acidified to ca. pH 5 with 5% aqueous hydrochloric acid and saturated sodium bicarbonate and then briefly concentrated in vacuo to remove the THF. MeCN (ca. 2 mL) was added, and the mixture was briefly sonicated to dissolve the solids and then purified by reverse-phase flash chromatography on an oversized C18 silica gel column (gradient elution, 20–90% MeCN in H_2_O, 0.05% FA both phases). The two products did not separate well; therefore, all product fractions were combined, partially concentrated in vacuo, frozen on dry ice, and lyophilized to yield **S-10b** as an impure white solid (45 mg, 0.035 mmol, 80% yield).

MS (ESI) *m*/*z*: 1297.6 [M + H]^+^.

##### <i>tert</i>-Butyl (6<I>S</I>,9<I>S</I>,12<I>S</I>,15<I>S</I>)-15-Acetamido-1-amino-12-(3-(<i>tert</i>-butoxy)-3-oxopropyl)-6-((4-(2-((5-(2,5-dioxo-2,5-dihydro-1H-pyrrol-1-yl)pentyl)amino)-1-((((1S,9S)-9-ethyl-5-fluoro-9-hydroxy-4-methyl-10,13-dioxo-2,3,9,10,13,15-hexahydro-1<I>H</I>,12<I>H</I>-benzo[de]pyrano[3′,4’:6,7]indolizino[1,2-<i>b</i>]quinolin-1-yl)carbamoyl)oxy)-2-oxoethyl)phenyl)carbamoyl)-9-isopropyl-1,8,11,14-tetraoxo-2,7,10,13-tetraazaoctadecan-18-oate (<b>**S-10b**)

^1^H-Benzotriazol-1-yloxytripyrrolidinophosphonium hexafluorophosphate (PyBOP, 55 mg, 0.11 mmol) and *N*-(5-aminopentyl)maleimide HCl salt (22 mg, 0.10 mmol) were weighed to a flask containing **S-10b** (45 mg, 0.035 mmol), and all were dissolved in anhydrous DMF (3 mL). DIPEA (30 mL, 0.17 mmol) was added, the flask was sealed with a rubber septum under a nitrogen balloon, and the reaction was stirred at rt for 18 h. DMF was evaporated in vacuo and the reaction was dissolved in 1:1 MeCN:H_2_O with a trace of FA. The mixture was purified by reverse-phase flash chromatography on a C18 silica gel column (gradient elution, 20–80% MeCN in H_2_O, 0.05% FA both phases), and the cleanest product fractions were combined, partially concentrated in vacuo, frozen on dry ice, and lyophilized to yield **S-11b** as a white solid (22 mg, 0.015 mmol, 43%).

MS (ESI) *m*/*z*: 1462.7 [M + H]^+^.

##### APL-1092.

S-11b (22 mg, 0.015 mmol) was treated with MeCN (1 mL) and 85% aqueous phosphoric acid (1.00 mL, 14.6 mmol), the flask was sealed with a rubber septum under a nitrogen balloon, and the reaction was stirred at ambient temperature. After 1.5 h, water (ca. 1 mL) was added, and the mixture was purified by reverse-phase flash chromatography on a C18 silica gel column (gradient elution, 10–70% MeCN in H_2_O, 0.05% FA both phases). The cleanest product fractions were combined, partially concentrated in vacuo, frozen on dry ice, and lyophilized to yield APL-1092 as a white solid (18.8 mg, 0.0139 mmol, 92% yield), which appeared to be a mixture of stereoisomers obtained by LC–MS.

MS (ESI) *m*/*z*: 1349.2 [M + H]^+^.

#### ADC Synthesis

##### General procedure for DAR = 8 ADC Synthesis

Trastuzumab (1 mg) was dissolved in water and then buffer exchanged into conjugation buffer (0.2 mL, pH 7.5, 50 mM PBS, 10 mM EDTA) to prepare for the conjugation process. The reduction reaction began with the addition of 5 eq. Tris(2-carboxyethyl)-phosphine hydrochloride (TCEP-HCl) to the antibody and stirred mildly for 1.5 h at 37 °C. The resulting reaction mixture, DMA (8% v/v, not needed for APL-1091 or APL-1092), and 10 eq. of payload-linker were sequentially added and stirred mildly for 1 h at 20 °C. The unreacted drug linker was quenched with the addition of 25 eq of *N*-acetyl-l-cysteine (NAC) and mixed for 25 min at 20 °C. The final mixture was purified using NAP-5 desalting columns and eluted with pH 5.2, 20 mM histidine, 5.5% trehalose.

##### General procedure for DAR = 2 AJICAP-ADC Synthesis

2.5 equiv of AJICAP peptide reagent (20 mM in DMF) was added to trastuzumab (10 mg/mL, 20 mM acetate buffer, pH 5.5), and then, it was allowed to incubate for 1 h at 20 °C. After 1 h had passed, excess NH_2_OH HCl was introduced, and the mixture was left to be stirred for an additional 1 h. The reaction mixture was then purified using a NAP-25 desalting column and was eluted with a 20 mM acetate buffer at pH 5.5. The resulting trastuzumab-Lys248 thiol and 2.7 equiv of APL-1091 or APL-1092 were sequentially added and stirred mildly for 1 h at 20 °C. The unreacted drug linker was quenched with the addition of 25 equiv of *N*-acetyl-l-cysteine (NAC) and mixed for 25 min at 20 °C. The final mixture was purified using NAP-5 desalting columns and eluted with pH 5.2, 20 mM histidine, and 5.5% trehalose.

##### Instruments and Analytical methods

The ADC concentration and recovery were measured using the slope spectroscopy method with a Solo-VPE system.^25^

HIC-HPLC analysis was performed as previously reported.^36^

SEC-HPLC analysis of ADCs was performed using a Waters ACQUITY UPLC Protein BEH SEC column (200 Å, 4.6 × 300 mm, 1.7 μm) as previously reported.^36^

##### In vivo Xenograft Study

Cells.

NCI–N87 cells (Cat # CRL-5822) were purchased through ATCC. Cells were cultured by a previously established procedure.^34^

Animals.

NOD.CB17 homozygous mice were procured, fed, and housed by a previously established procedure.^34^

Implantation.

Implantation was performed as previously reported.^34^

Study Arms and Treatments for NCI–N87.

Tumor volumes were monitored, and on the first day (when mean tumor volume reached ∼120 mm^3^), mice were stratified and placed into 3 treatment groups of (10) mice as outlined in Tables 3 and 4.

Treatments were administered by tail vein injection (100 μL volumes). Doses were administered on days 0, 4, 7, and 11 for a total of 4 doses for the study. Animal weights and tumor volumes were measured.

#### Rat PK Study

##### Animal Experiments

Animal experiments were cultured by a previously established procedure.^34^

##### Clinical Observations

The animals were observed once a day for clinical signs. Individual body weights were measured on days 0, 7, 14, and 21 with the first day of administration defined as day 0.

##### Total Antibody Analysis

The blood samples were collected at 6 time points (immediately after administration, after administration, and 1, 3, 7, 14, and 21 days after administration) via the caudal vein. The concentrations of total antibody were measured by the double sandwich ELISA method.

##### LC–MS Assay for Total ADC Analysis

A 25 μL portion of Dynabeads M-280 Streptavidin was washed with PBS buffer (pH 7.4). Then, 50 μL of human IgG-Fc fragment antibody (1 mg/mL, PBS buffer, Bethyl Laboratories, Inc., USA) was added. After being shaken at rt for 2 h, the beads were washed with PBS buffer (pH 7.4) five times. The blood sample was added to the beads and shaken for an additional 2 h. The beads were then washed with PBS buffer (pH 7.4) five times, and elution solution (25 μL, 10% ACN, 1% formic acid v/v in water) was added to each sample. After the mixture was shaken for 30 min, the eluent was injected for LC–MS analysis.

The resulting sample was analyzed by using a NexeraBio system (Shimadzu, Kyoto, Japan) coupled to a timsTOF Pro mass spectrometer (Bruker Daltonics, Bremen, Germany). A PLRP-S column (5 μm, 2.1 × 50 mm, 1000 Å (Agilent Technologies, Santa Clara, CA)) was used for the analytical column with a column temperature of 80 °C. The autosampler temperature was set at 4 °C, and the injection volume was 25 μL. Mobile phase A was 0.1% formic acid in water, and mobile phase B was 0.1% formic acid in acetonitrile. The gradient conditions of B % were: 0–0.5 min hold at 10% and 0.5–1.0 min linear gradient from 10% to 25%. After 3 min of holding at 25%, a linear gradient from 25% to 90% in 6 min was performed. A wash step was conducted as a 0.1 min linear gradient from 90% to 98%, holding for 5 min at 98%. Subsequently, mobile phase B was reduced to 10% within 0.1 min and maintained at 10% for 4.8 min. The flow rate was 400 μL/min. MS analysis was conducted in the positive ion mode with a scan range of *m*/*z* 500–6000, and TIMS was set to unable. The end plate offset and the capillary voltage were set to 500 V and 4500 V, respectively. The nebulizer gas was 1.4 bar and 5.5 L/min and the dry gas temperature was 200 °C. All samples were analyzed with an in-source collision-induced dissociation (isCID) energy of 100 eV.

The resulting data were processed and analyzed with Genedata Expressionist software version 16.5. One (Genedata, Basel, Switzerland). The spectra of retention time 5.1–6.1 min and *m*/*z* 2300–4400 were extracted and analyzed with using a time-resolved deconvolution and the Maximum Entropy (MaxEnt) algorithm. Outputs of minimum and maximum masses were, respectively, set to 145 and 160 kDa with a 1.0 Da mass step. Appropriate smoothing was conducted before the peak detection node. After valid feature filtration of intensity and repetition of detection, protein mapping was conducted with a mass tolerance of 500 ppm, and each linker and payload was set for fixed modifications or conjugates. Each peak height was provided, and the sum of the top 3 glycan variants (2 × A2G0F, A2G0F/A2G1F, and 2 × A2G1F) was used for DAR calculation.

#### In Vitro Human Neutrophil Assay

##### Neutrophil Elastase Treatment

NE treatment was performed according to the previously reported method^9,10^ with slight modifications. 7.5 μL of ADC solution (2.66 μM, PBS, pH 7.4) was mixed with 92.5 μL of tris-buffered saline (TBS, pH 7.5) and 100 μL of human NE (20 μg/mL, TBS, pH 7.5) (Enzo Life Sciences, USA). The mixture was incubated at 37 °C for 24 h. Then, this mixture was employed for the in vitro cytotoxicity assay.

##### Cell Culture

The MCF-7 cell line was obtained from Japanese Collection of Research Bioresources Cell Bank (Japan). The SKBR-3 cell line was obtained from Memorial Sloan Kettering Cancer Center (USA). Both cells were cultured on a Collagen I-coated cell culture dish (IWAKI, Japan) in the culture medium (DMEM supplemented with 10% FBS and 1% penicillin–streptomycin) in 5% CO_2_ at 37 °C.

##### Cytotoxicity Assay

In vitro cytotoxicity assay was performed according to the previously reported method^32^ with slight modifications. Five μL of 100 nM NE-treated or untreated ADCs or MMAE in NE-reaction buffer was mixed with 45 μL of culture medium and serially 3- or 4-fold diluted by the culture medium. 1000 cells of MCF-7 and SKBR-3 cells were seeded onto a Collagen I-coated 96-well cell culture plate (IWAKI, Japan) with 50 μL of culture medium and cultured for 24 h in 5% CO_2_ at 37 °C. Diluted ADCs were added to each cell and cultured for 6 days. Relative cell numbers were evaluated using CellTiter-Glo Luminescent Cell Viability Assay (Promega) following the manufacturer’s protocol. Luminescence was analyzed using a Nivo plate reader (PerkinElmer).

#### Mouse Plasma Stability Assay

##### Mouse Plasma Treatment

A solution was prepared by spiking ADvC into 500 μL of mouse plasma (Charles River Laboratories) until a definitive concentration of 0.1 mg/mL was reached. Subsequently, sterile filtration of the mixture was performed. The prepared solution was then carefully aliquoted into six separate Eppendorf tubes, with 50 μL dispensed into each. Three of these aliquots were incubated at a controlled temperature of 37 °C for 4 days. Concurrently, the remaining three tubes were stored at −80 °C for the same period (these samples were designated as the day 0 samples.). After these periods, 100 μL of acetonitrile was added to each sample. Once thoroughly vortex-mixed, the samples were centrifuged, and the pellet was isolated. The resulting supernatants from each tube were then collected and subsequently subjected to advanced LC–MS analysis.

#### LC–MS Analysis

The quantification of the released payload from the ADC was conducted using RP-HPLC. The fluorescence of the detached payload relatives detected from the day 4 and day 0 samples was determined using the extracted ion chromatogram. The difference between these values was analyzed to deduce payload release. Separately, using free pyrene, a correlation of fluorescence wavelength area on HPLC and concentration was established. Using this correlation equation, the fluorescence strength of each ADC sample was converted into concentration. The percentage difference in the ion chromatogram using day 0 concentration as the 100% benchmark was calculated to determine the payload release rate.

### Cathepsin B Cleavage Assay for ADCs

Cathepsin B cleavage assay was cultured by a previously reported procedure.^16^

## Supporting Information Available

Details of analytical results including NMR, LC–MS and QTOF MS, rat PK, and NE assay (PDF)
SI2 (CSV)

## Abbreviations USED

ADC: antibody–drug conjugate
AJICAP: AJICAP (site-specific antibody–drug conjugation technology)
DAR: drug–antibody ratio
DMEM: Dulbecco’s modified Eagle medium
DMF: dimethylformamide
ELISA: enzyme-linked immunosorbent assay
EVC: Glu-Val-Cit (glutamic acid-valine-citrulline)
FA: formic acid
FBS: fetal bovine serum
FDA: U.S. Food and Drug Administration
HIC: hydrophobic interaction chromatography
LC–MS: liquid chromatography–mass spectrometry
mAb: monoclonal antibody
NAC: *N*-acetyl-l-cysteine
NE: neutrophil elastase
PAB: *p*-aminobenzylcarbamate
PBS: phosphate-buffered saline
PEG: polyethylene glycol
PK: pharmacokinetics
Q-TOF/MS: quadrupole time-of-flight mass spectrometry
RPMI: Roswell Park Memorial Institute medium
SEC: size-exclusion chromatography
TBS: tris-buffered saline
TCEP-HCl: tris(2-carboxyethyl)phosphine hydrochloride
TFA: trifluoroacetic acid
THF: tetrahydrofuran
Val-Cit: valine-citrulline
