# Supplementary material for: Exo-Cleavable Linkers: Enhanced Stability and Therapeutic Efficacy in Antibody–Drug Conjugates
Source: J Med Chem. 2024 Oct 16;67(20):18124–38. doi: 10.1021/acs.jmedchem.4c01251 (PMC11513888; doi:10.1021/acs.jmedchem.4c01251)
Supplement: Supplementary file 1 — jm4c01251_si_001.pdf [file jm4c01251_si_001.pdf]

Supporting information

Exo-Cleavable Linkers: Enhanced Stability and Therapeutic Efficacy in Antibody-Drug Conjugates

Tomohiro Watanabe[†1], Naoko Arashida, Tomohiro Fujii[†], Natsuki Shikida[†], Kenichiro Ito[†], Kazutaka Shimbo[†], Takuya Seki[†], Yusuke Iwai[†], Ryusuke Hirama[†], Noriko Hatada[†], Akira Nakayama[†], Tatsuya Okuzumi[†], Yutaka Matsuda*[‡1]

* Corresponding author

YM: Yutaka.Matsuda@US.AjiBio-Pharma.com

[1] These authors contributed equally.

[†]Ajinomoto Co., Inc., 1-1, Suzuki-Cho, Kawasaki-Ku, Kawasaki-Shi, Kanagawa 210-8681, Japan.

[‡]Ajinomoto Bio-Pharma Services, 11040 Roselle Street, San Diego, CA 92121, United States.

Table of Content

Analytical results

1 Payload-linker synthesis: HPLC, LC-MS and NMR S–3

2 ADC synthesis: HIC and SEC analysis S–38

3 *In vivo* xenograft study S–42

4 Rat PK study S–45

5 *In vitro* human neutrophil assay S–72

6 Comparison summary of linear and Exo-EVC Linkers S–73

1 Payload-linker synthesis: HPLC, LC-MS, and 1H NMR

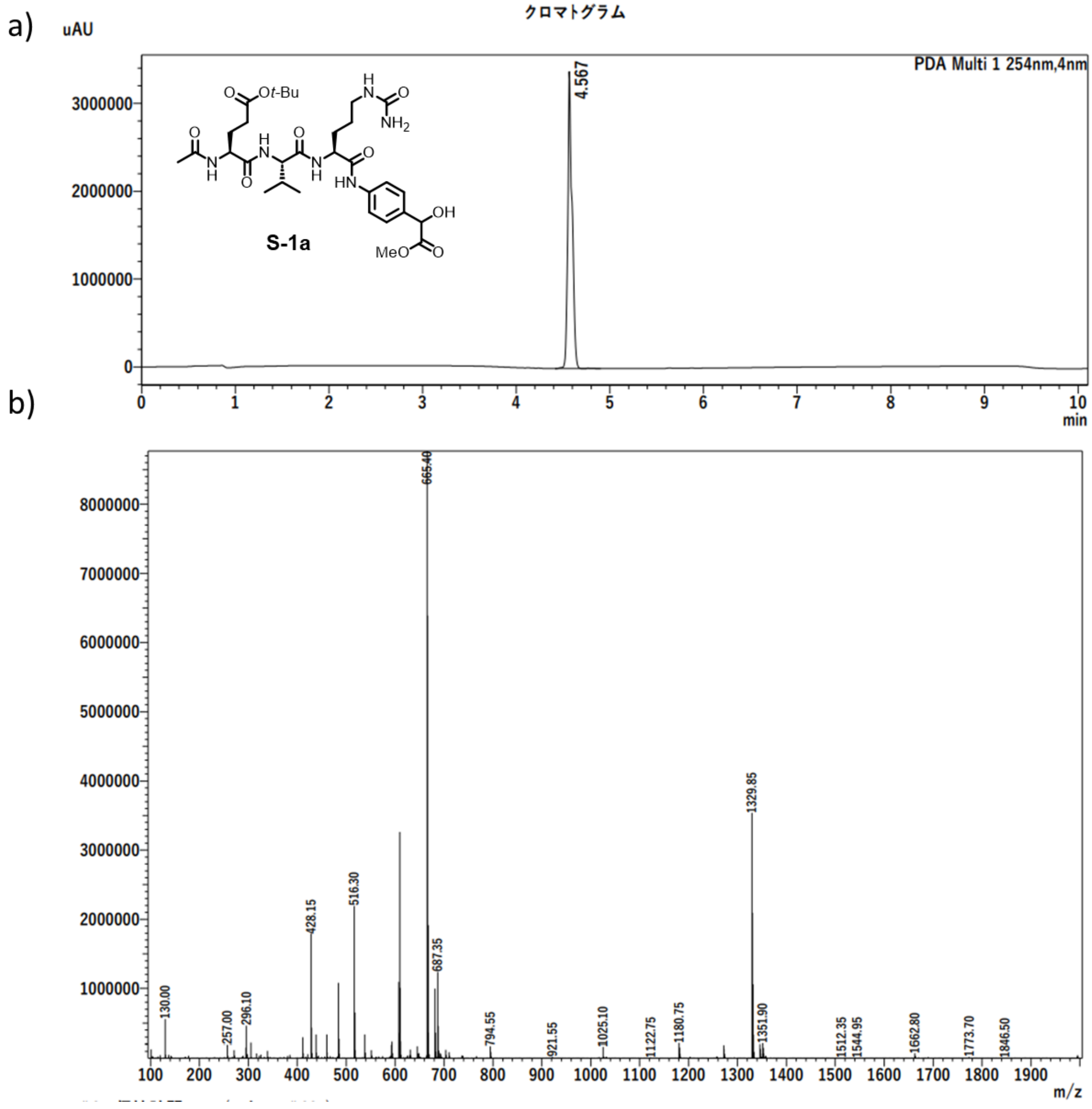

Figure S1. Analysis of compound **S-1a**, a) HPLC analysis, b) MS analysis

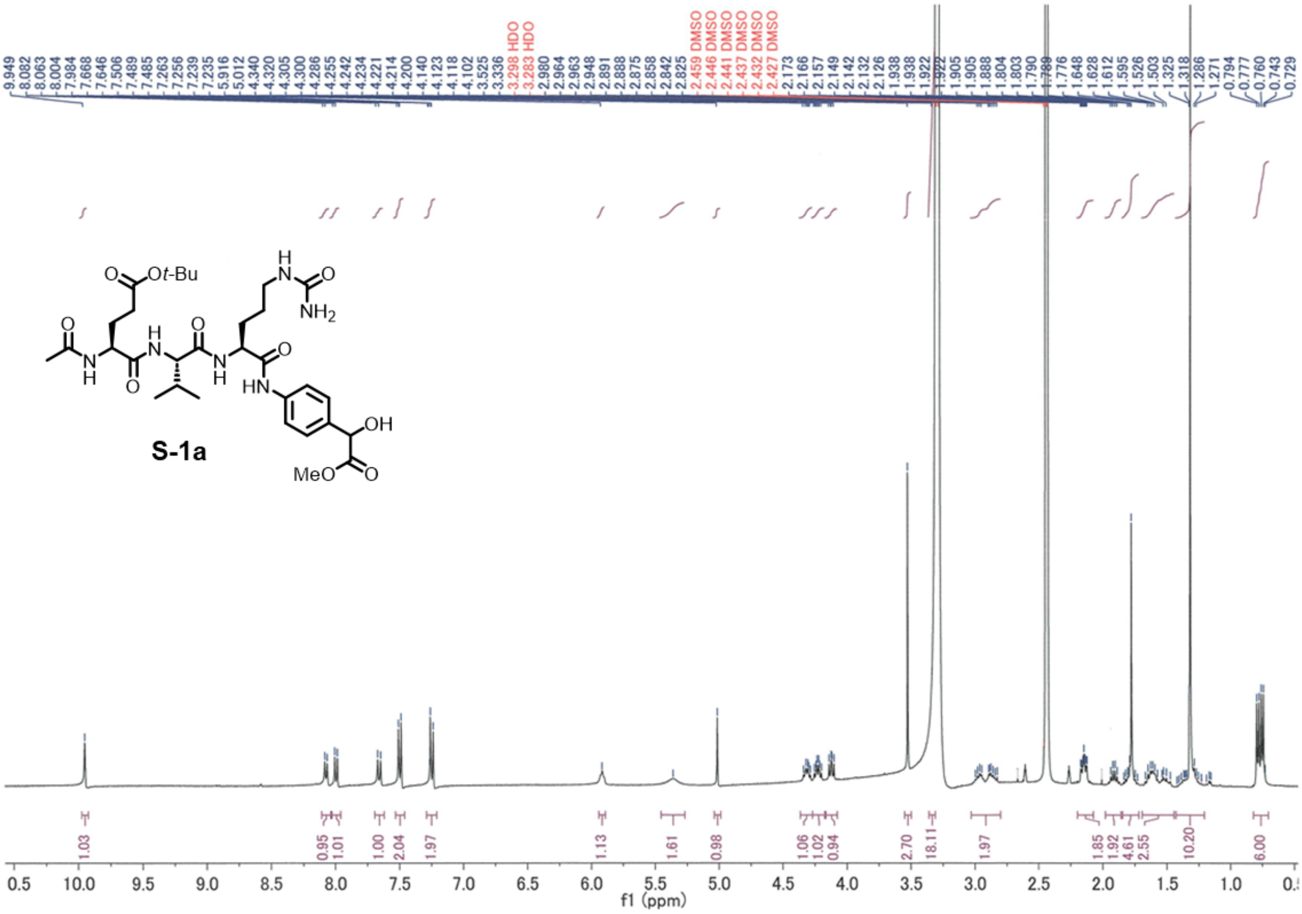

Figure S2. 1H NMR of compound **S-1a**

a)

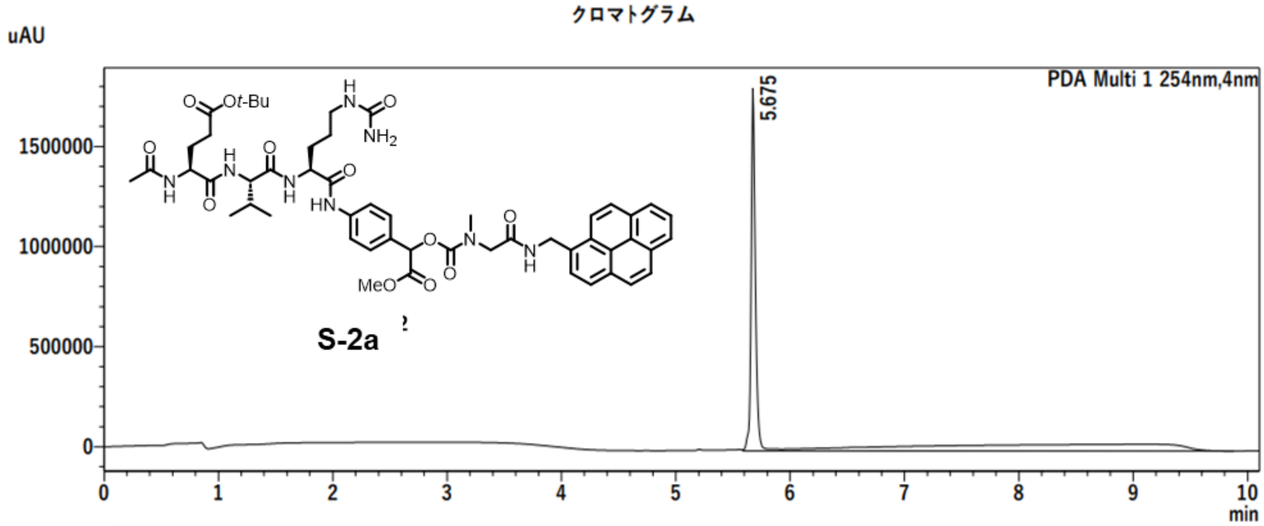

b)

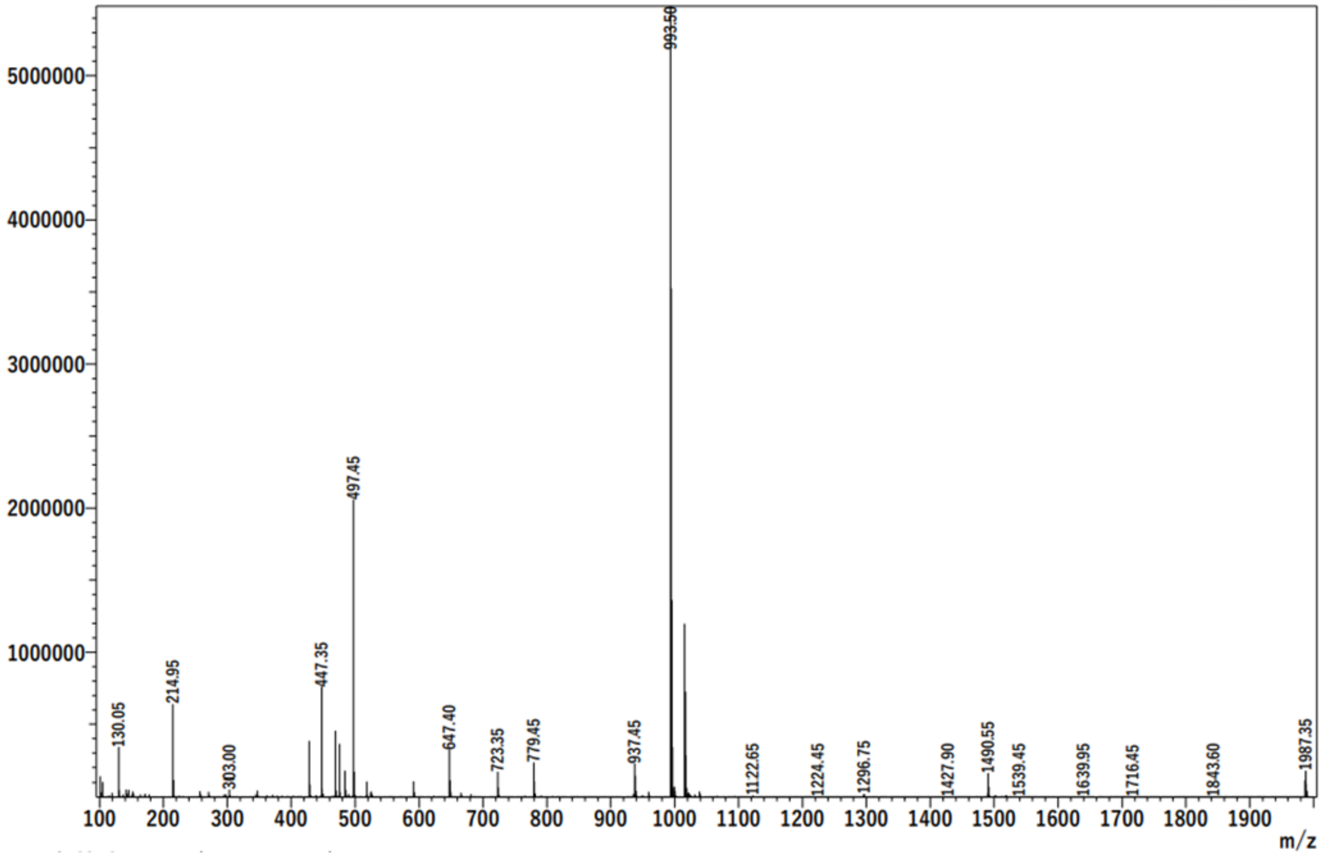

Figure S3. Analysis of compound **S-2a**, a) HPLC analysis, b) MS analysis

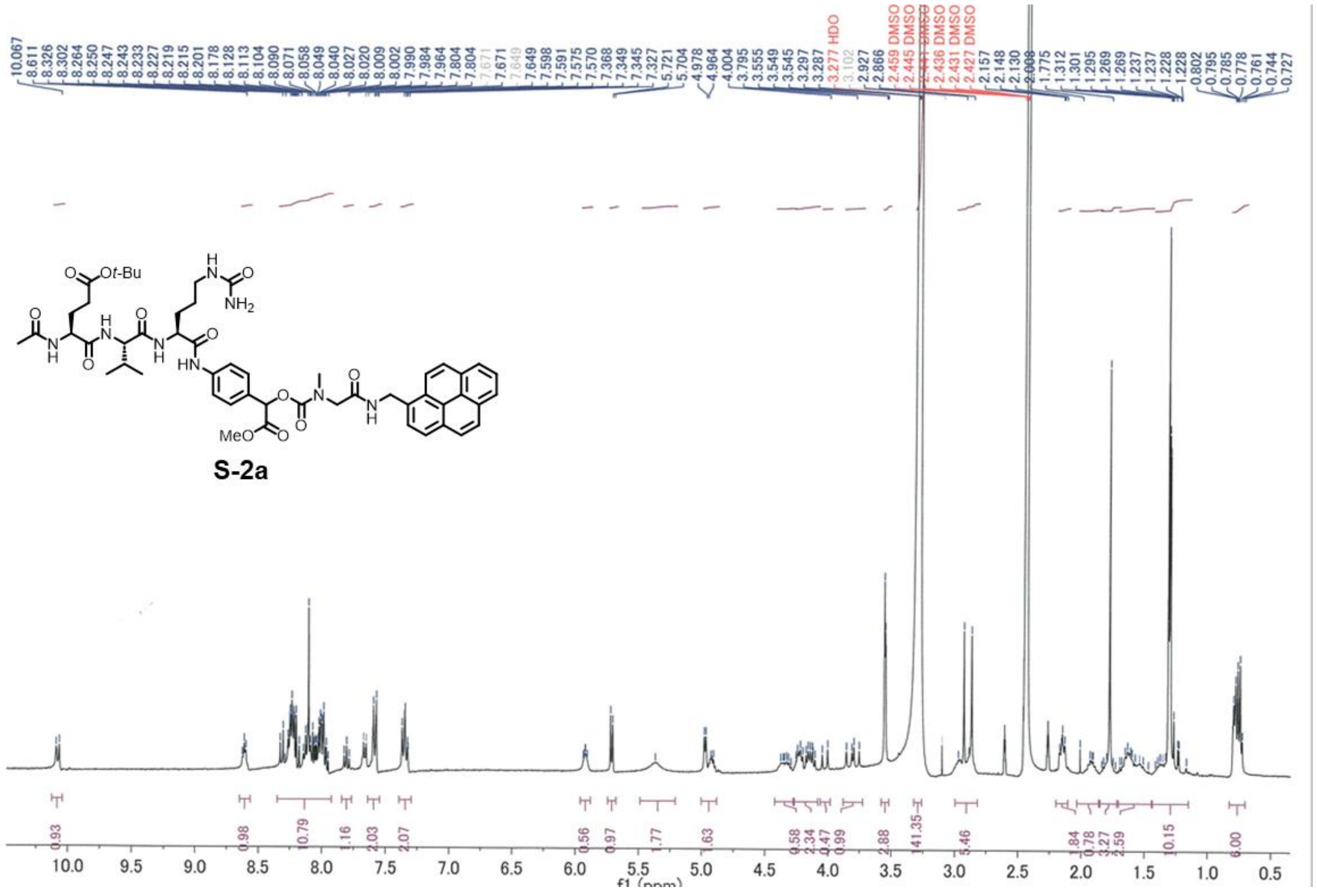

Figure S4. 1H NMR of compound **S-2a**

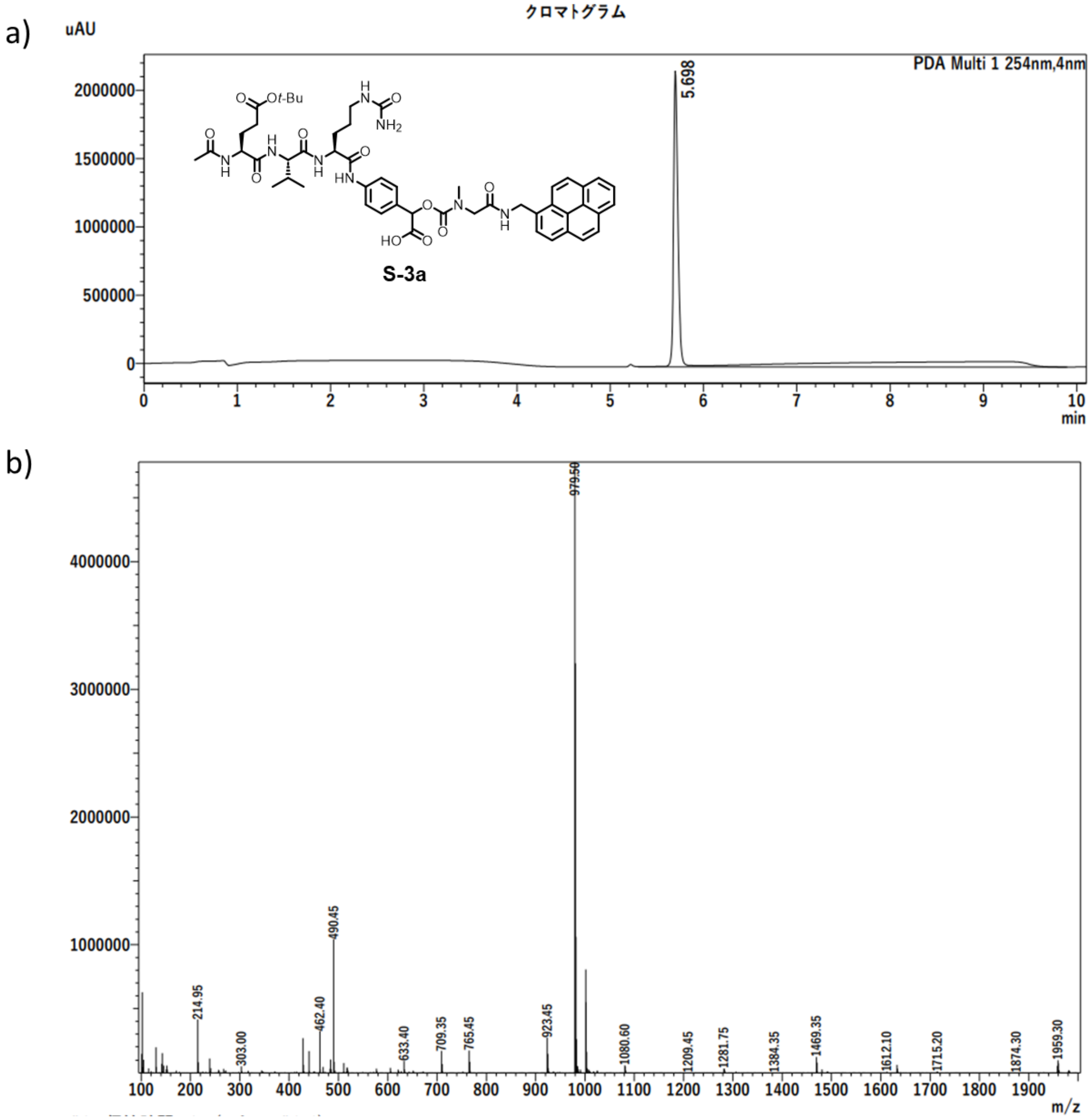

Figure S5. Analysis of compound **S-3a**, a) HPLC analysis, b) MS analysis

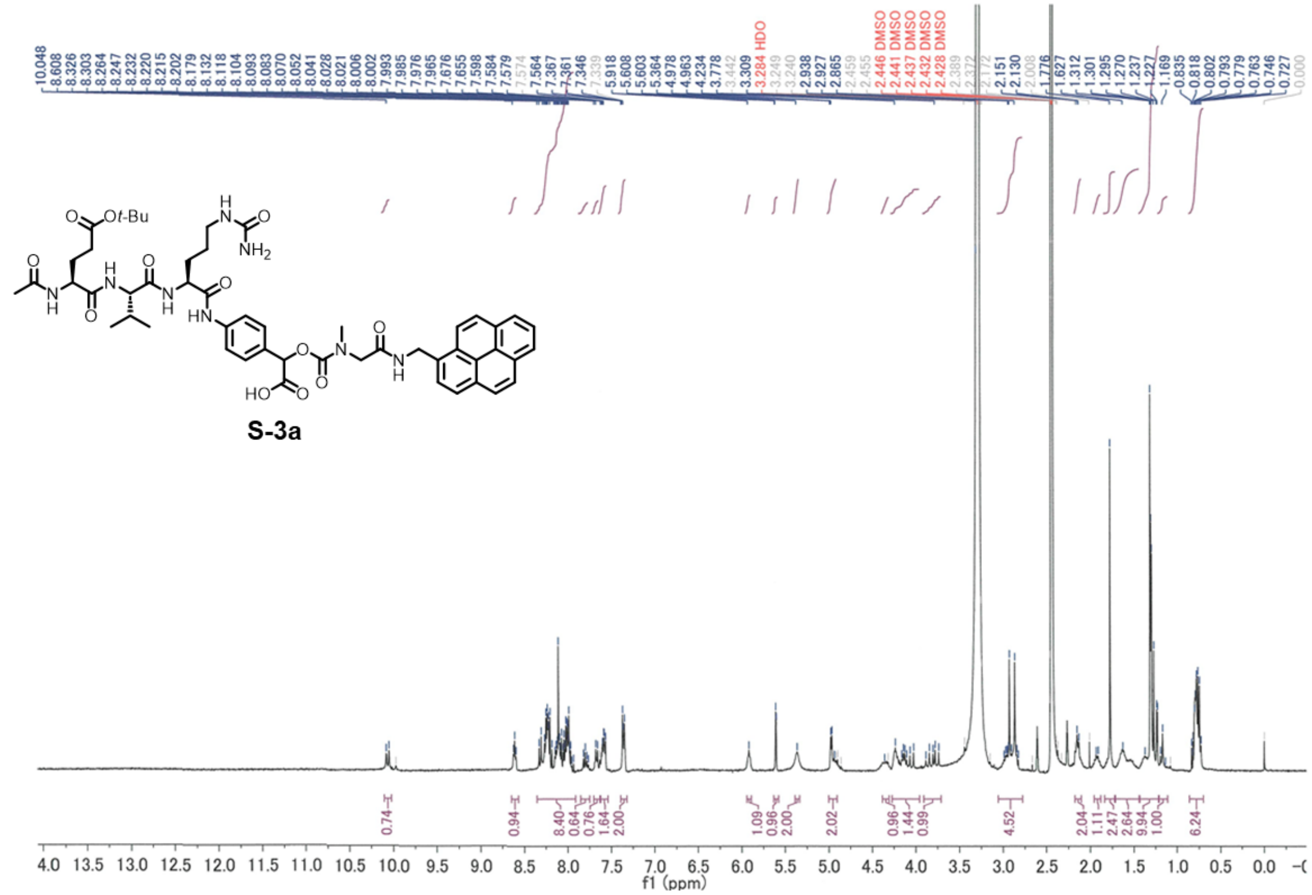

Figure S6. 1H NMR of compound **S-3a**

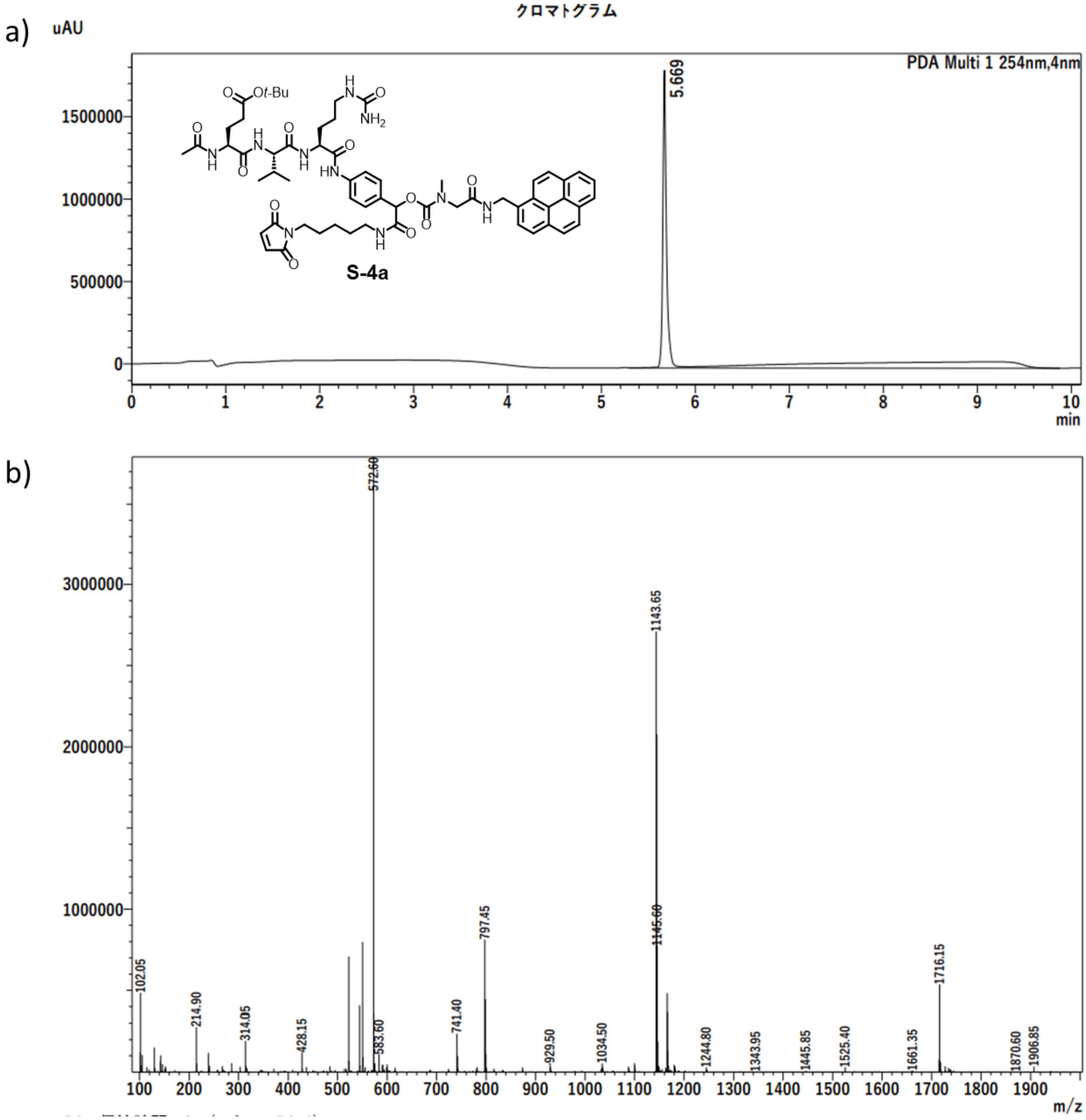

Figure S7. Analysis of compound **S-4a**, a) HPLC analysis, b) MS analysis

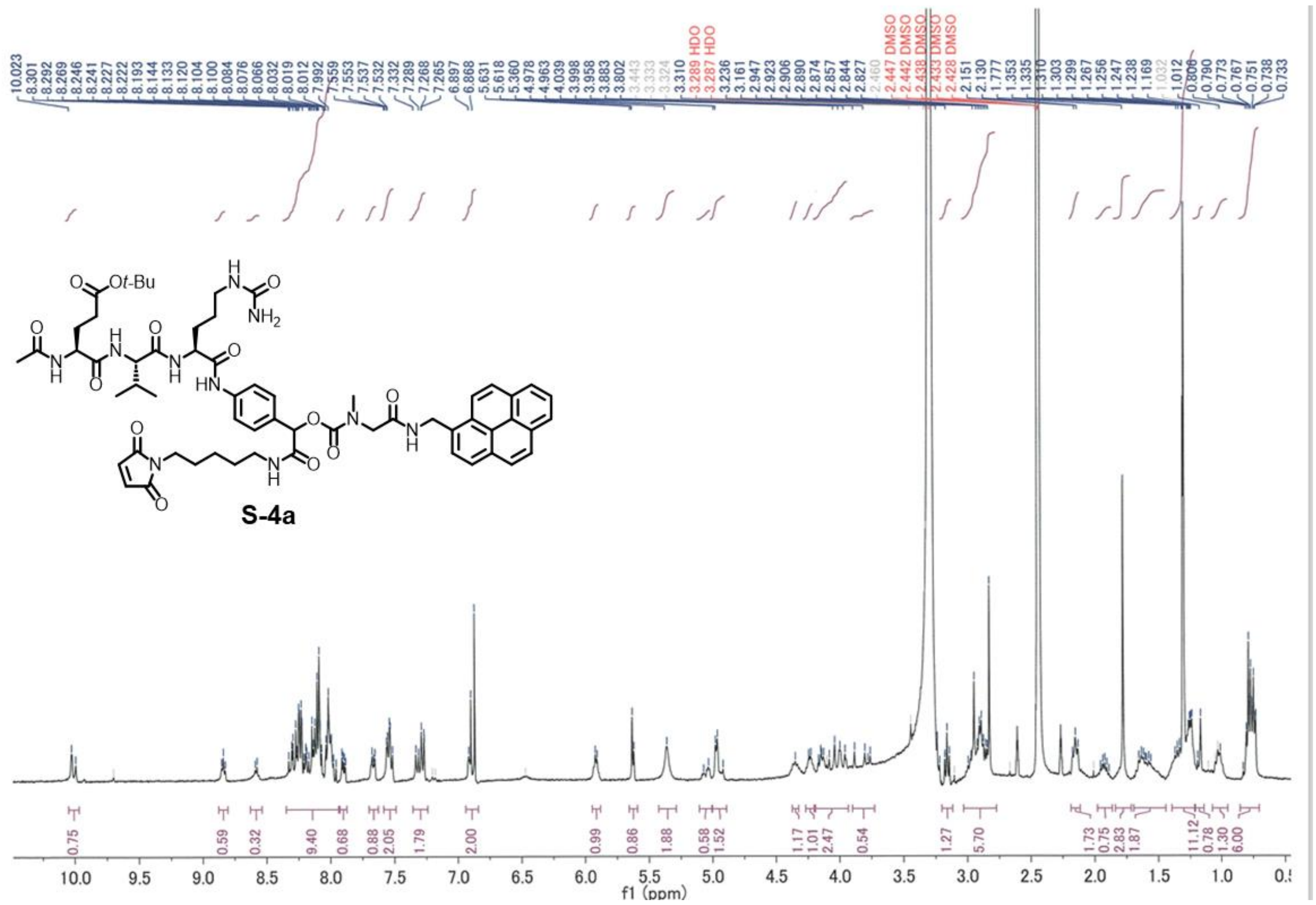

Figure S8. 1H NMR of compound **S-4a**

a)

b)

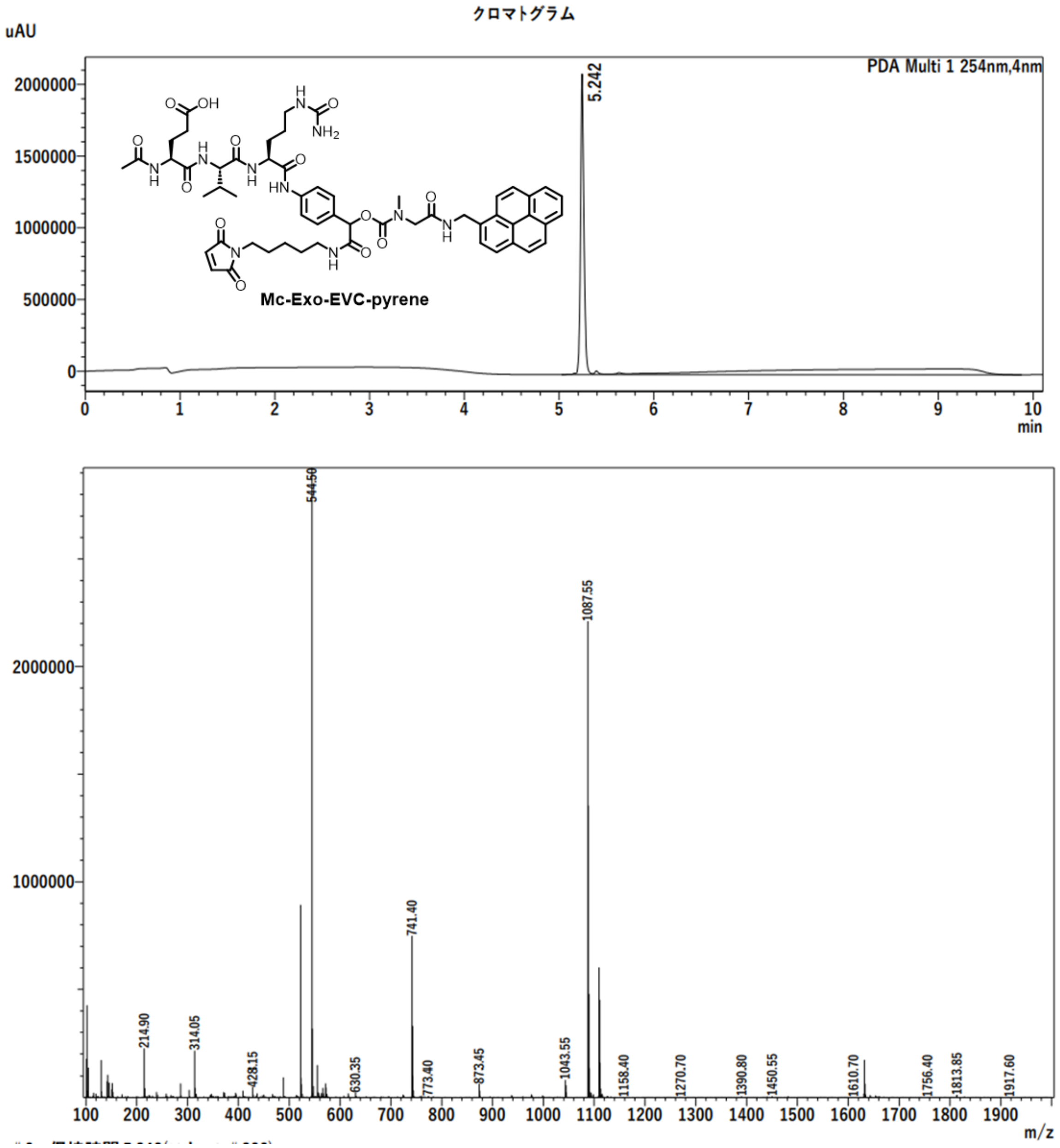

Figure S9. Analysis of compound Mc-Exo-EVC-pyrene, a) HPLC analysis, b) MS analysis

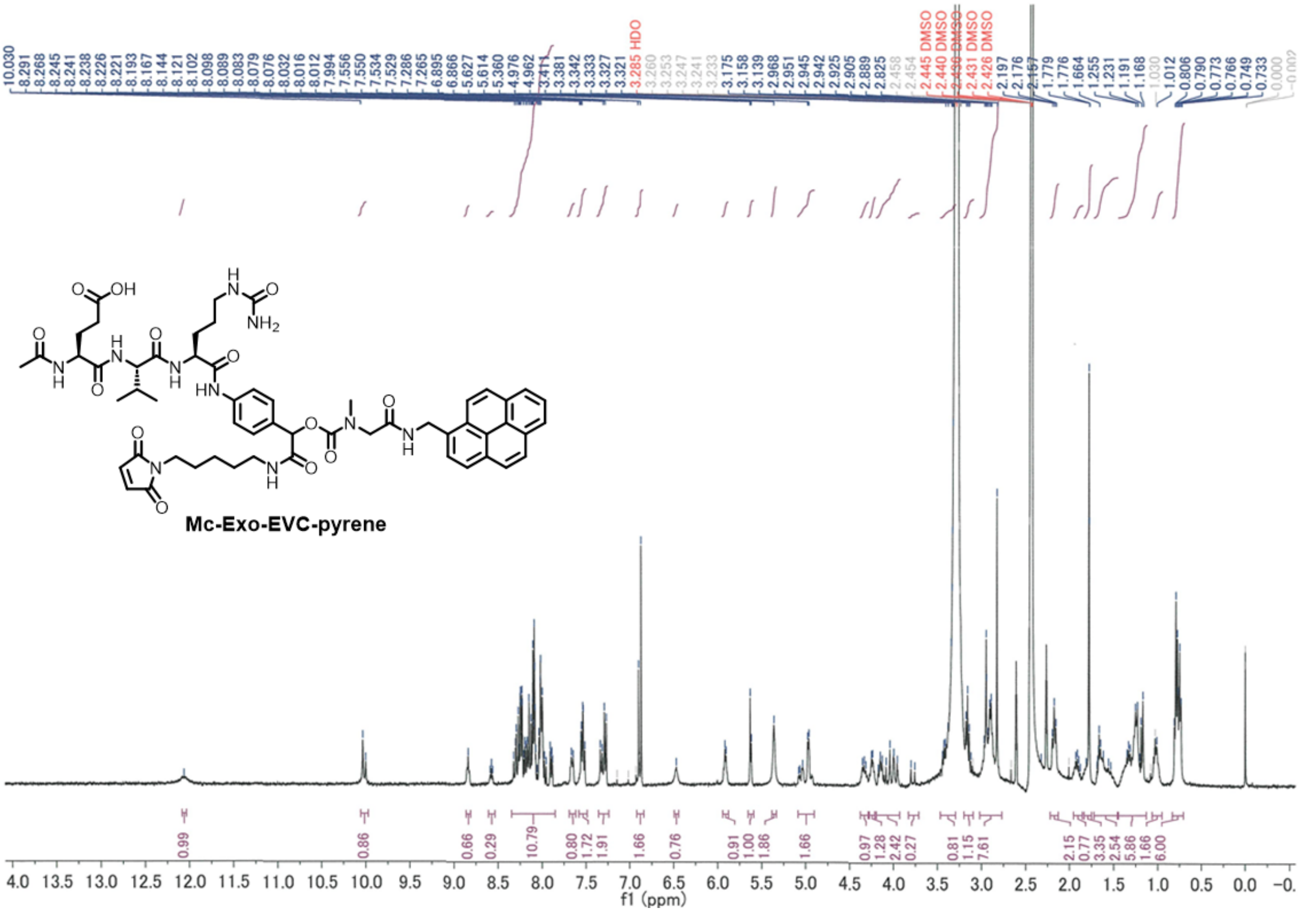

Figure S10. 1H NMR of Mc-Exo-EVC-pyrene

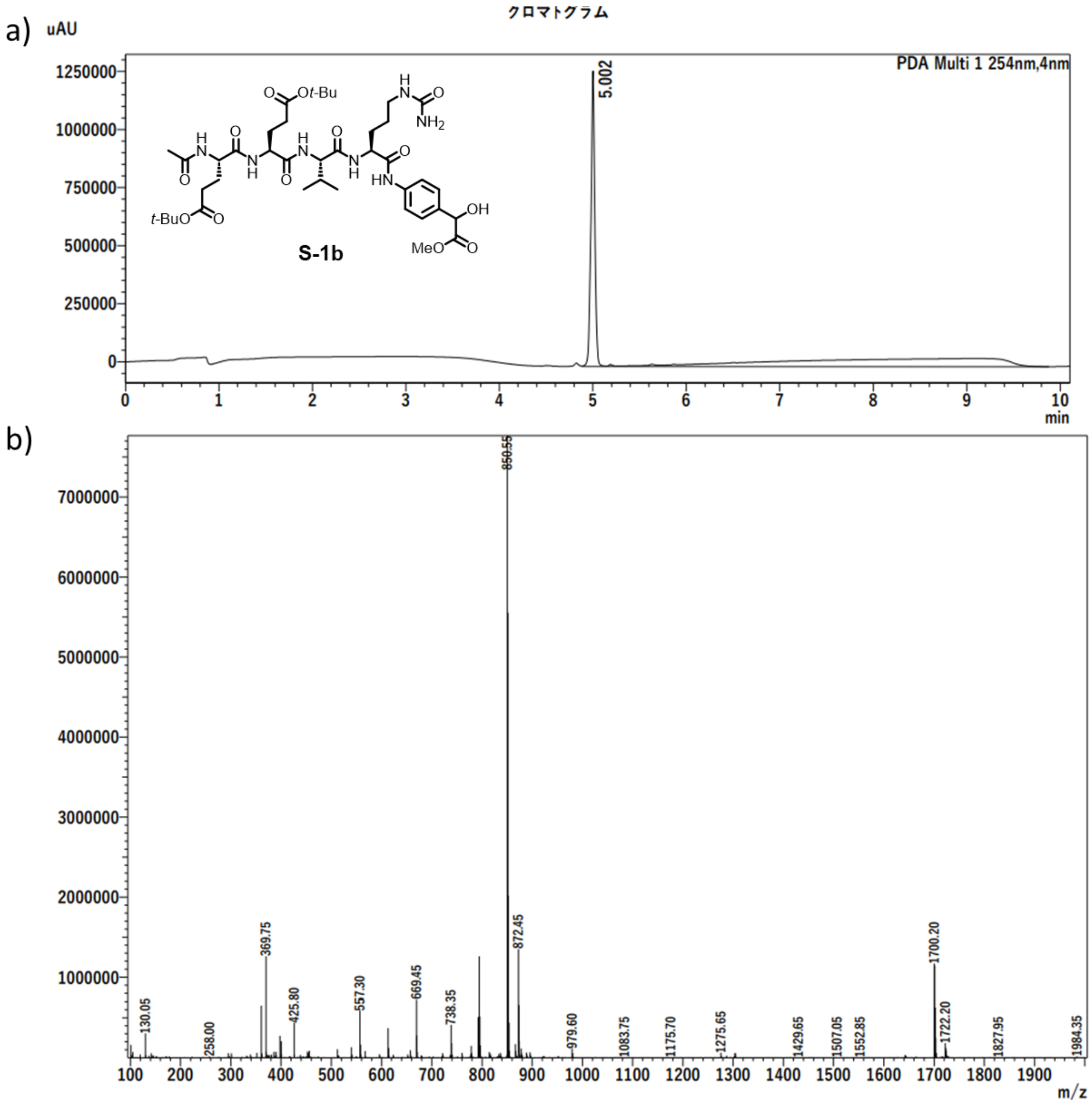

Figure S11. Analysis of compound **S-1b**, a) HPLC analysis, b) MS analysis

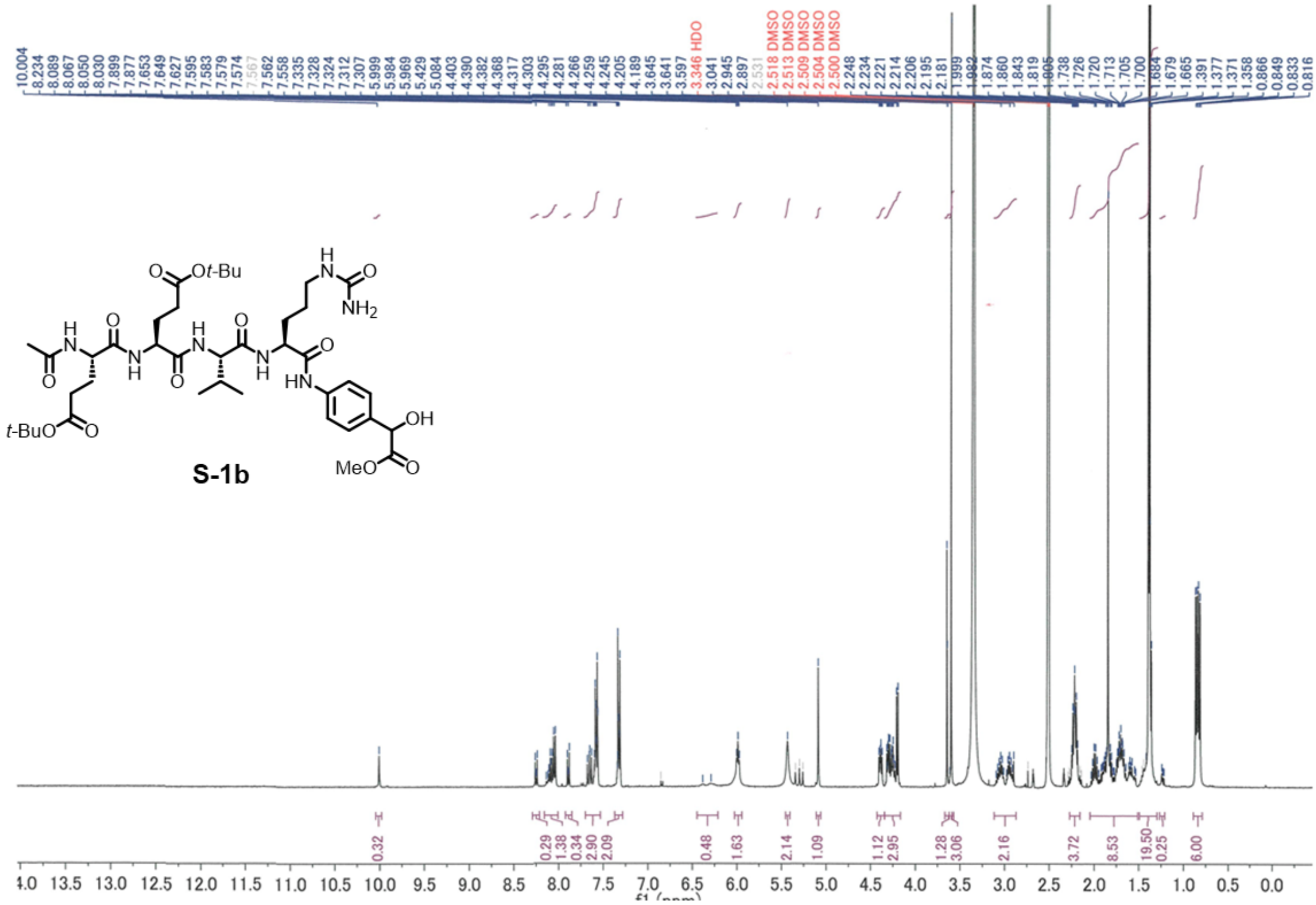

Figure S12. 1H NMR of compound **S-1b**

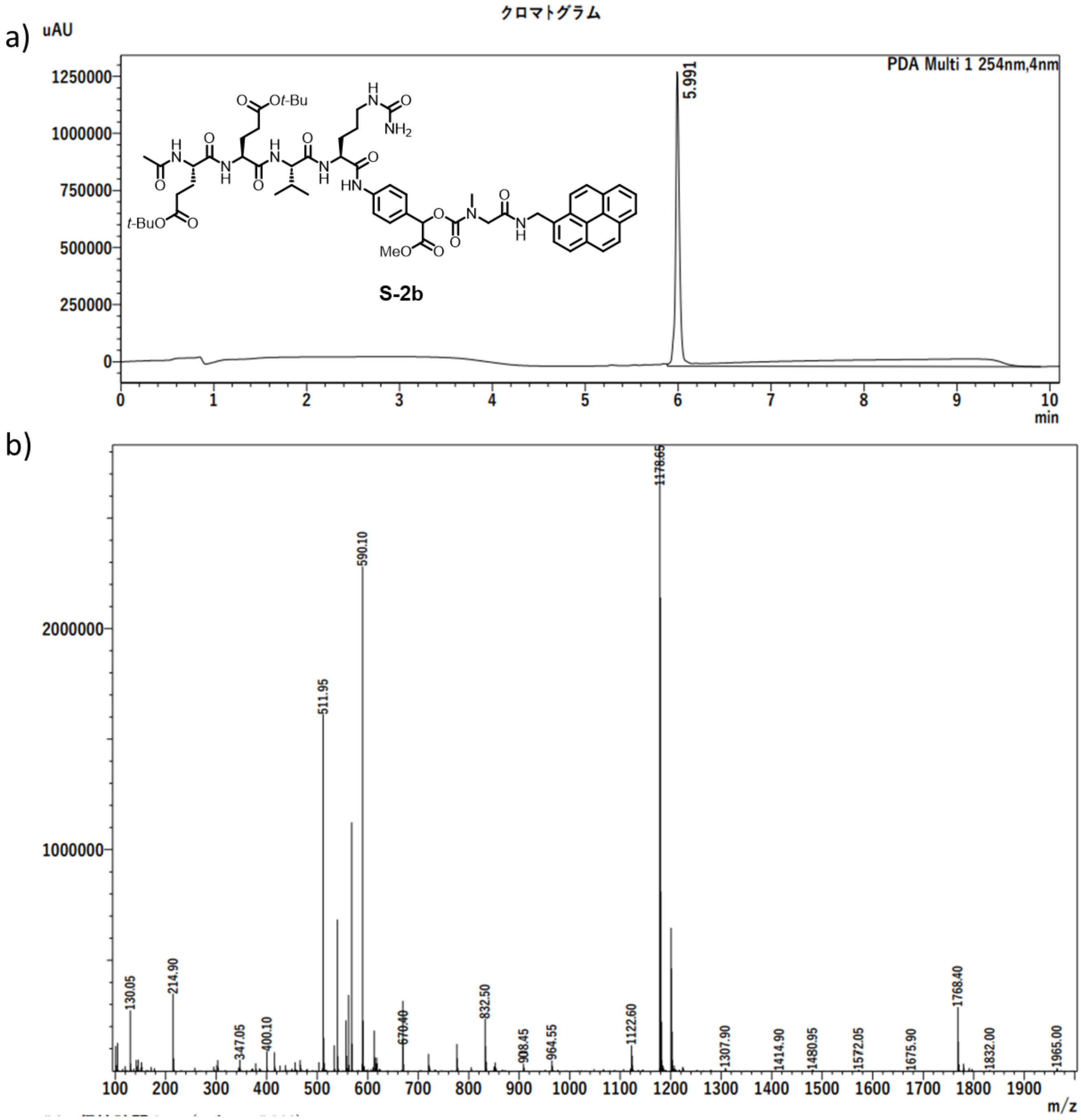

Figure S13. Analysis of compound **S-2b**, a) HPLC analysis, b) MS analysis

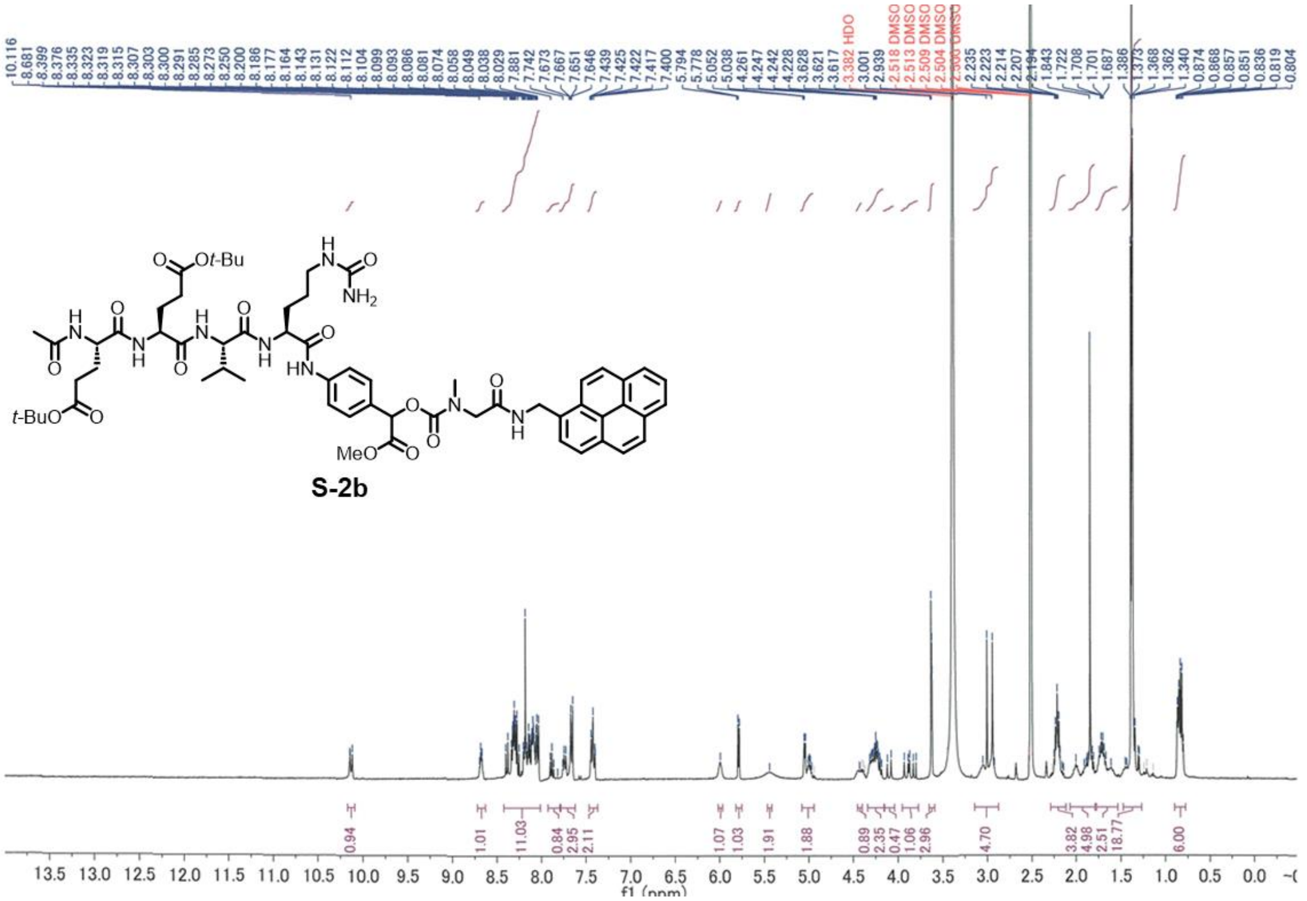

Figure S14. 1H NMR of compound **S-2b**

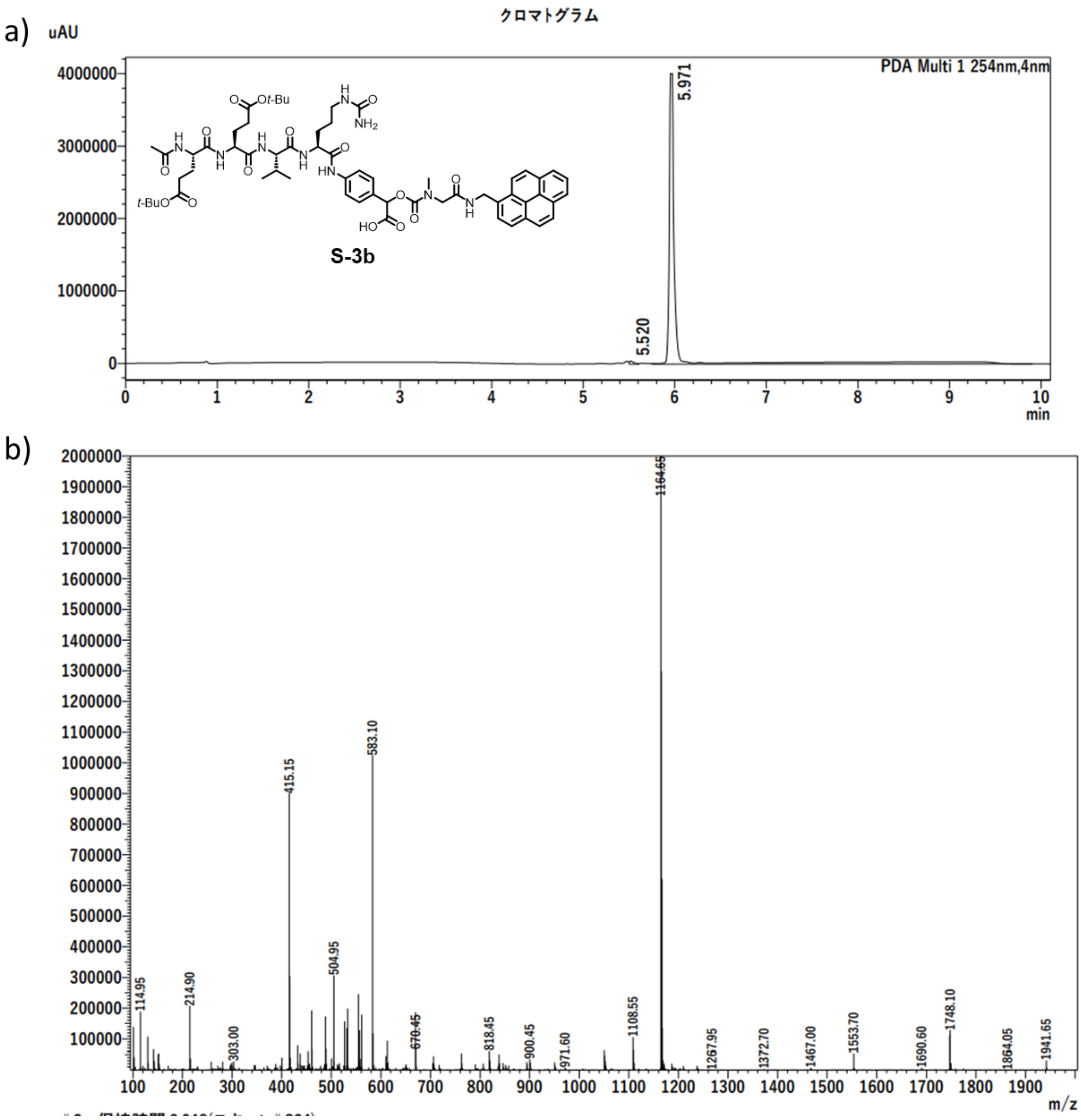

Figure S15. Analysis of compound **S-3b**, a) HPLC analysis, b) MS analysis

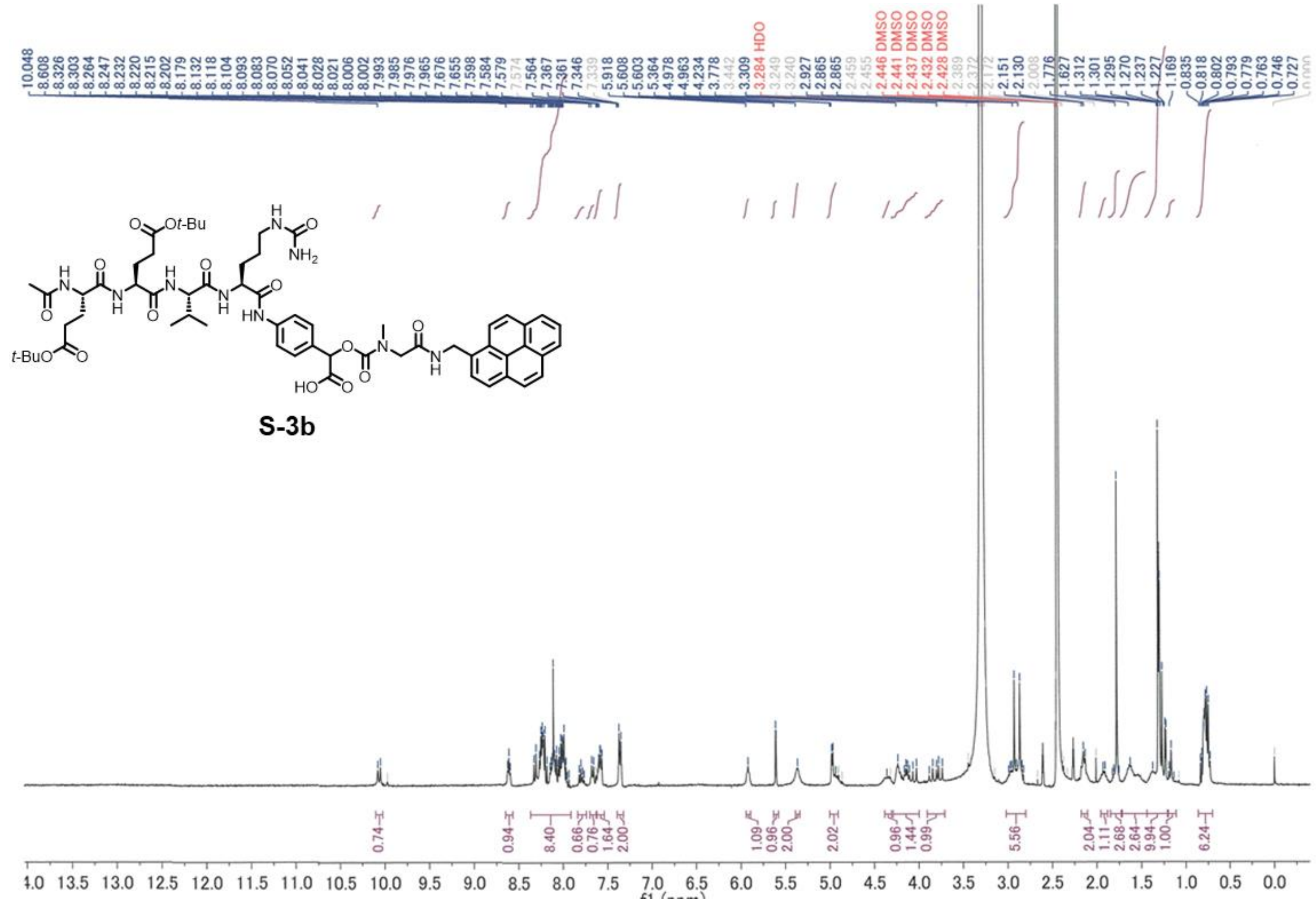

Figure S16. 1H NMR of compound **S-3b**

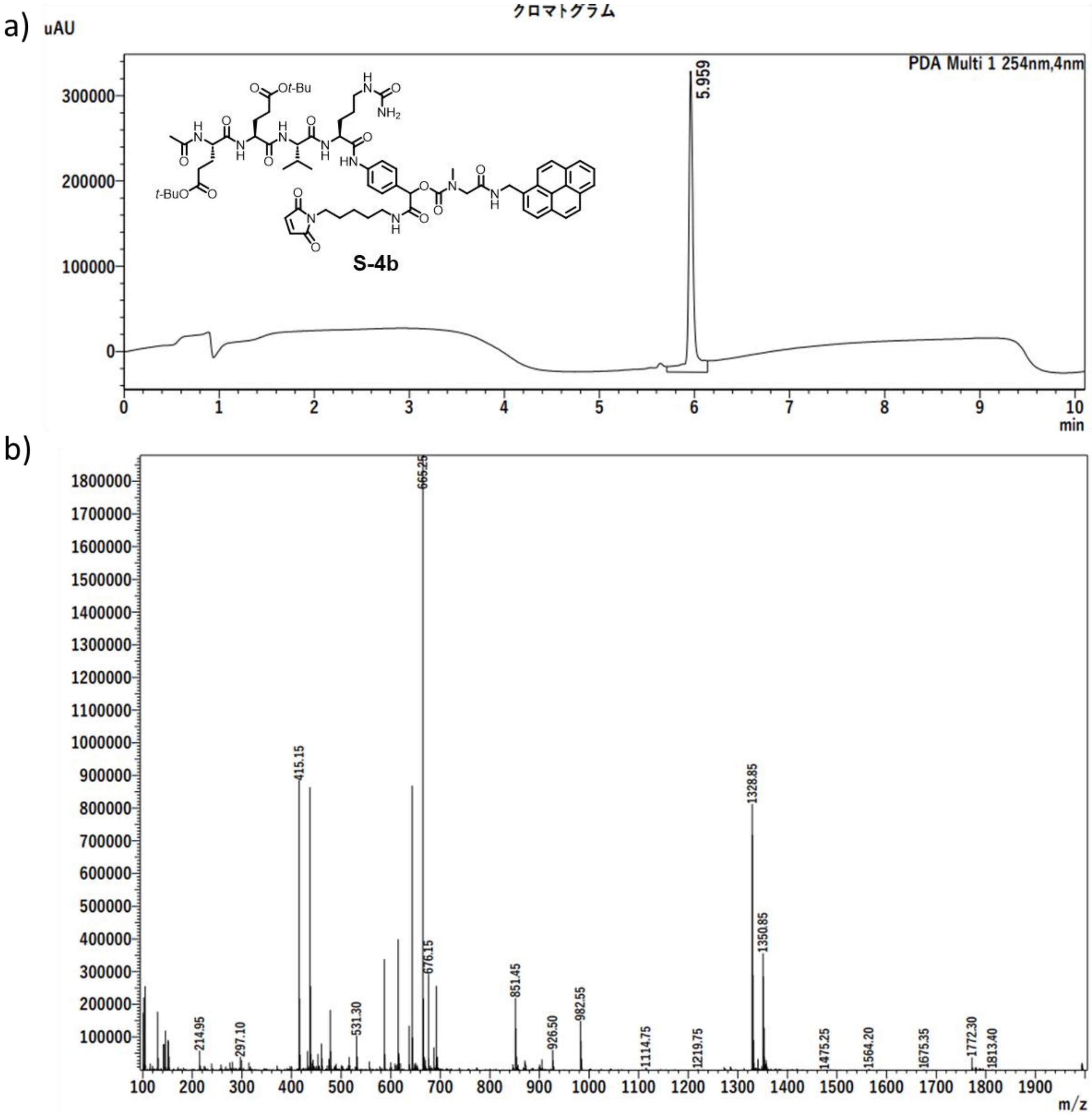

Figure S17. Analysis of compound **S-4b**, a) HPLC analysis, b) MS analysis

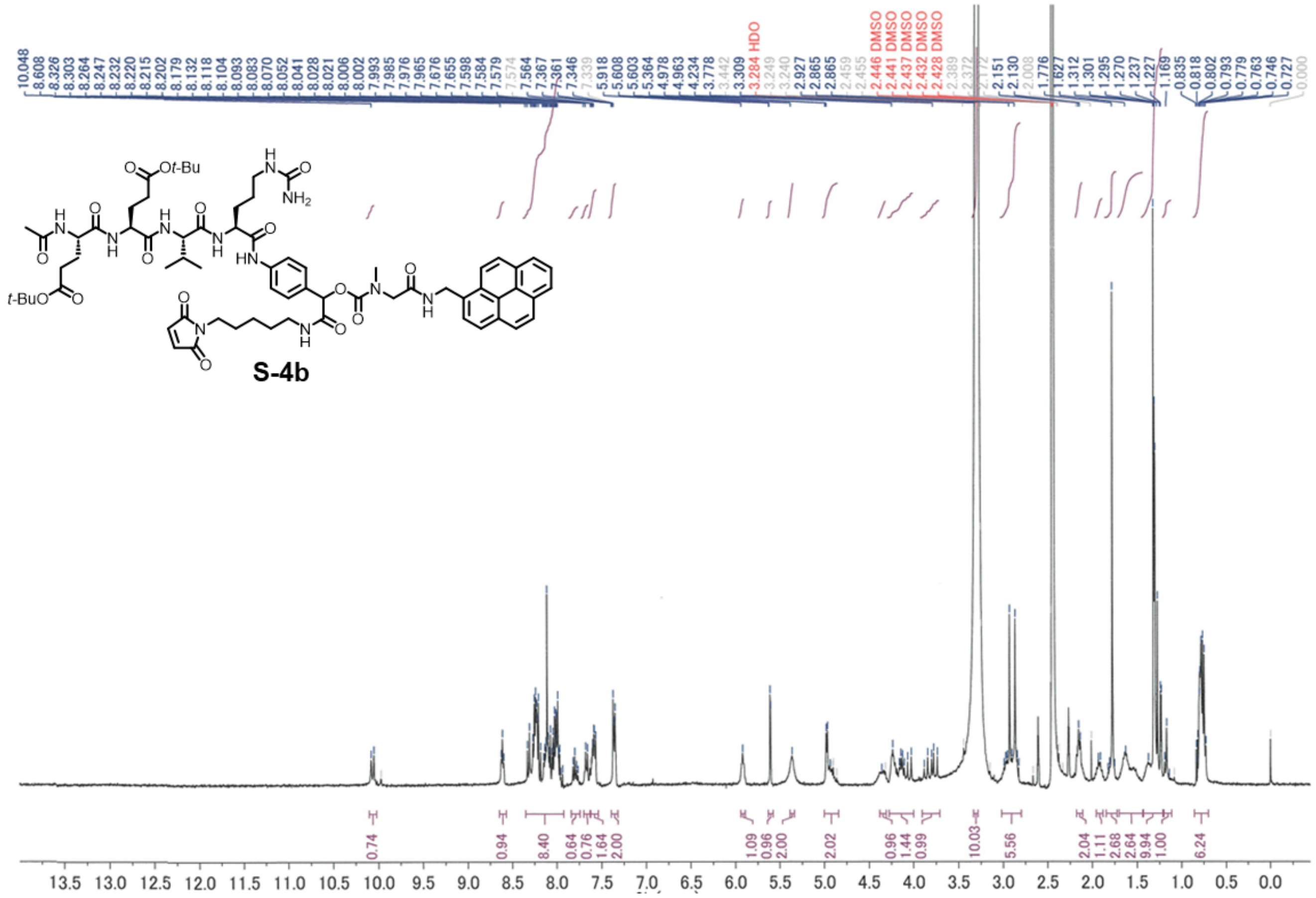

Figure S18. 1H NMR of compound **S-4b**

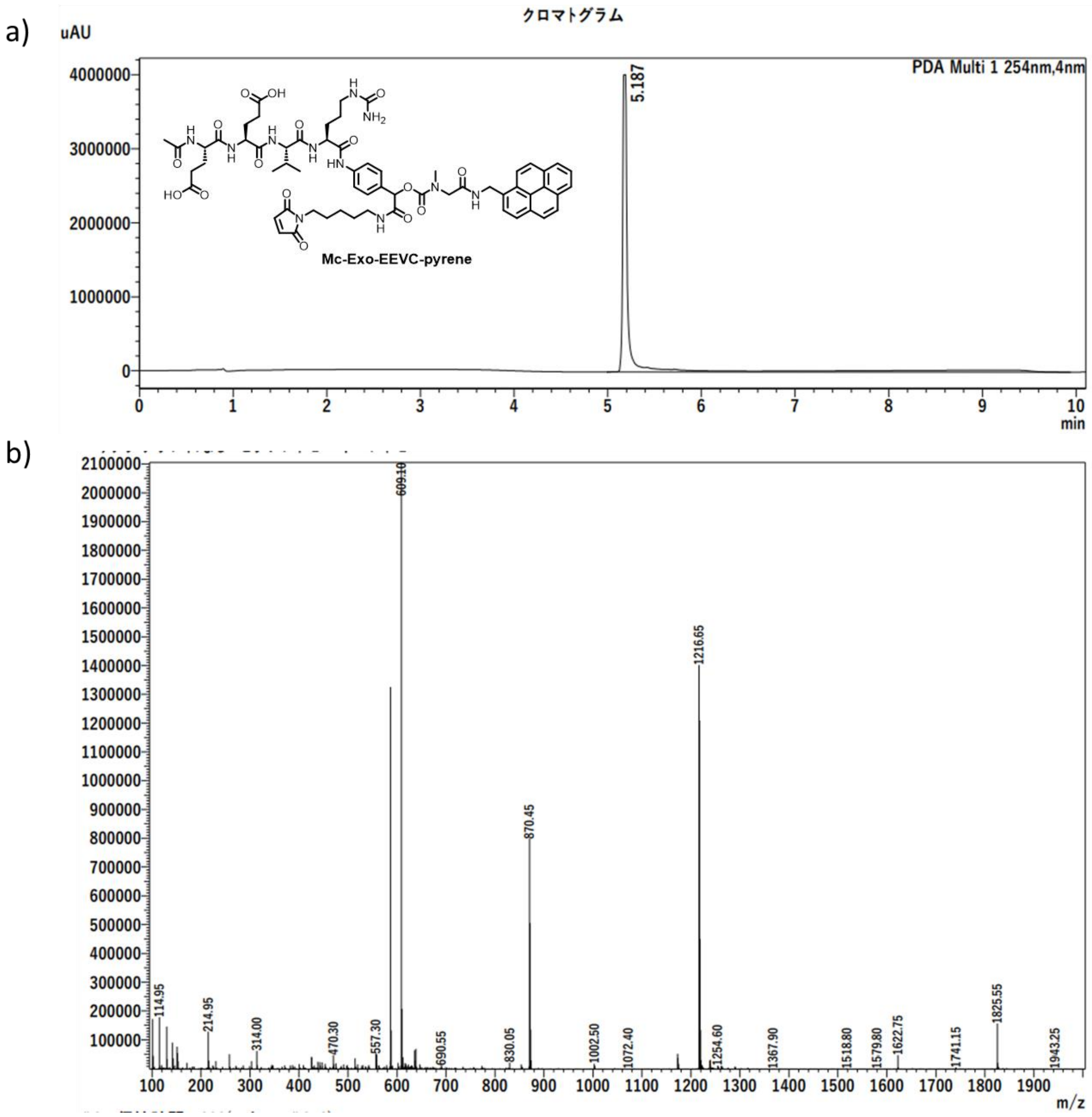

Figure S19. Analysis of compound Mc-Exo-EEVC-pyrene, a) HPLC analysis, b) MS analysis

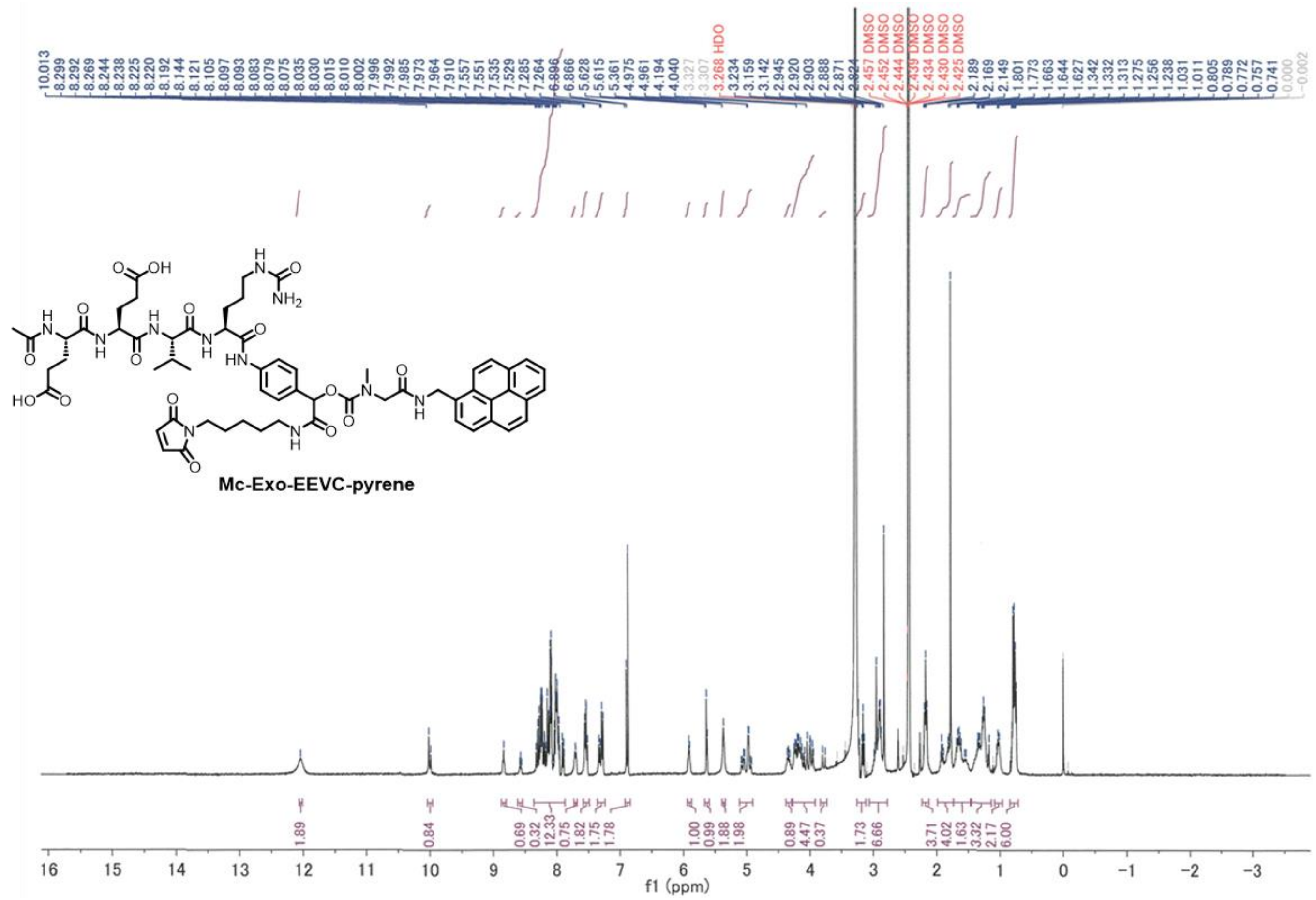

Figure S20. 1H NMR of compound **S-8**

a)

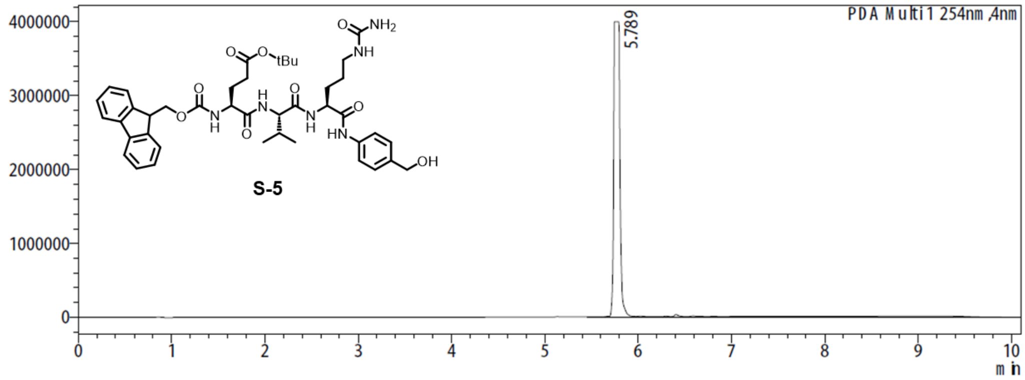

b)

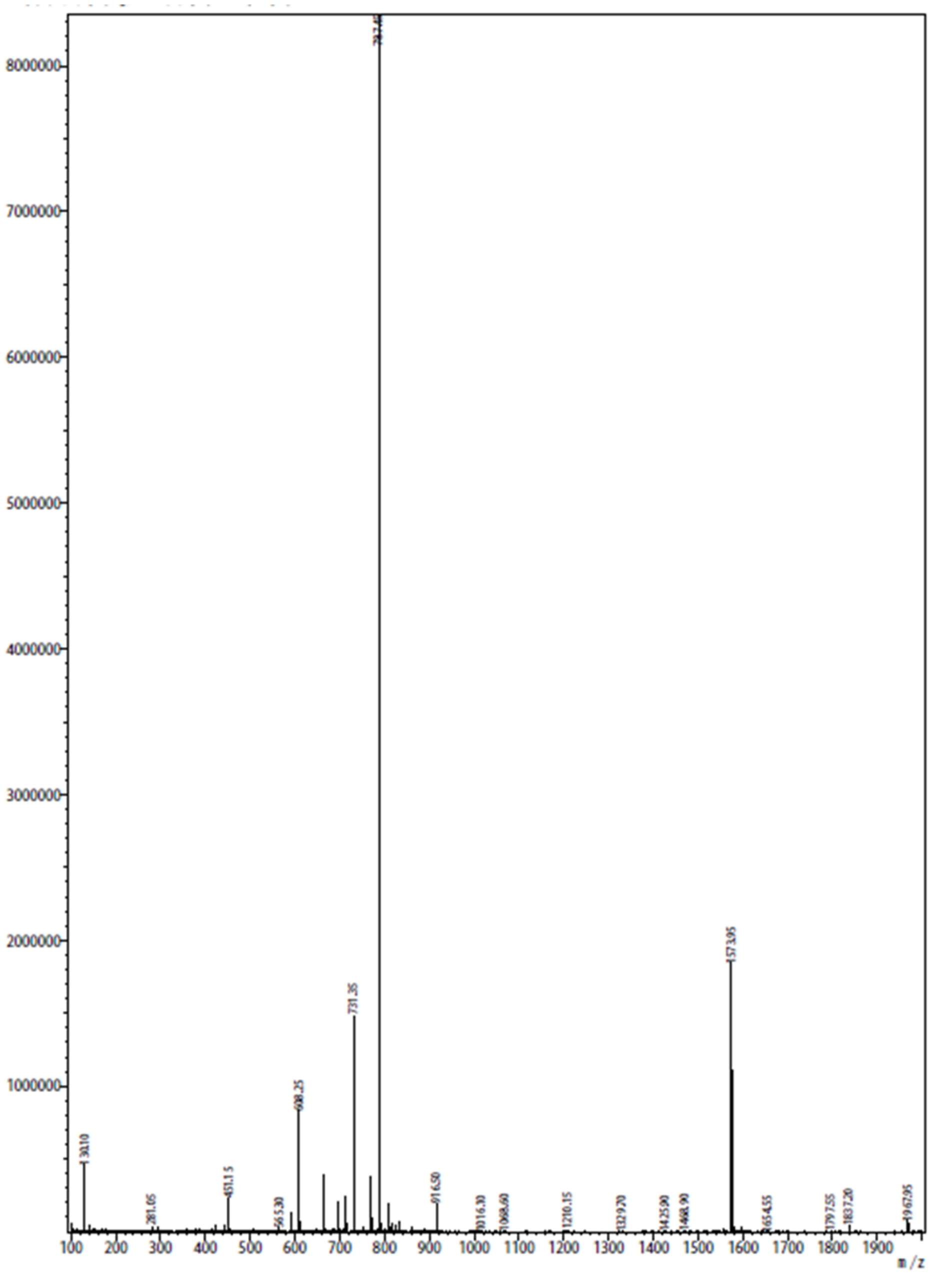

Figure S21. Analysis of compound **S-5**, a) HPLC analysis, b) MS analysis

a)

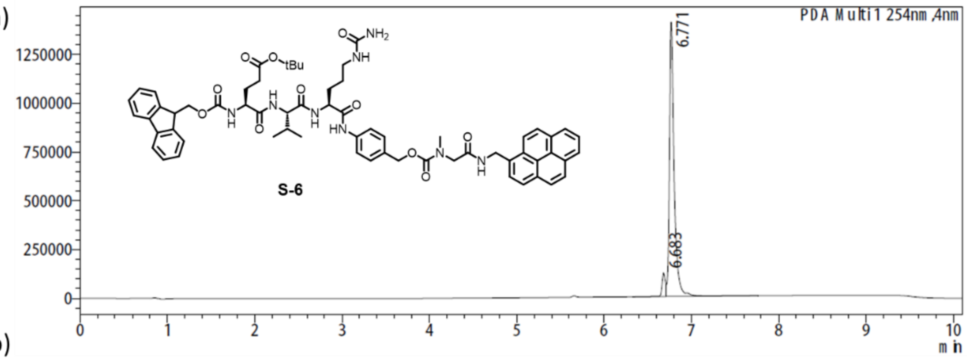

b)

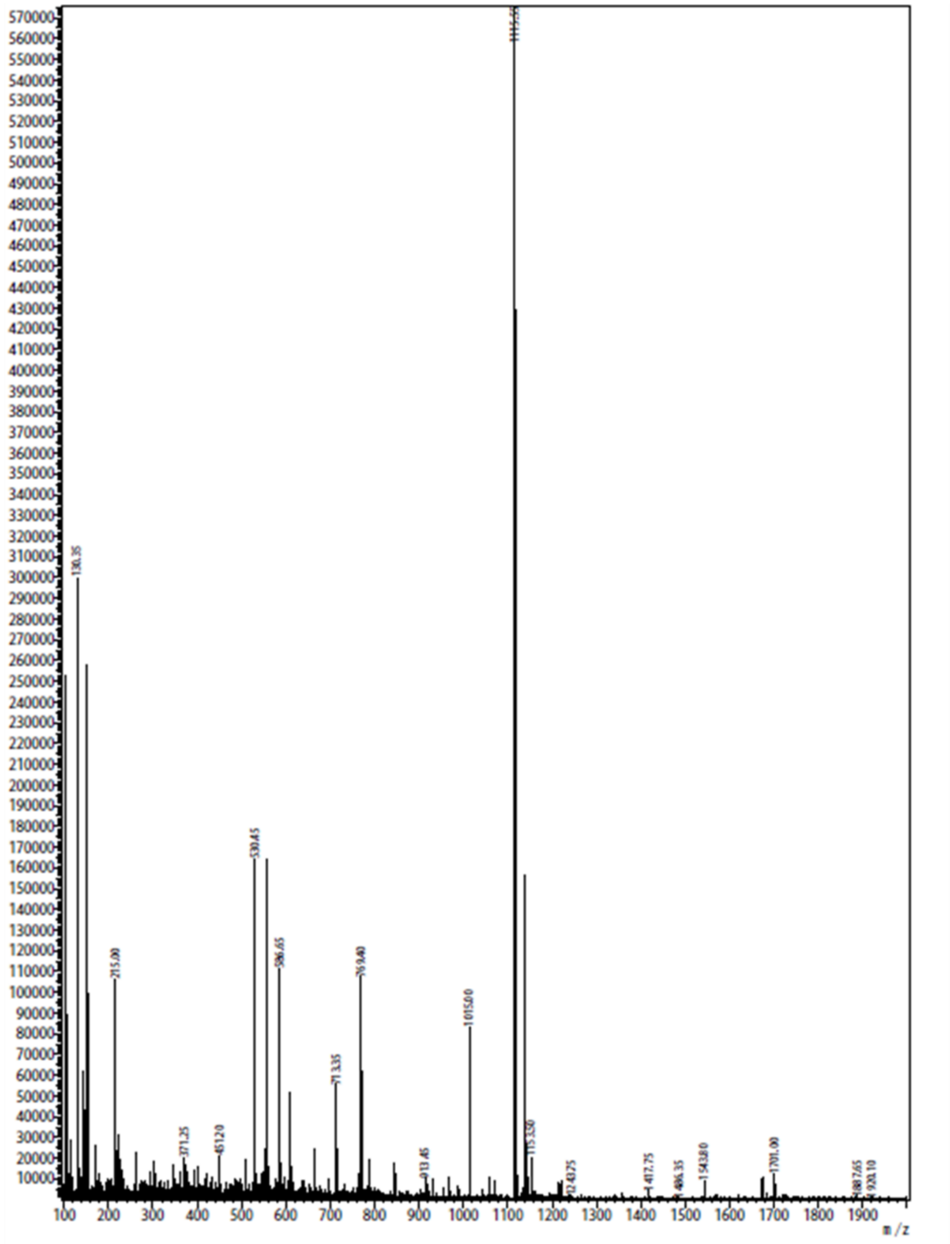

Figure S22. Analysis of compound **S-6**, a) HPLC analysis, b) MS analysis

a)

b)

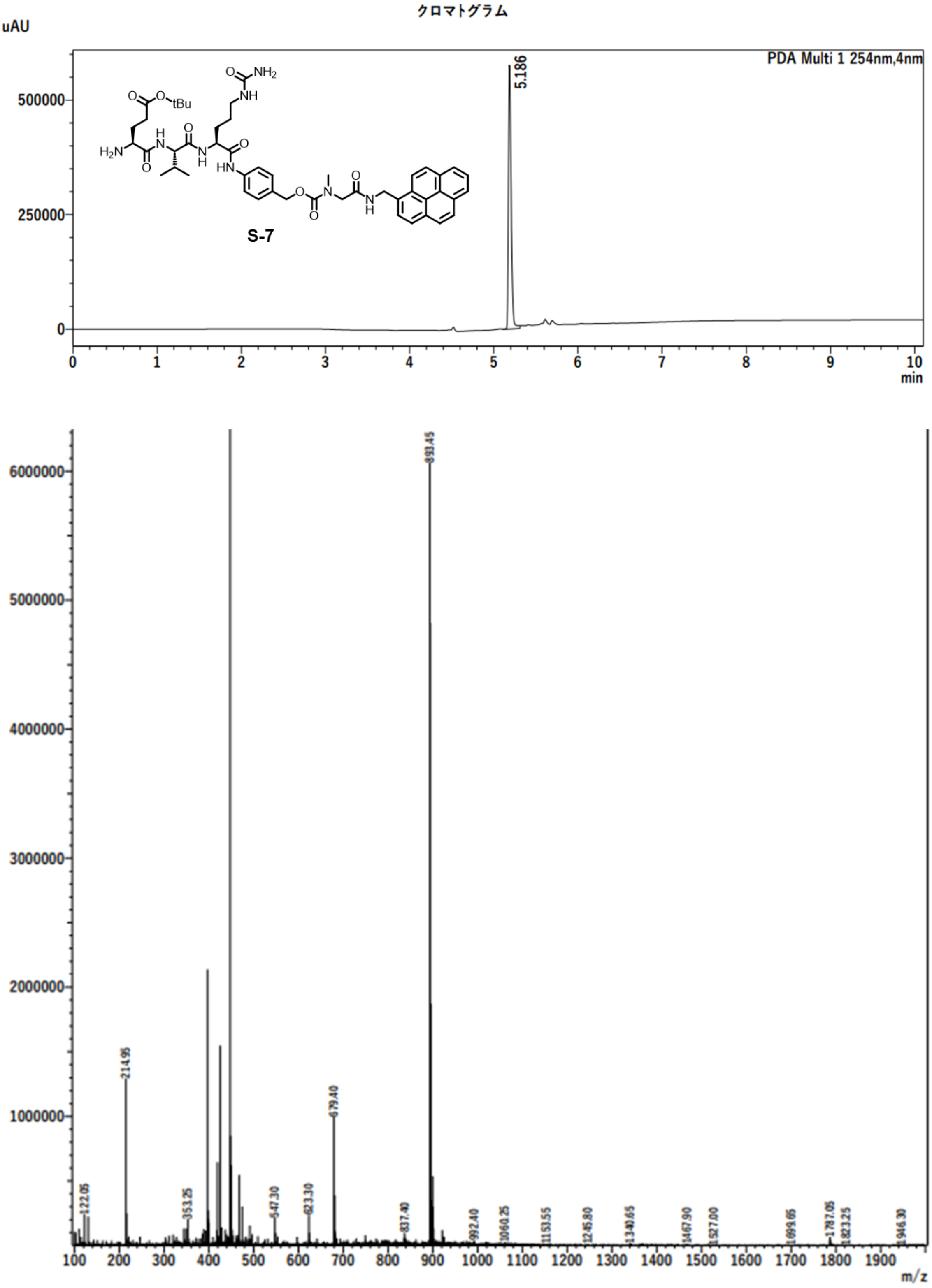

Figure S23. Analysis of compound **S-7**, a) HPLC analysis, b) MS analysis

a)

b)

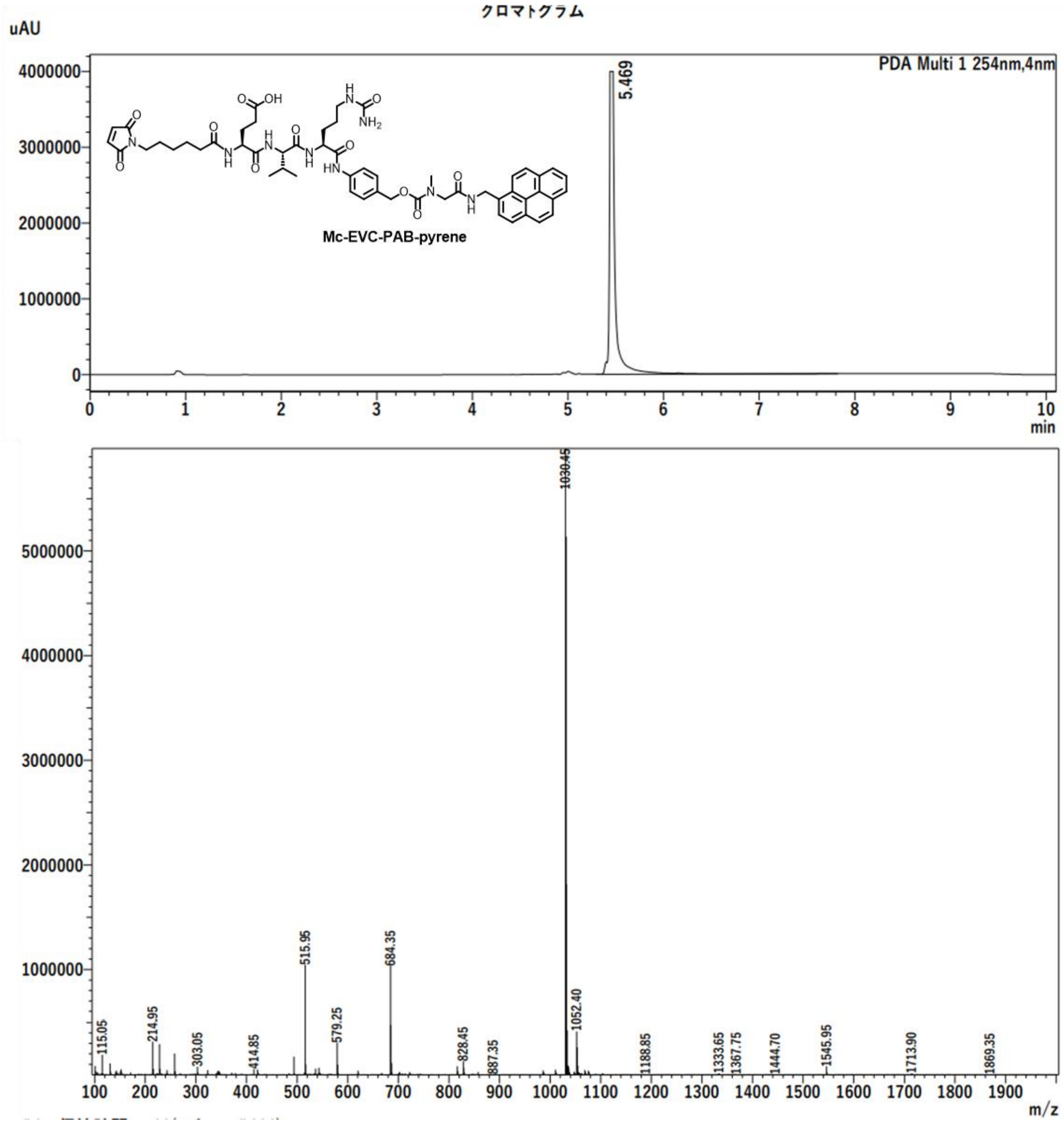

Figure S24. Analysis of compound Mc-EVC-PAB-pyrene, a) HPLC analysis, b) MS analysis

a)

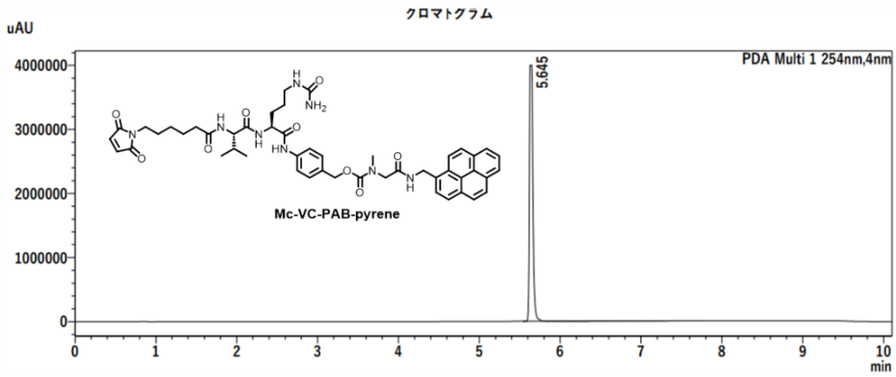

b)

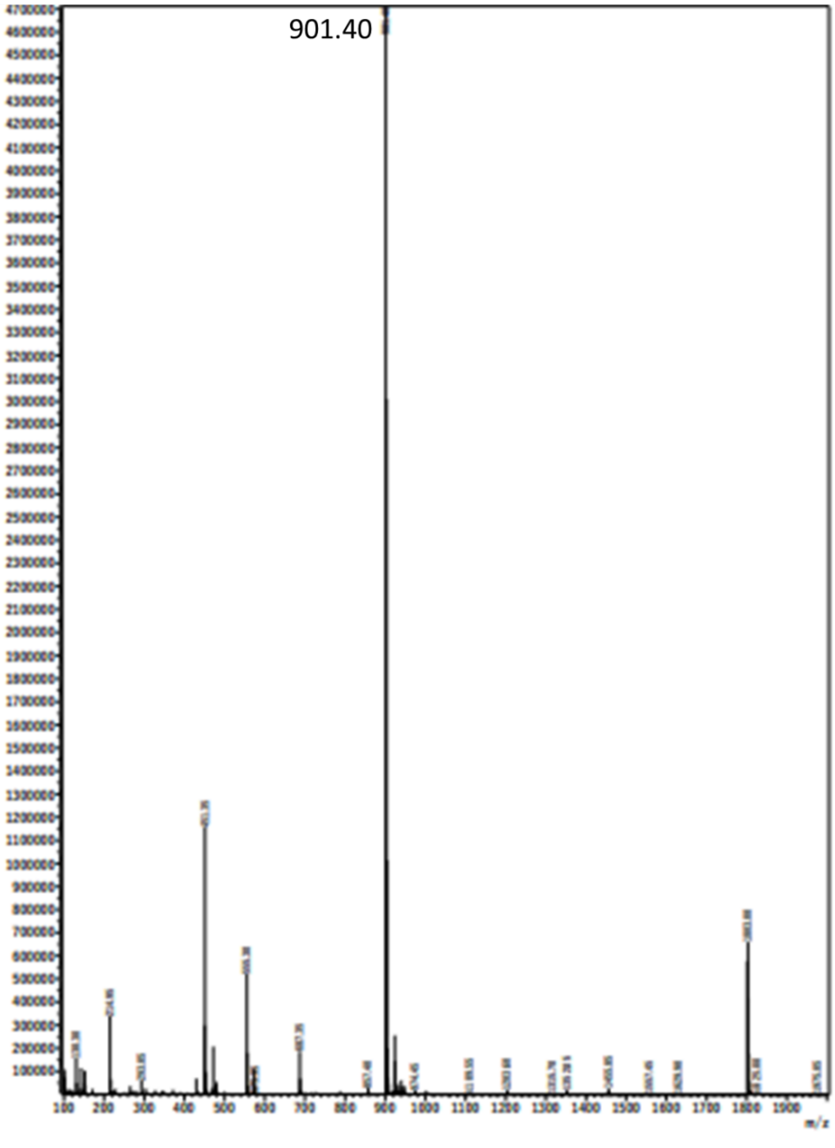

Figure S25. Analysis of compound Mc-VC-PAB-pyrene, a) HPLC analysis, b) MS analysis

a)

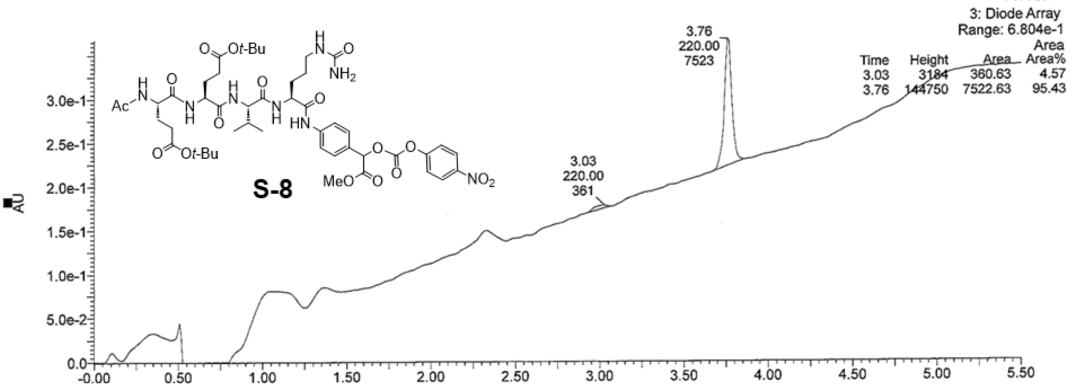

b)

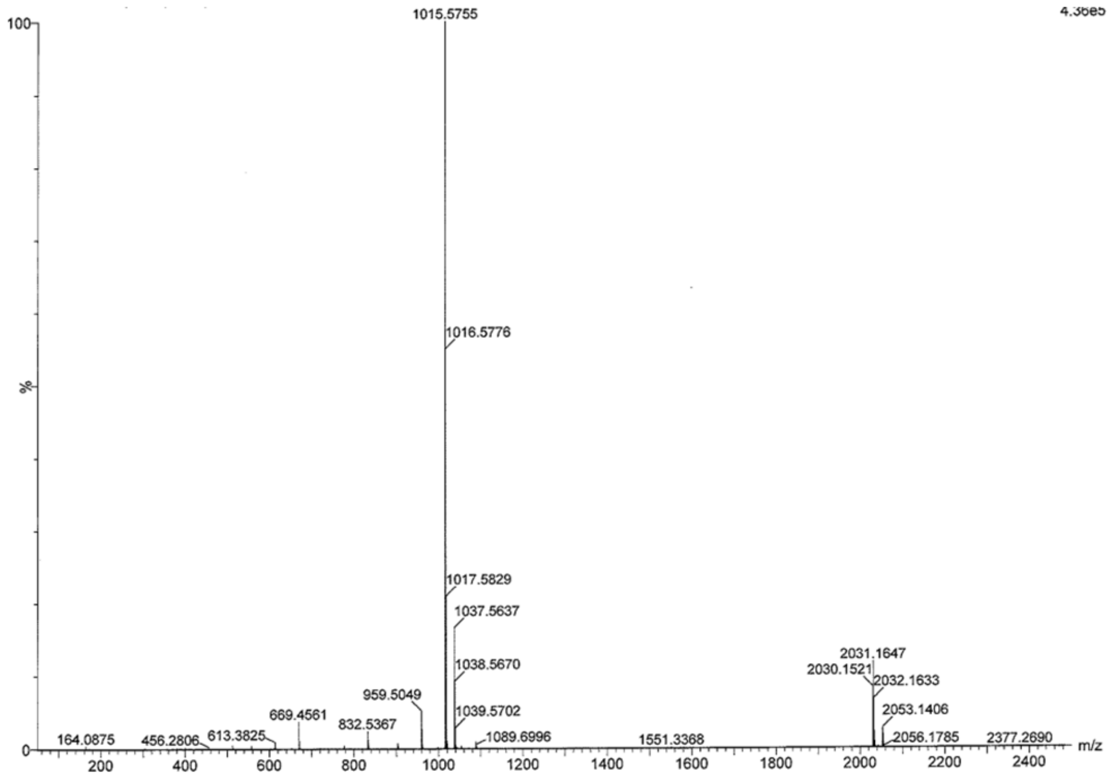

Figure S26. Analysis of compound **S-8**, a) HPLC analysis, b) MS analysis

a)

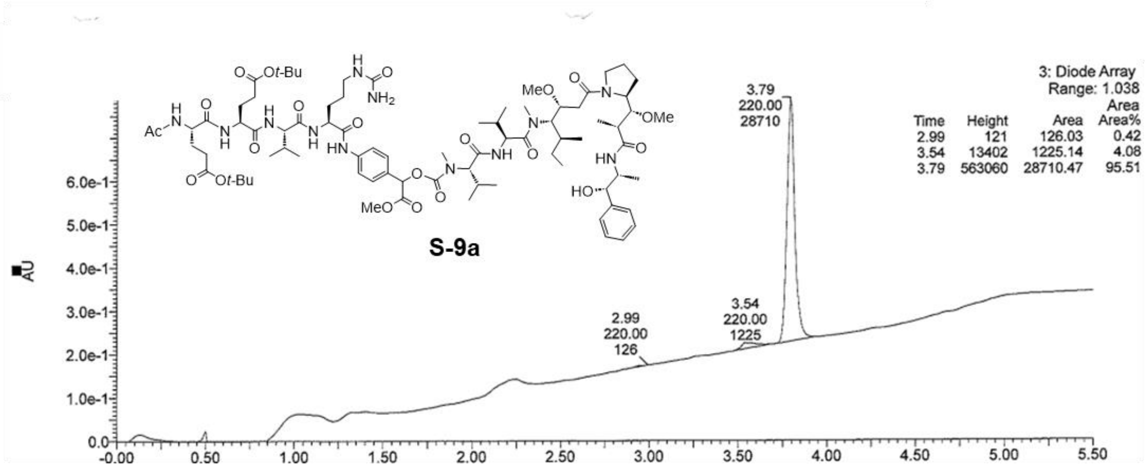

b)

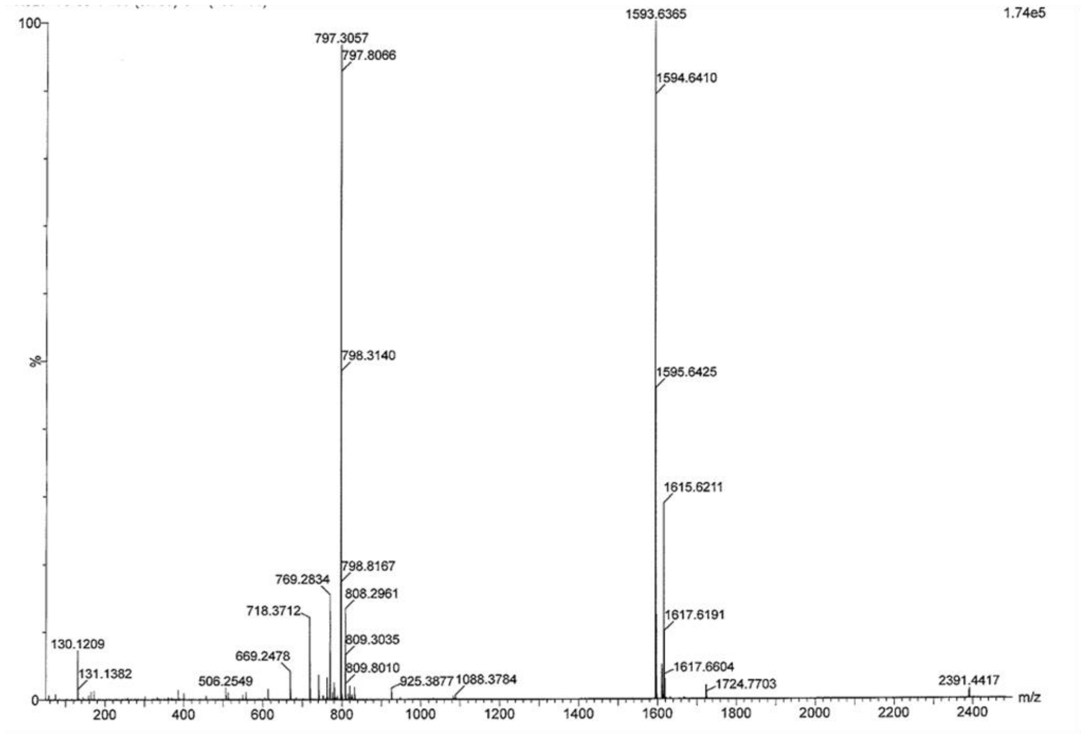

Figure S27. Analysis of compound **S-9a**, a) HPLC analysis, b) MS analysis

a)

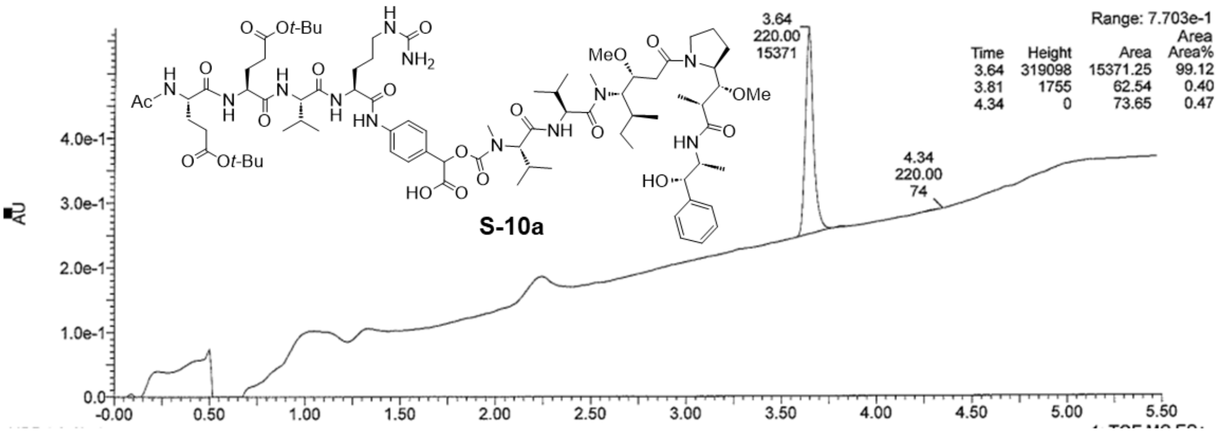

b)

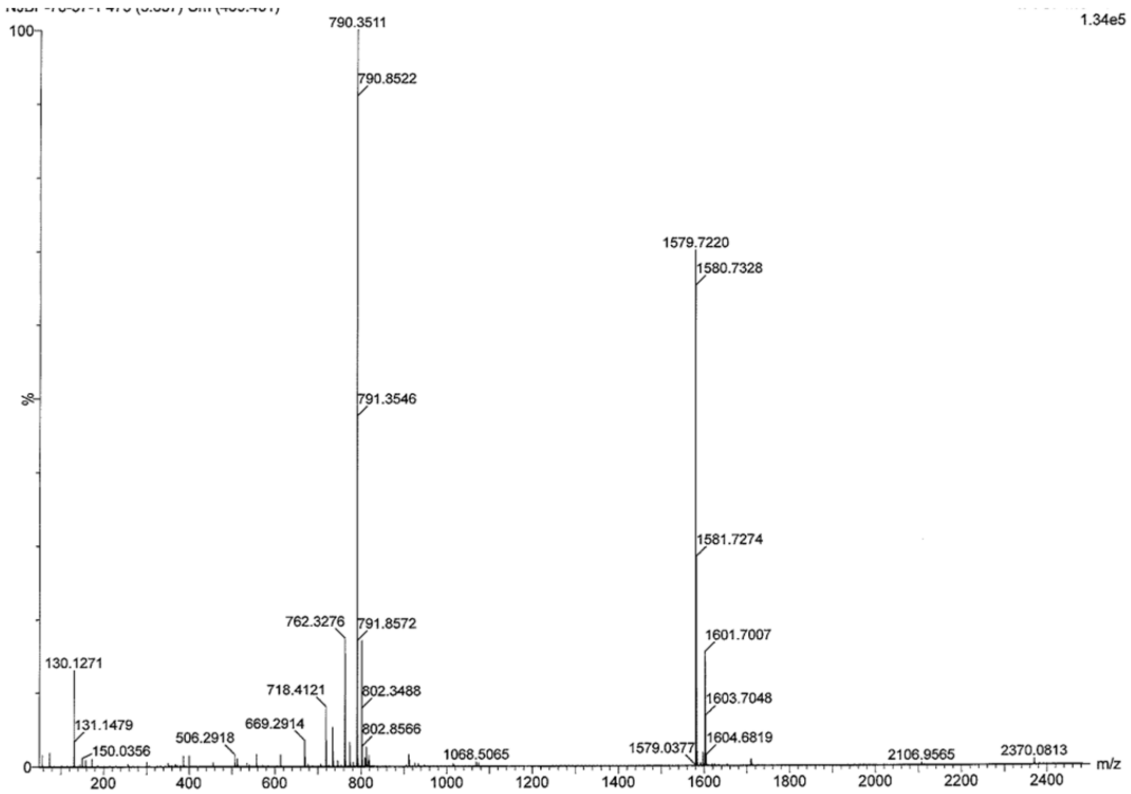

Figure S28. Analysis of compound **S-10a**, a) HPLC analysis, b) MS analysis

a)

b)

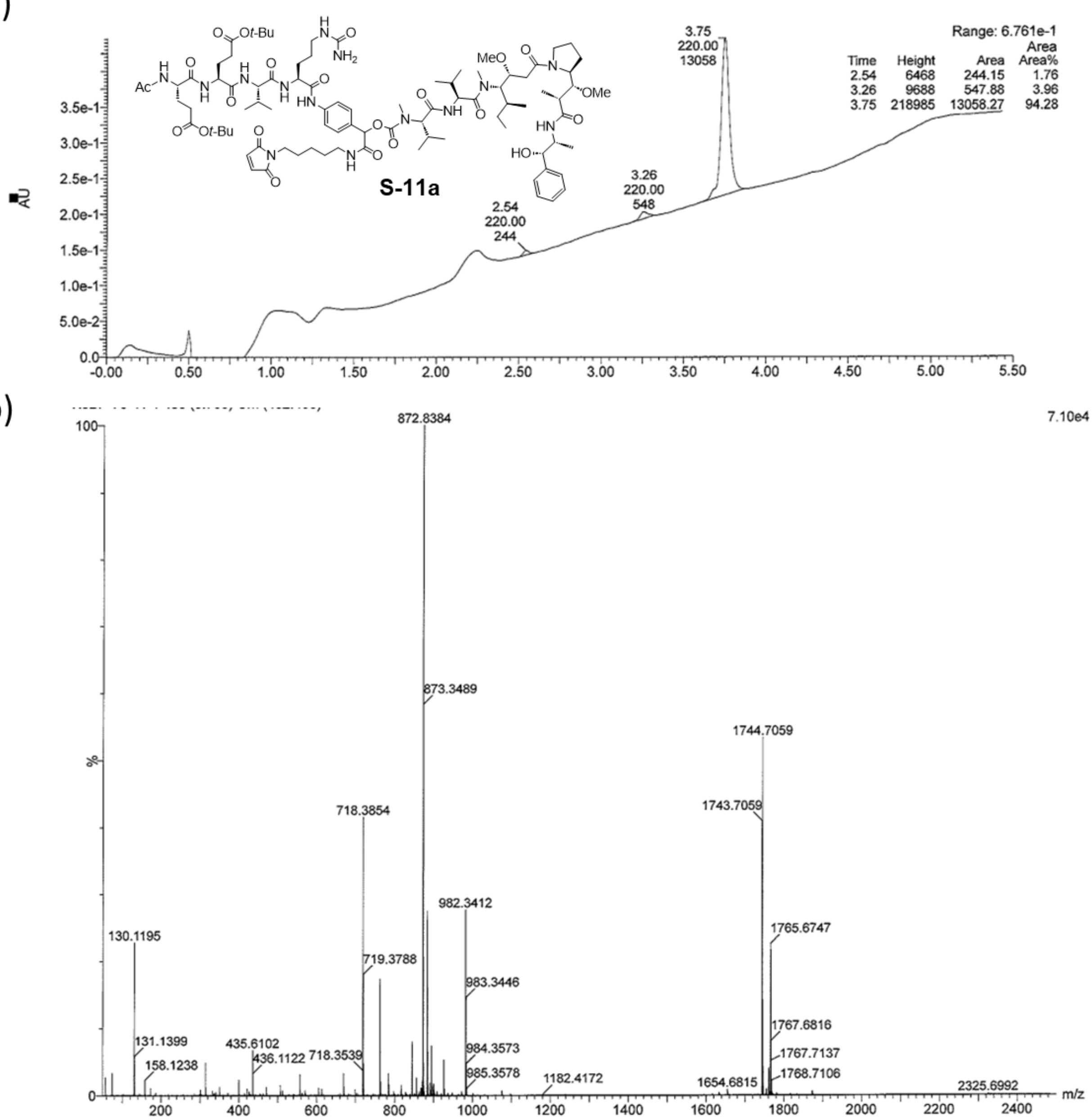

Figure S29. Analysis of compound **S-11a**, a) HPLC analysis, b) MS analysis

a)

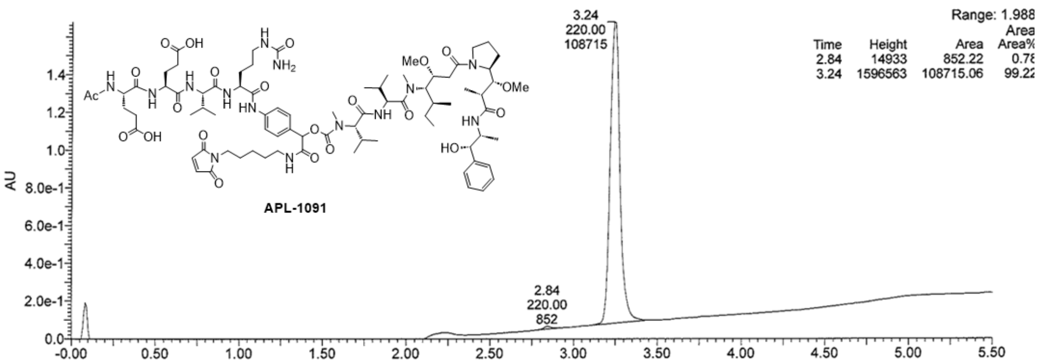

b)

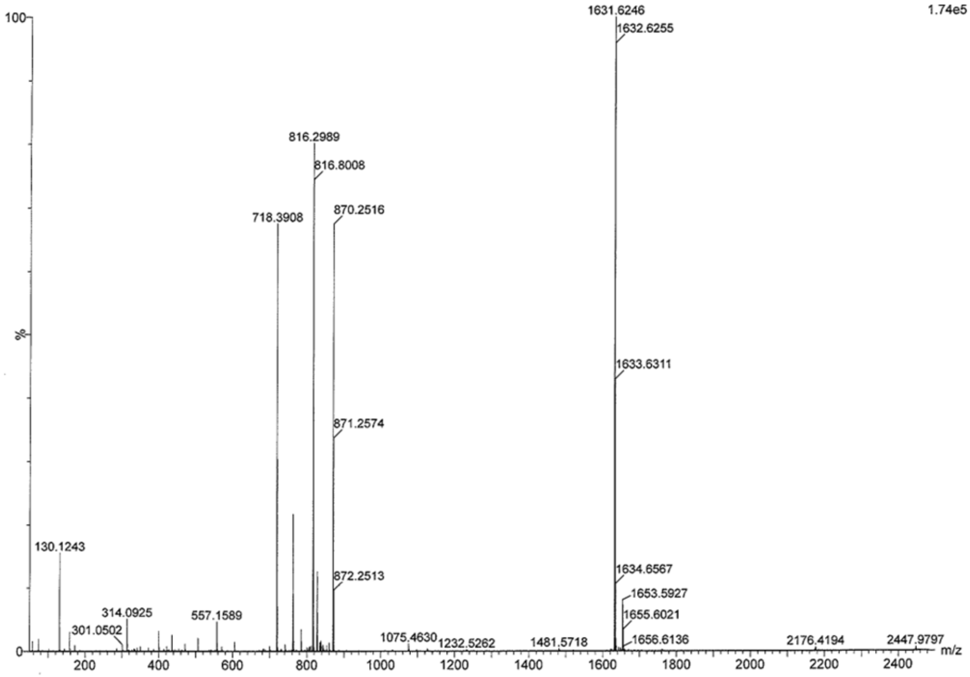

Figure S30. Analysis of compound APL-1091, a) HPLC analysis, b) MS analysis

a)

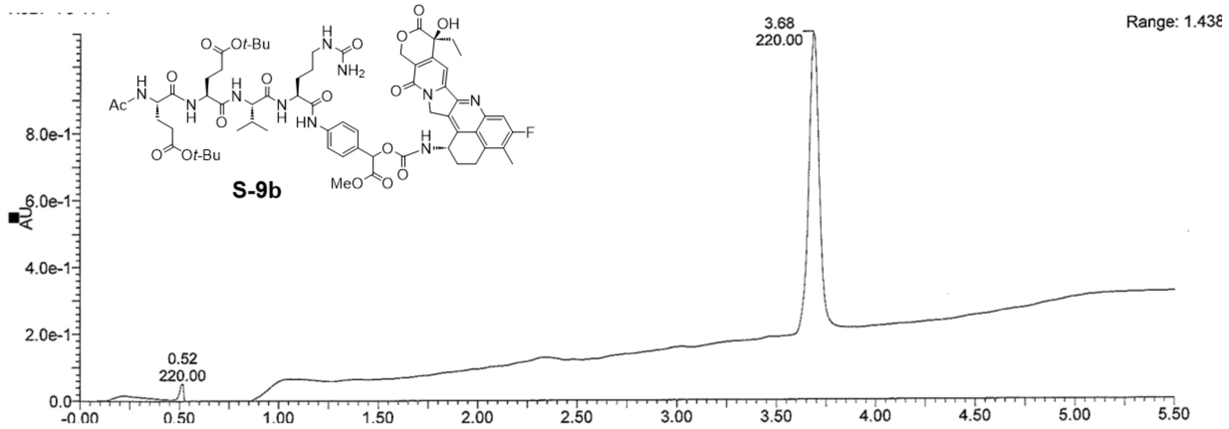

b)

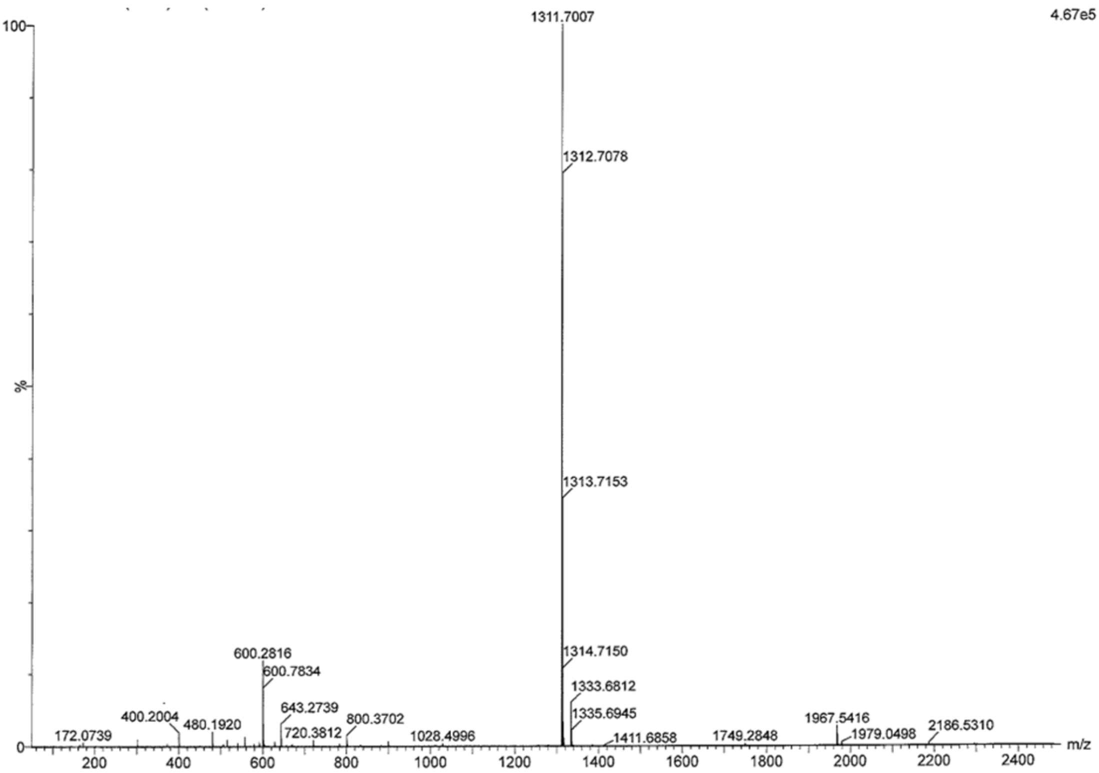

Figure S31. Analysis of compound **S-9b**, a) HPLC analysis, b) MS analysis

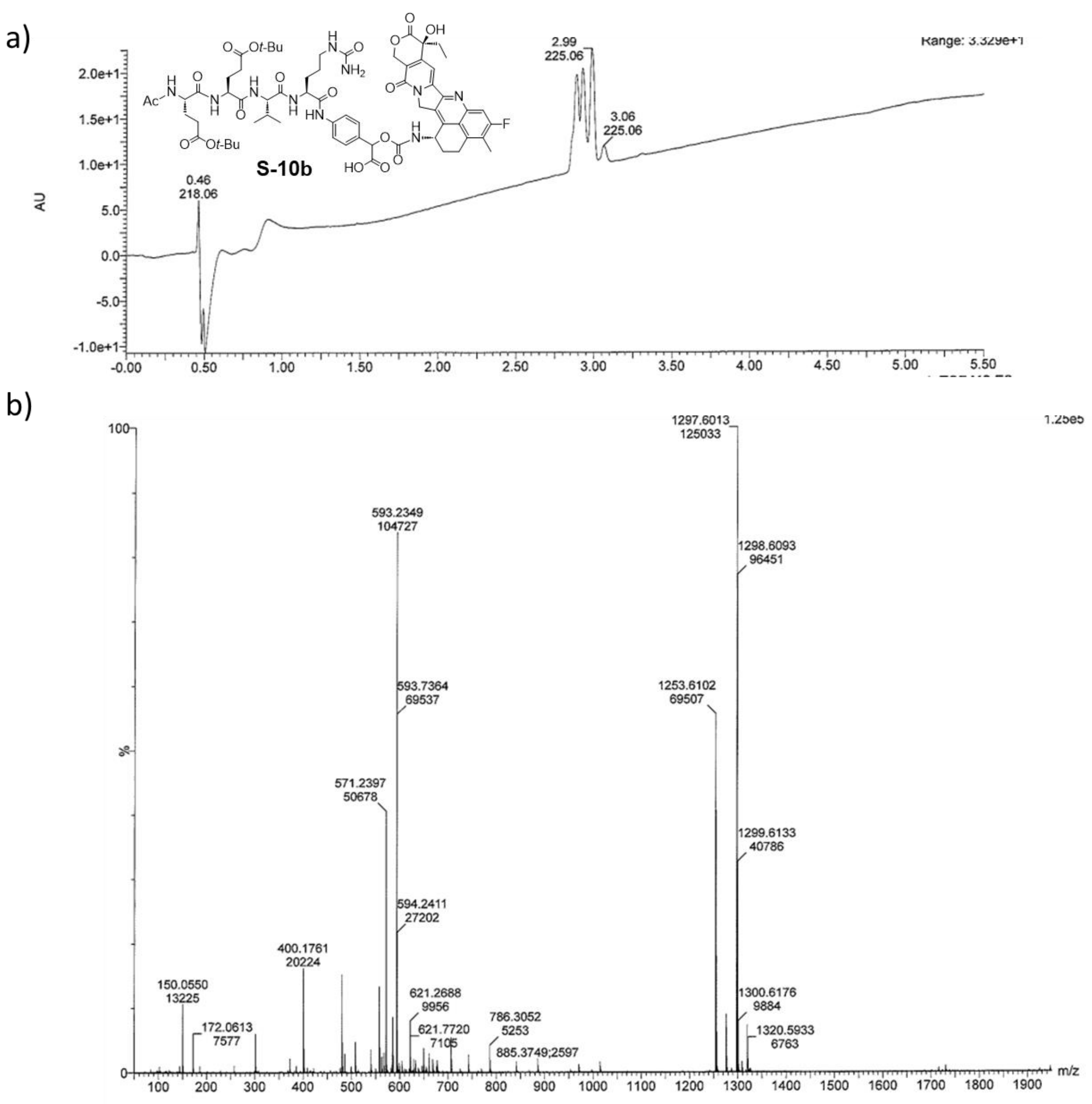

Figure S32. Analysis of compound **S-10b**, a) HPLC analysis, b) MS analysis

a)

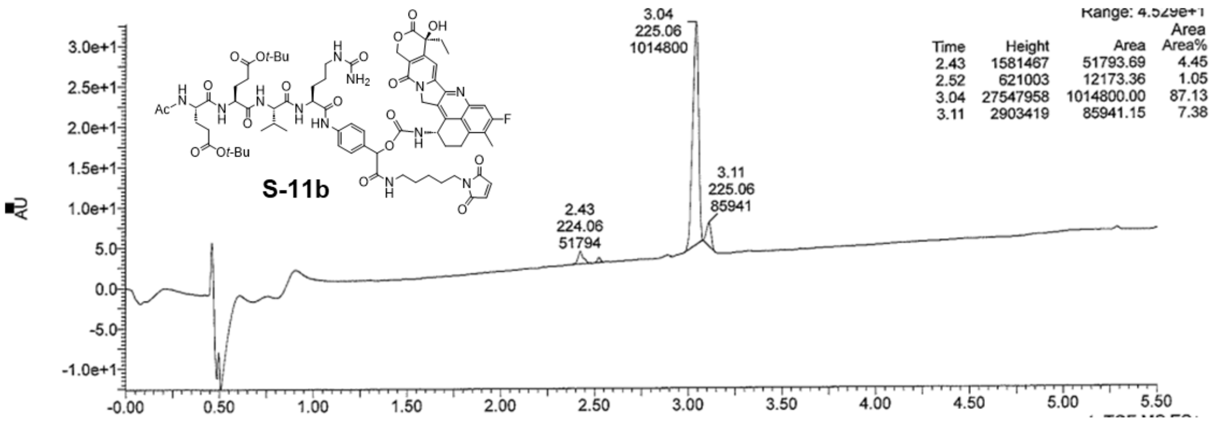

b)

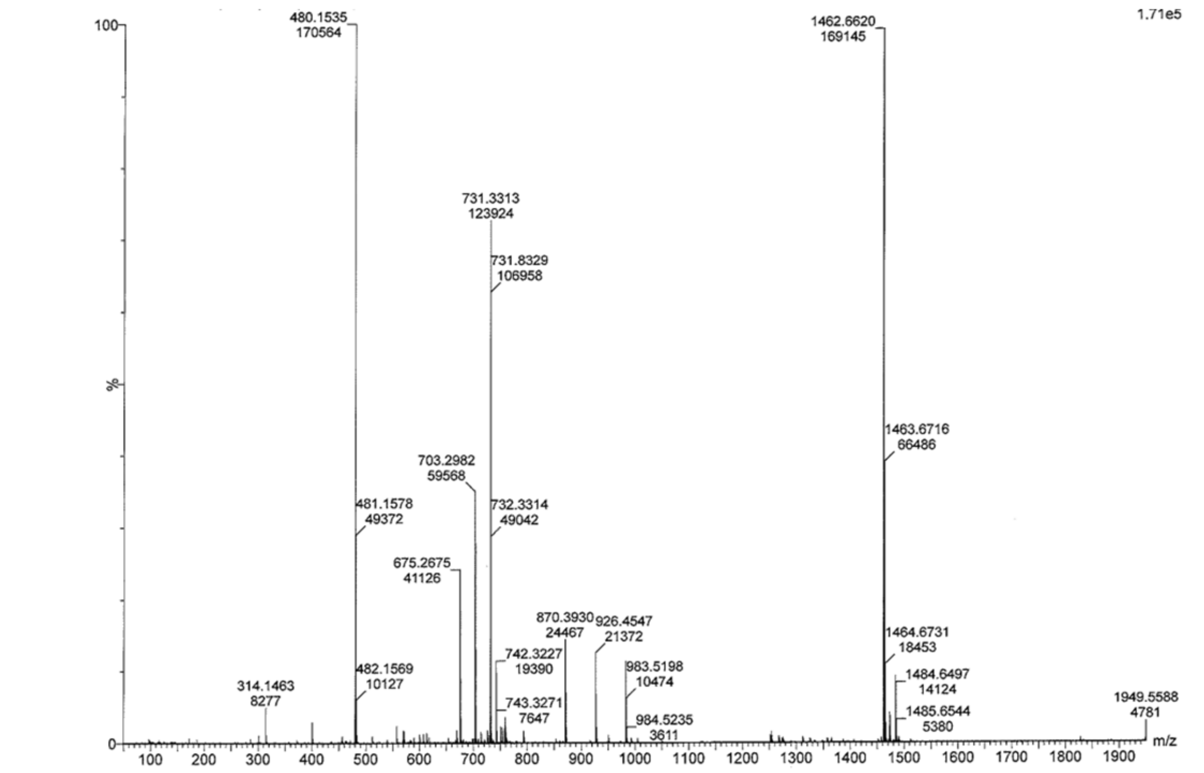

Figure S33. Analysis of compound **S-11b**, a) HPLC analysis, b) MS analysis

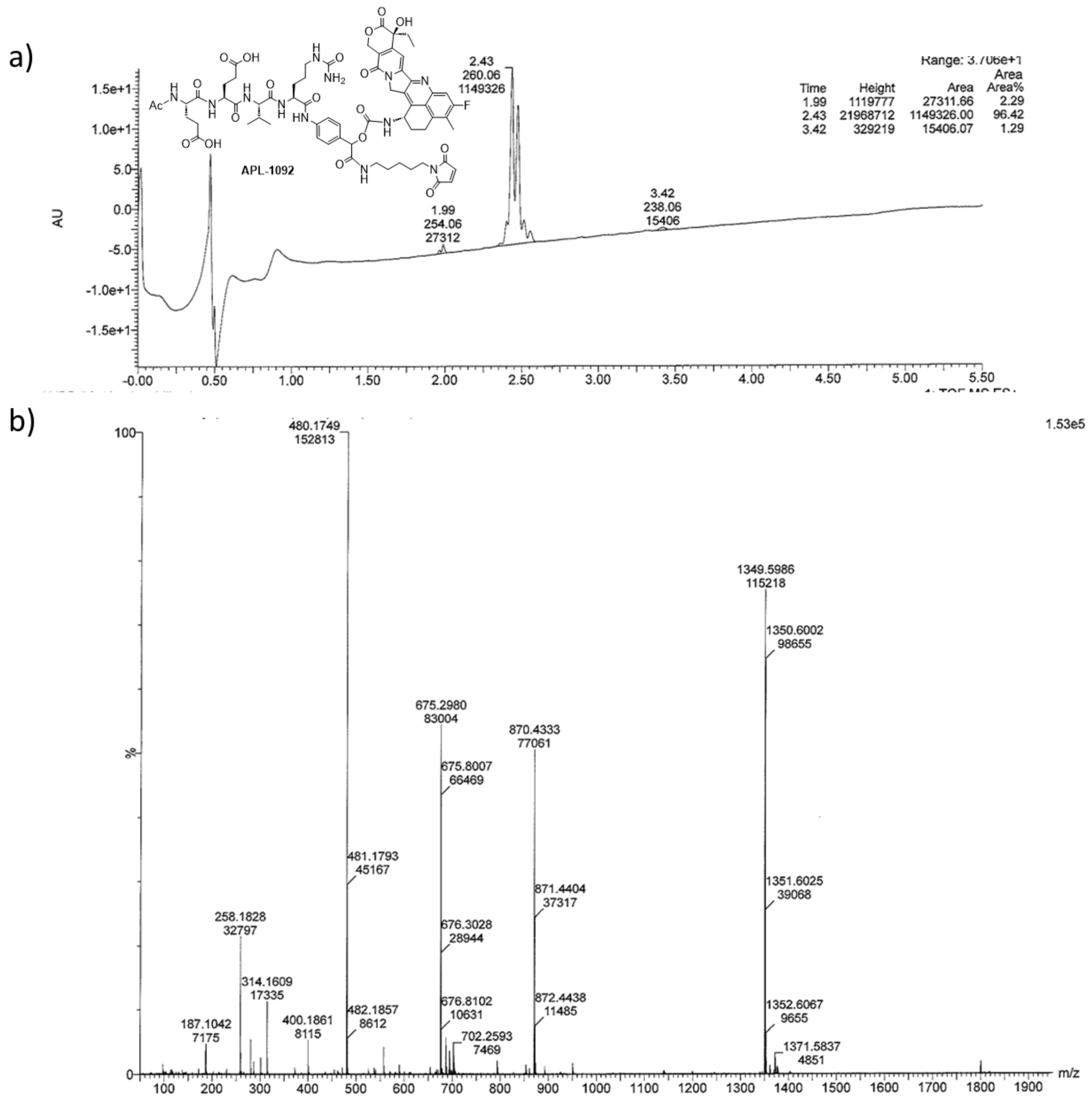

Figure S34. Analysis of compound APL-1092, a) HPLC analysis, b) MS analysis

Two main peaks were observed in HPLC, and MS confirmed that they have the same molecular weight. This suggests that they are diastereomers derived from the secondary alcohol used to construct the carbamate.

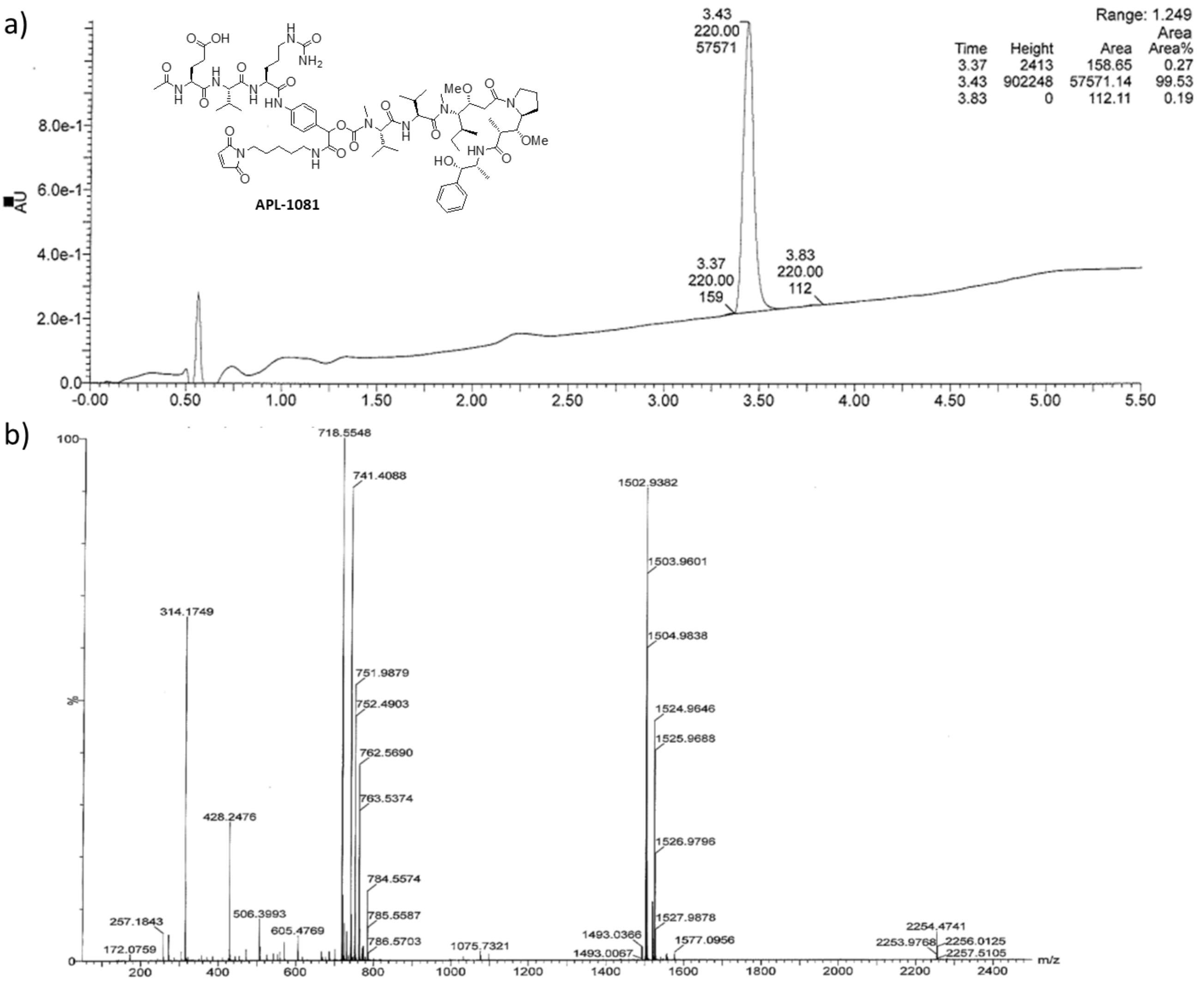

Figure S35. Analysis of compound APL-1081, a) HPLC analysis, b) MS analysis

2 ADC synthesis: HIC and SEC analysis

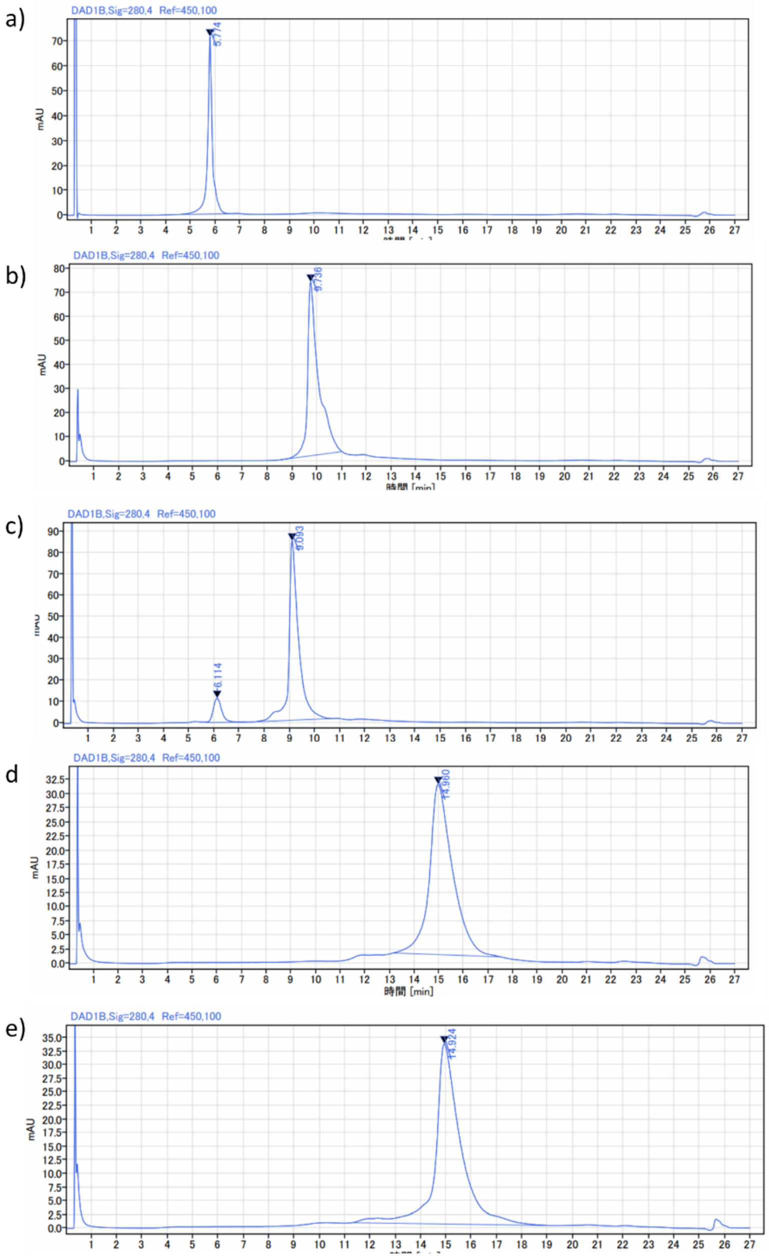

Figure S36. HIC-HPLC analysis of pyrene-based ADCs, a) trastuzumab, b) ADC (1) (Mc-Exo-EVC-pyrene), c) ADC (2) (Mc-Exo-EEVC-pyrene), d) ADC (3) (Mc-VC-PAB-pyrene), e) ADC (4) (Mc-EVC-PAB-pyrene)

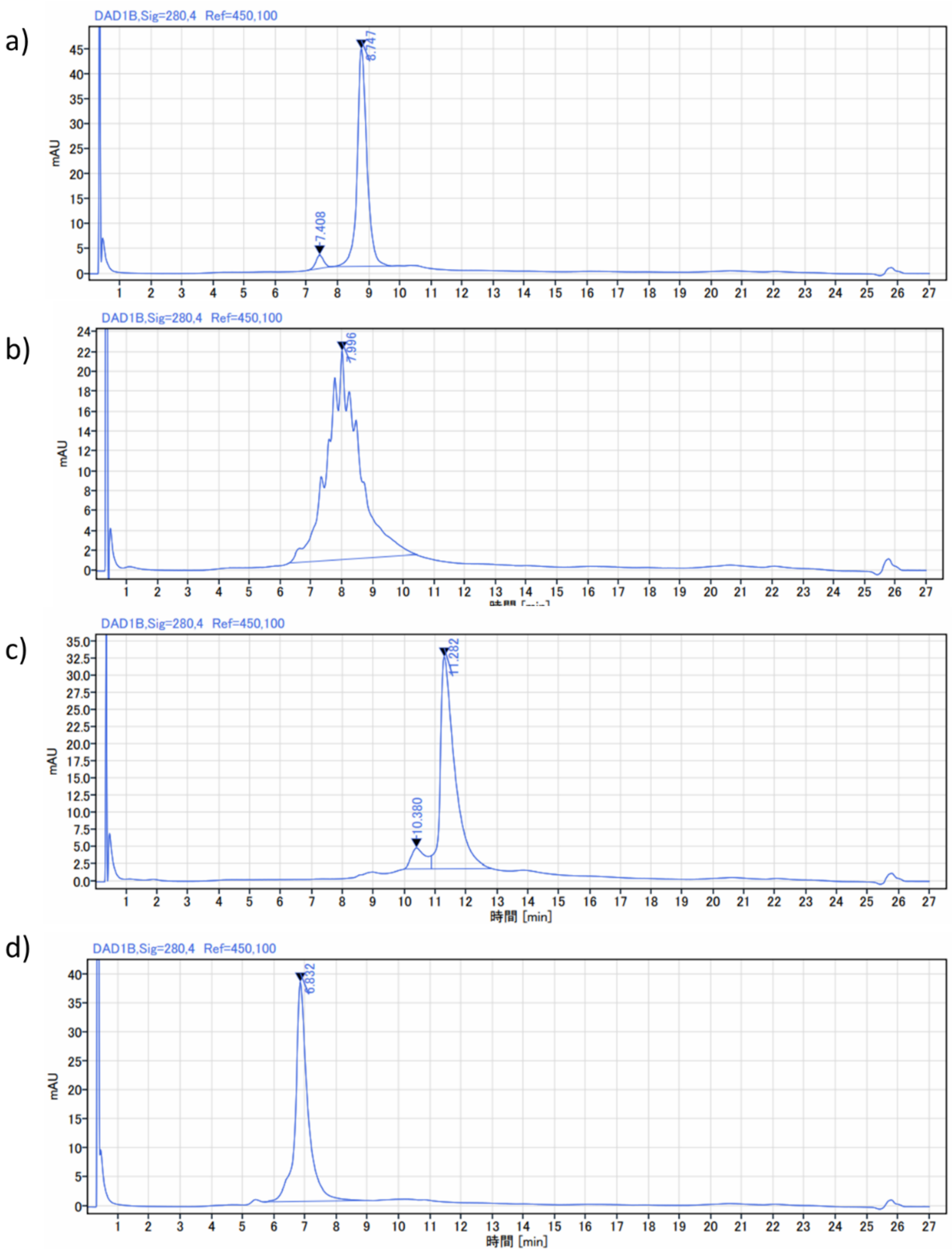

Figure S37. HIC-HPLC analysis of cytotoxic payload-based ADCs, a) ADC (6) (APL-1091, DAR=2), b) ADC (7) (APL-1092, DAR=2), c) ADC (8) (APL-1091, DAR=8), d) ADC (9) (APL-1092, DAR=8)

a)

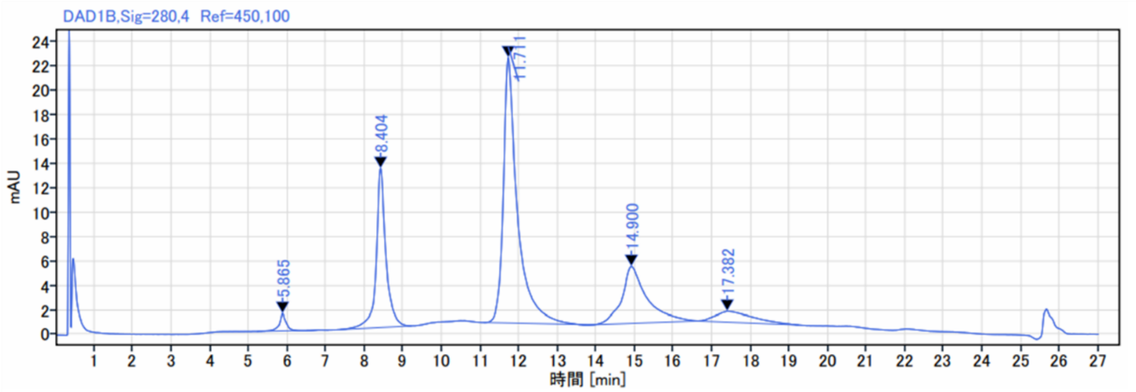

b)

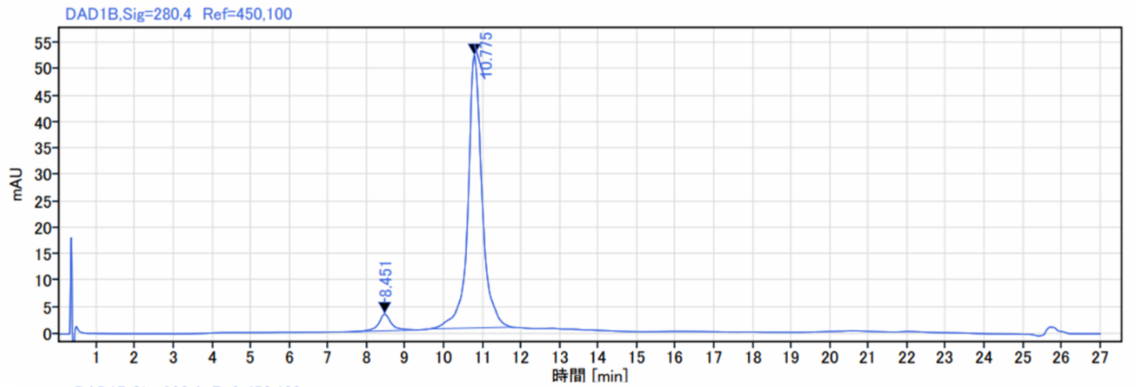

c)

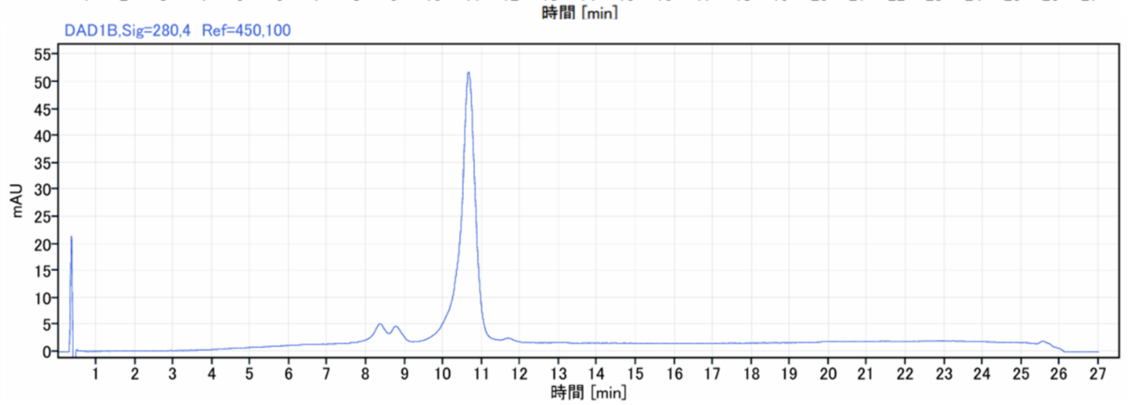

d)

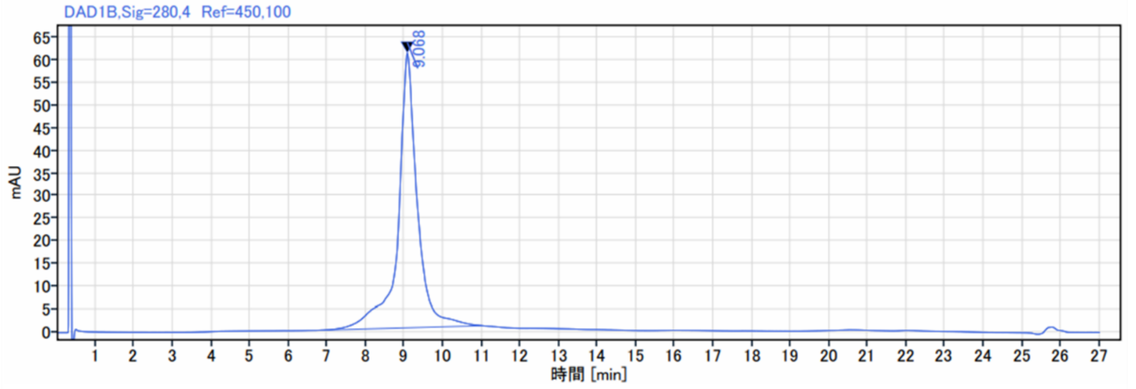

Figure S38. HIC-HPLC analysis of benchmark ADCs, a) ADC (10) (Mc-VC-PAB-MMAE, DAR=4), b) ADC (11) (Mc-VC-PAB-MMAE, DAR=2), c) ADC (12) (Mc-EVC-PAB-MMAE, DAR=2), d) T-Dxd (deruxtecan, DAR=8),

a)

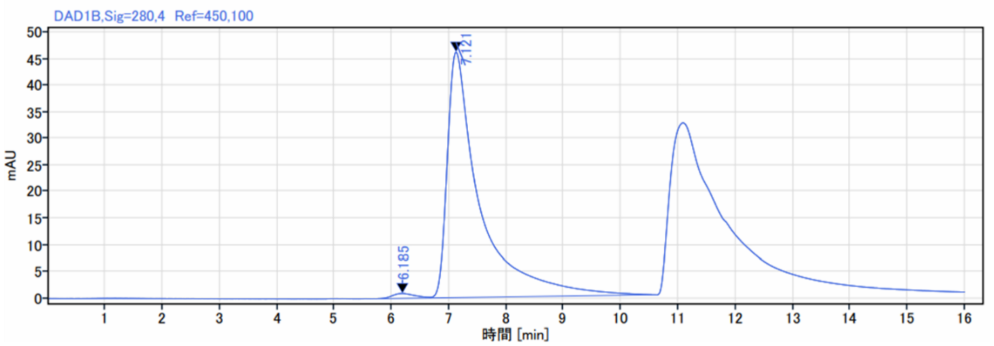

b)

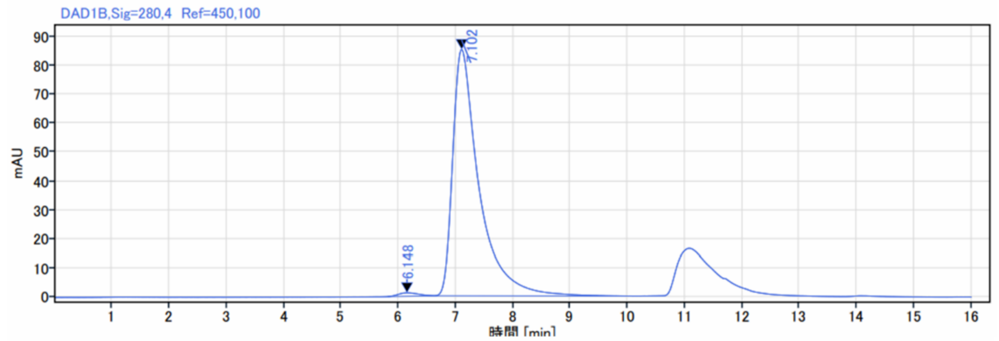

c)

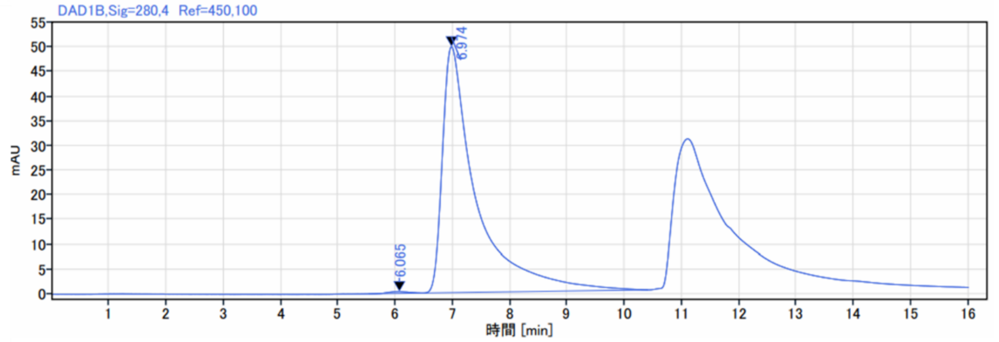

d)

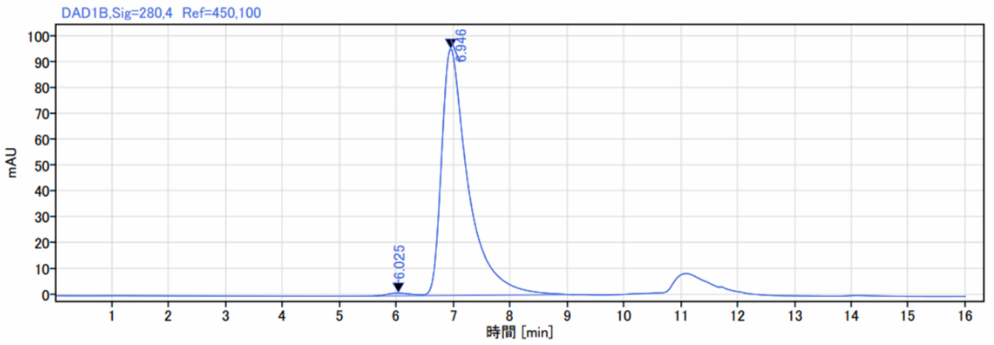

Figure S39. SEC-HPLC analysis of cytotoxic payload-based ADCs, a) ADC (6) (APL-1091, DAR=2), b) ADC (7) (APL-1092, DAR=2), c) ADC (8) (APL-1091, DAR=8), d) ADC (9) (APL-1092, DAR=8)

3 In vivo xenograft study

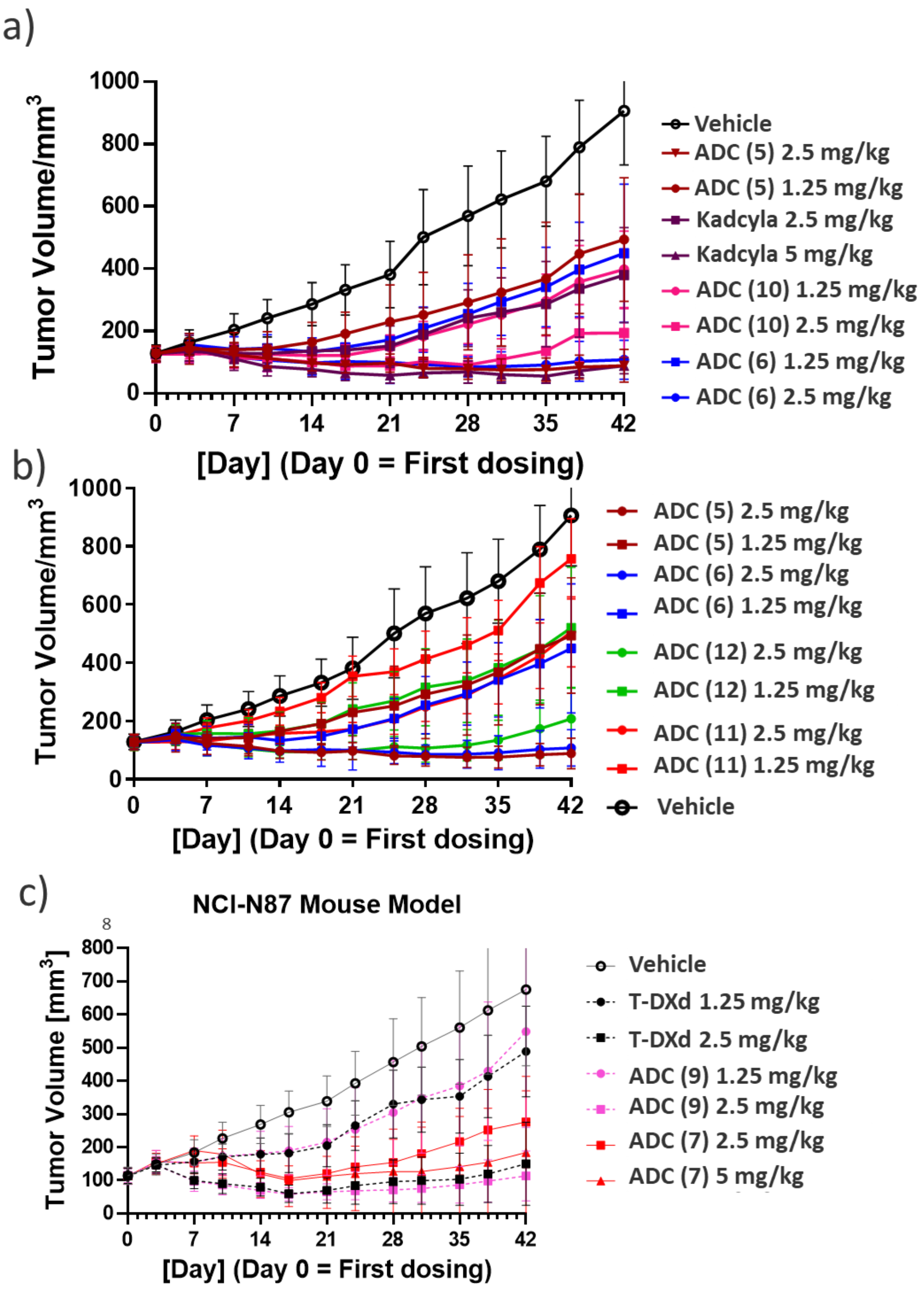

Figure S40. Anti-tumor activity of anti-HER2 ADCs in a NCI-N87 xenograft tumor model: a) Comparison of MMAE-based ADCs compared with Kadcyla, b) MMAE-based ADCs to compare the cleavable linker, c) Comparions of Exatecan-based ADCs.

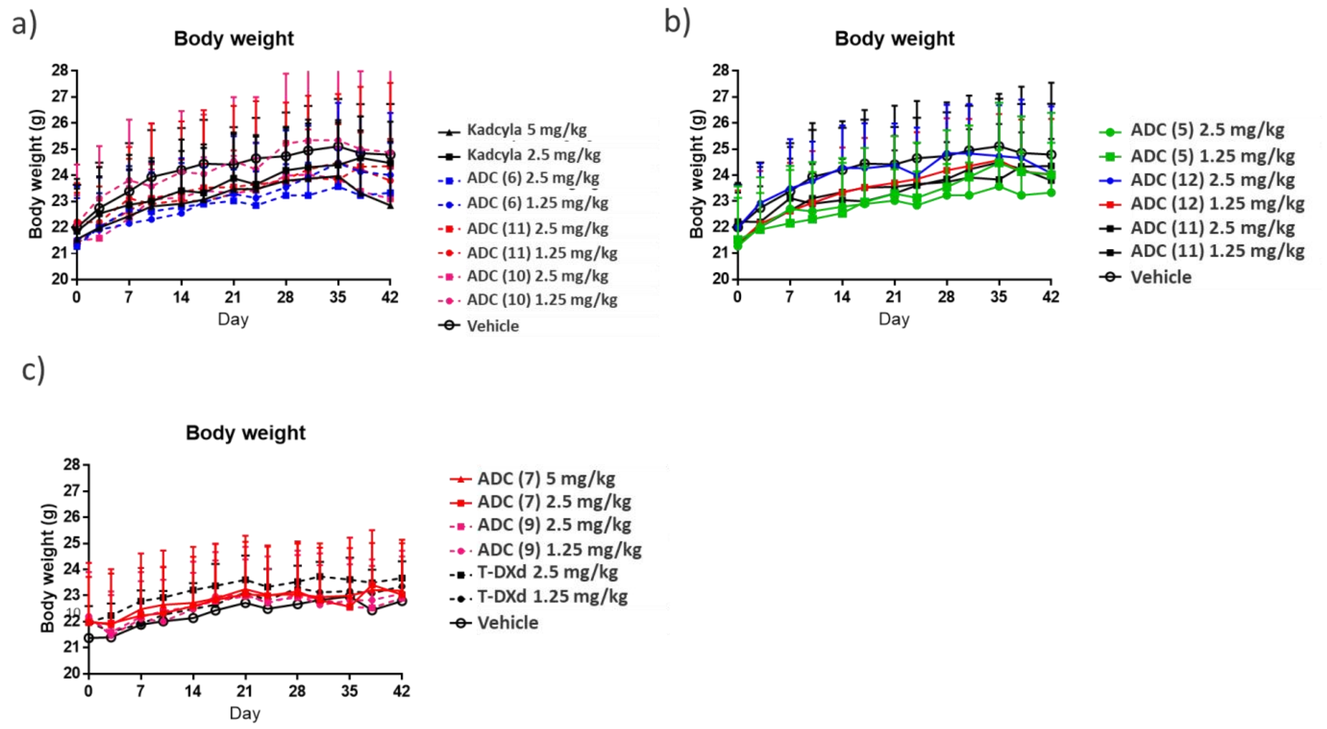

Figure S41. Relative weight (note tight Y-axis) of groups in the study. : a) Comparison of MMAE-based ADCs compared with Kadcyla, b) MMAE-based ADCs to compare the cleavable linker, c) Comparions of Exatecan-based ADCs.

All groups gained weight over the course of the study (roughly 10-20% increase in body weight for all groups). This would be expected for young mice that were used in the study.

4 Rat PK study

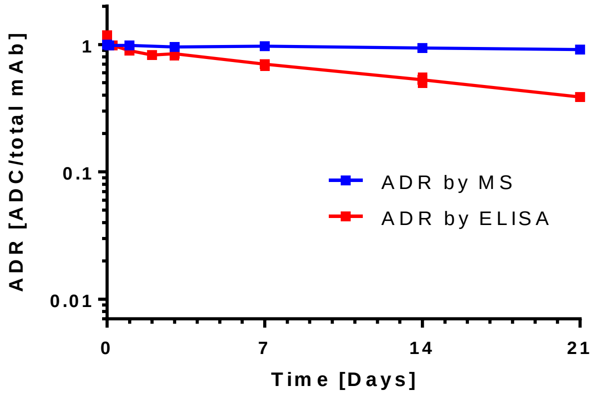

Figure S42. Comparison of LC-MS assay and ELISA assay of ADC 11

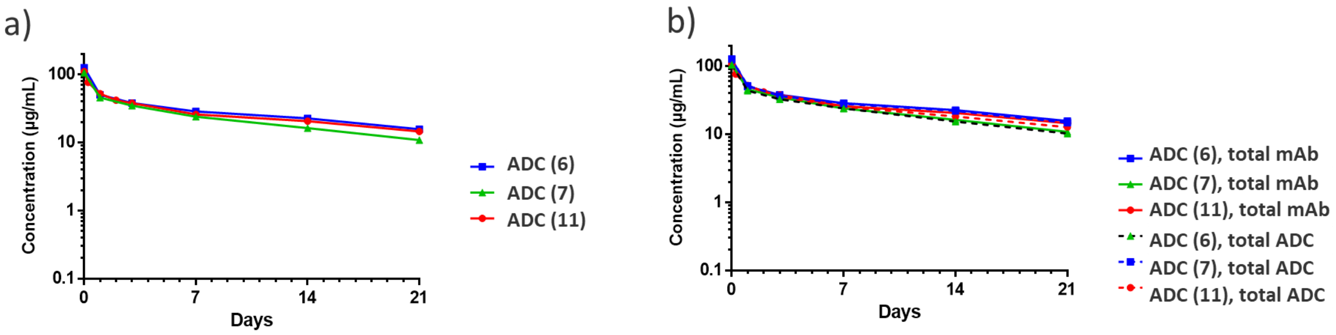

Figure S43. Pharmacokinetic Study of exo-linker-based ADCs in Rats. a) Analysis of total antibody using ELISA. b) Combined trend of total antibody and total ADC. Trend in average DAR determined via LC-MS assay follow by multiplying the average DAR by the total antibody concentration to caluculate Total ADC.

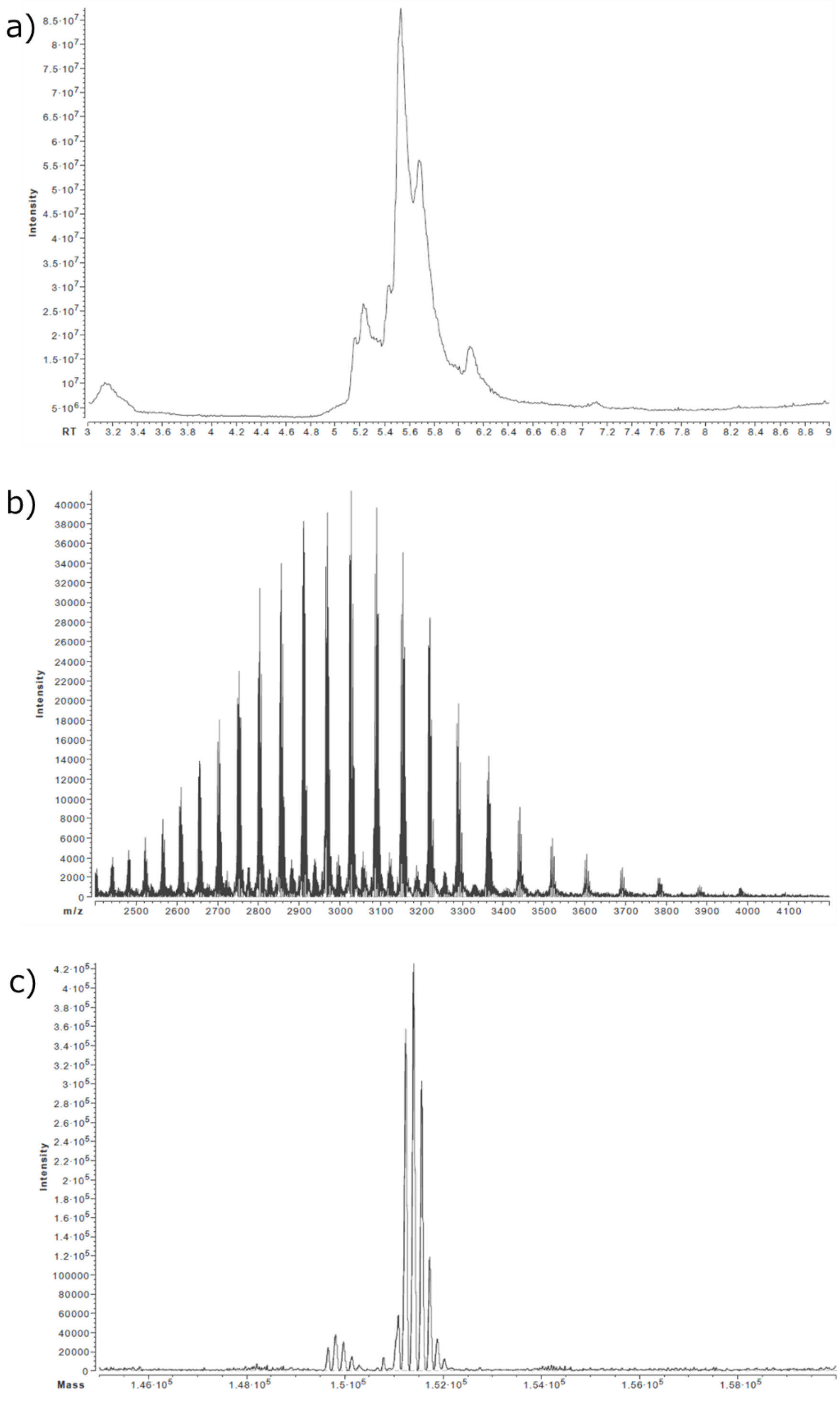

Figure S44. Provide a representative data of the n3 analysis of ADC 5 Day0, a) TIC within a retention time range of 3 to 9 minutes, b) Spectrum of *m/z* 2400 to 4200, c) Deconvoluted spectrum ranging from 1.45e5 to 1.60e5.

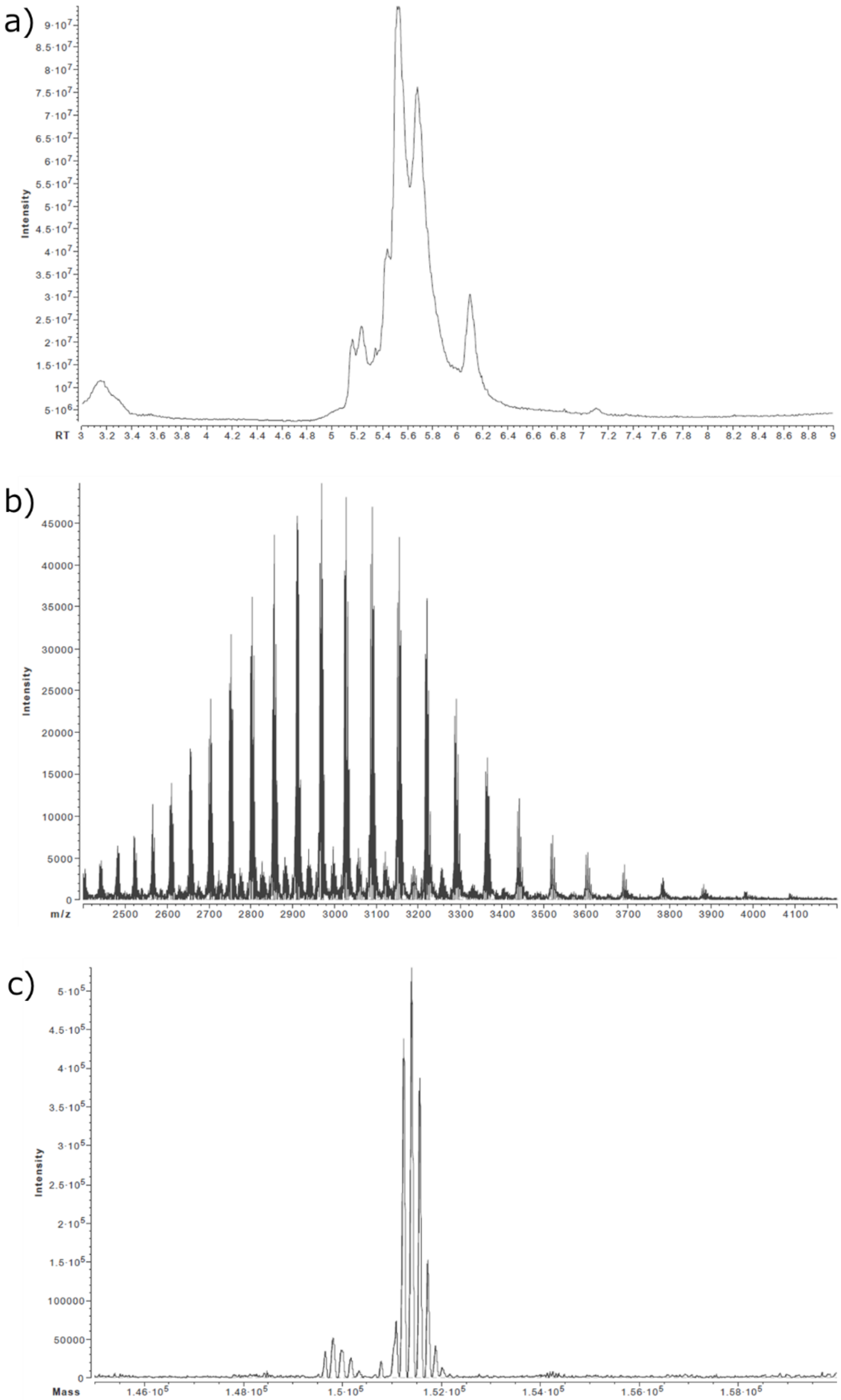

Figure S45. Provide a representative data of the n3 analysis of ADC 5, a) TIC within a retention time range of 3 to 9 minutes, b) Spectrum of *m/z* 2400 to 4200, c) Deconvoluted spectrum ranging from 1.45e5 to 1.60e5.

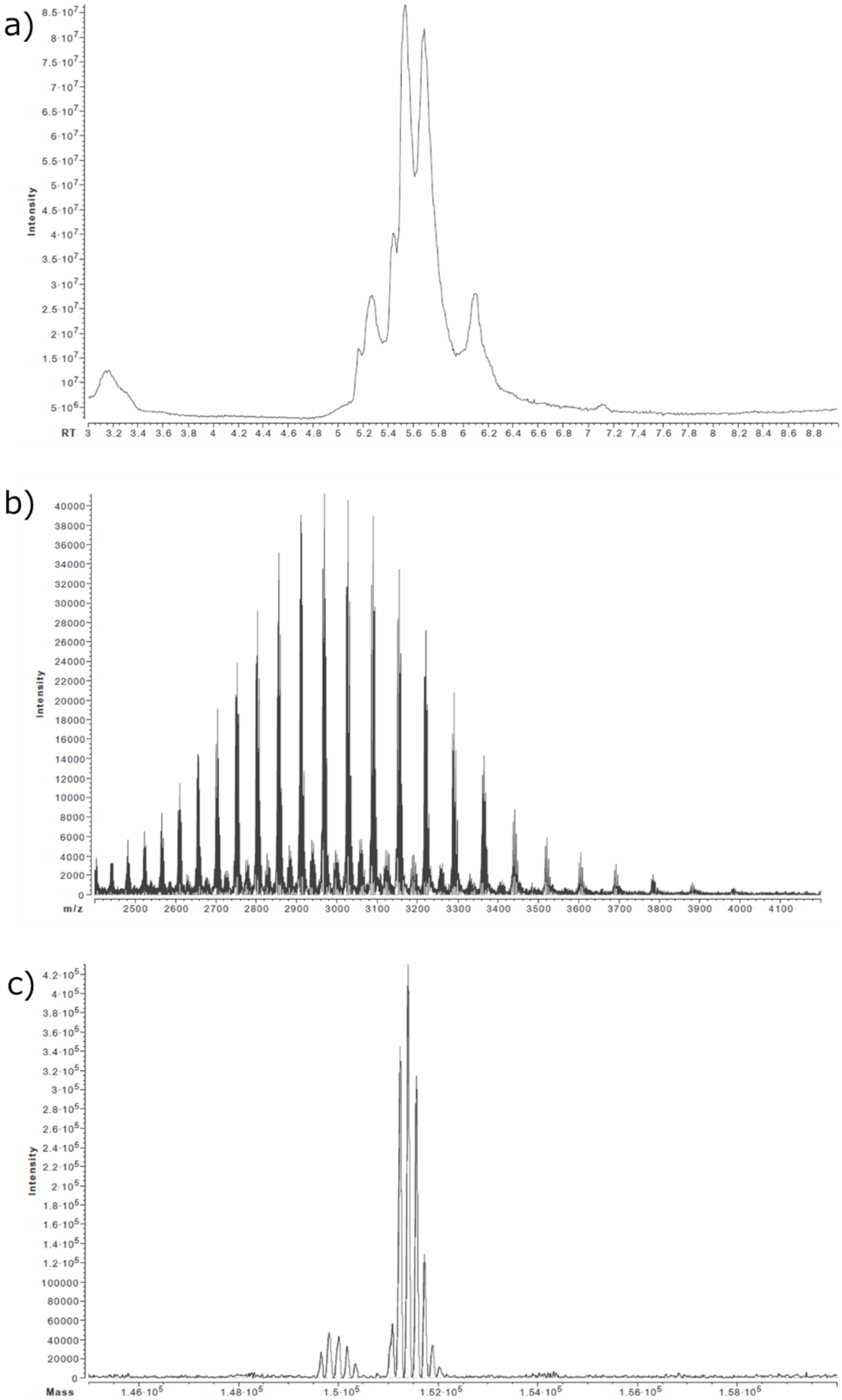

Figure S46. Provide a representative data of the n3 analysis of ADC 5, a) TIC within a retention time range of 3 to 9 minutes, b) Spectrum of *m/z* 2400 to 4200, c) Deconvoluted spectrum ranging from 1.45e5 to 1.60e5.

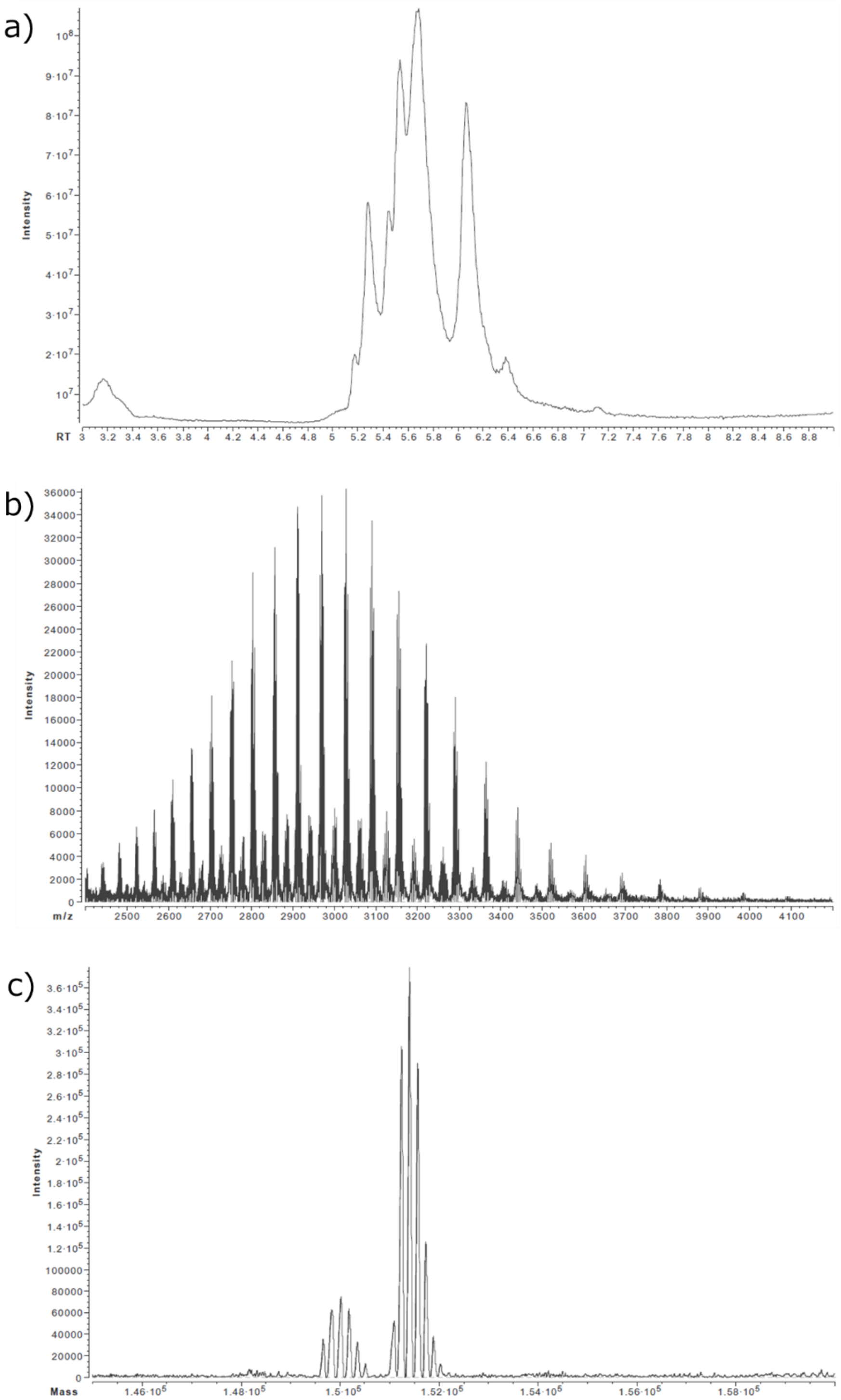

Figure S47. Provide a representative data of the n3 analysis of ADC 5 Day7, a) TIC within a retention time range of 3 to 9 minutes, b) Spectrum of *m/z* 2400 to 4200, c) Deconvoluted spectrum ranging from 1.45e5 to 1.60e5.

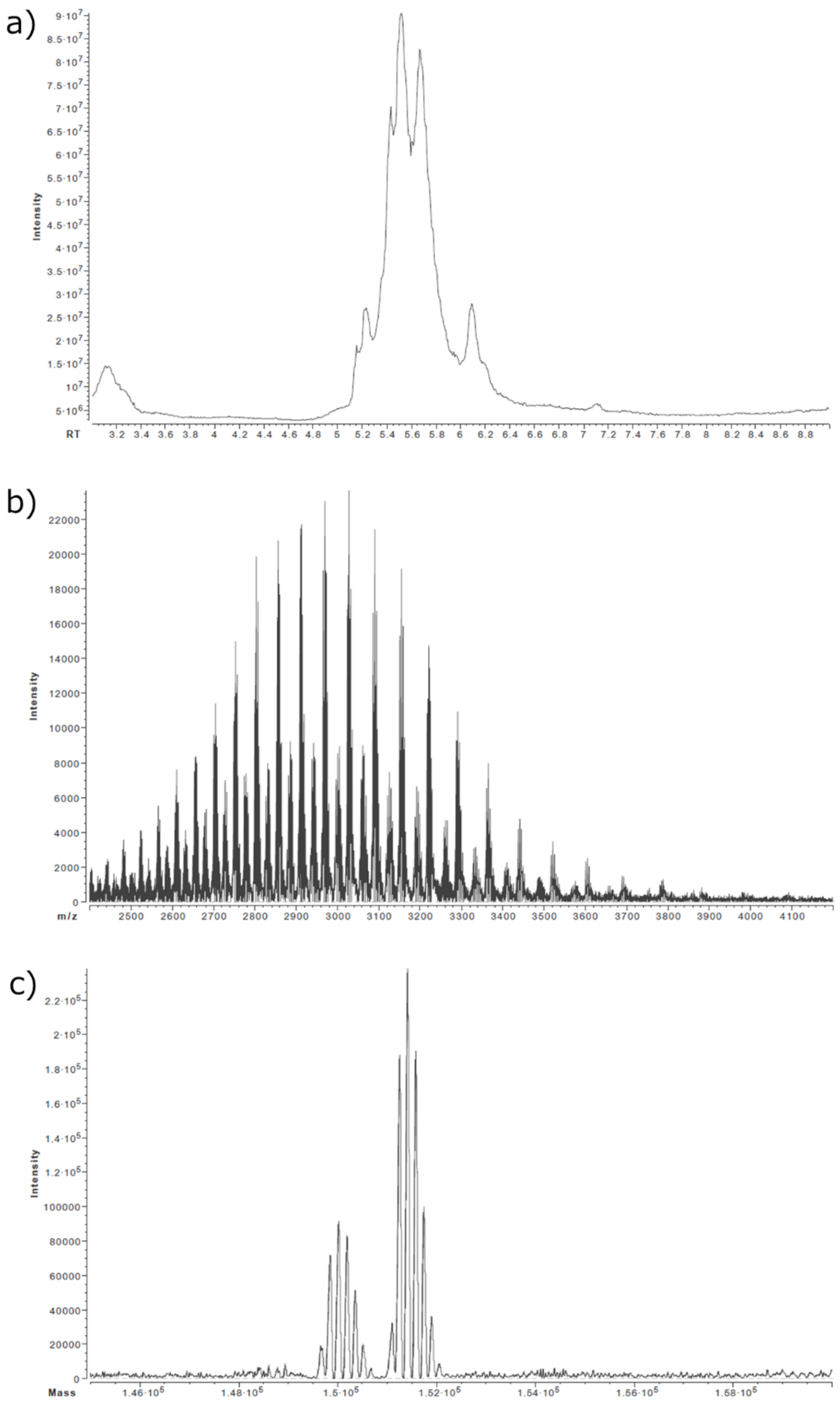

Figure S48. Provide a representative data of the n3 analysis of ADC 5 Day21, a) TIC within a retention time range of 3 to 9 minutes, b) Spectrum of *m/z* 2400 to 4200, c) Deconvoluted spectrum ranging from 1.45e5 to 1.60e5.

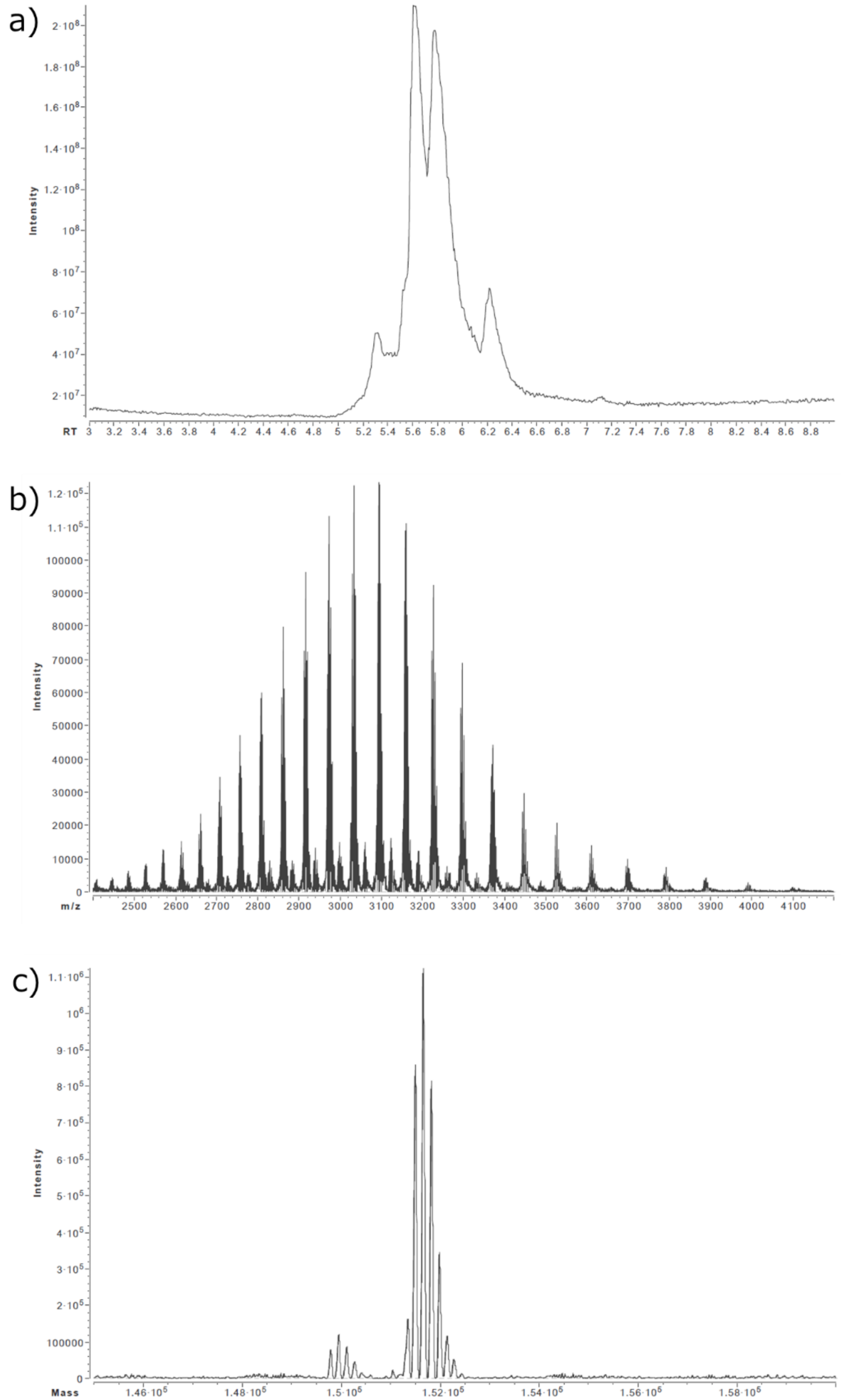

Figure S49. Provide a representative data of the n3 analysis of ADC 6 Day0, a) TIC within a retention time range of 3 to 9 minutes, b) Spectrum of *m/z* 2400 to 4200, c) Deconvoluted spectrum ranging from 1.45e5 to 1.60e5.

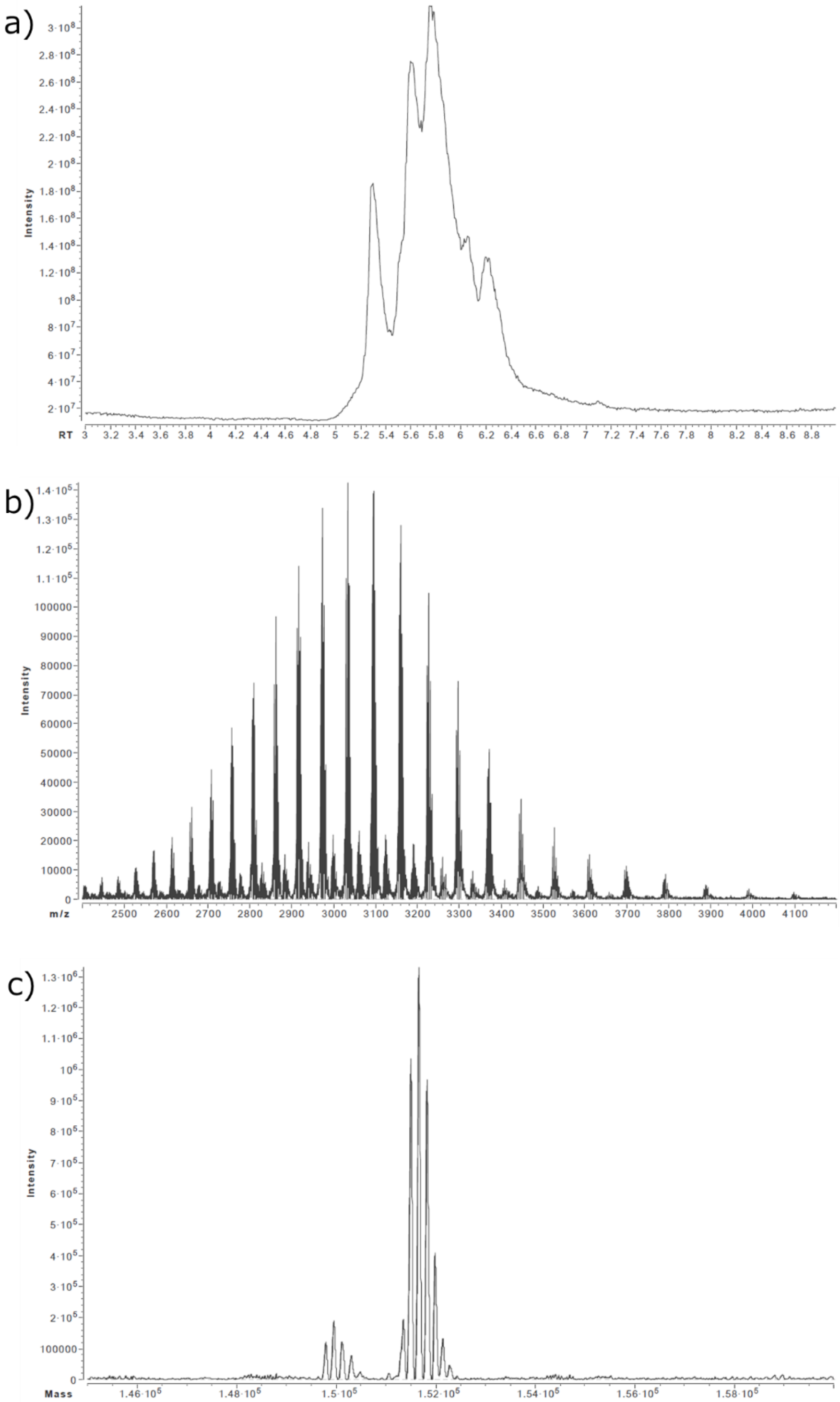

Figure S50. Provide a representative data of the n3 analysis of ADC 6 Day1, a) TIC within a retention time range of 3 to 9 minutes, b) Spectrum of *m/z* 2400 to 4200, c) Deconvoluted spectrum ranging from 1.45e5 to 1.60e5.

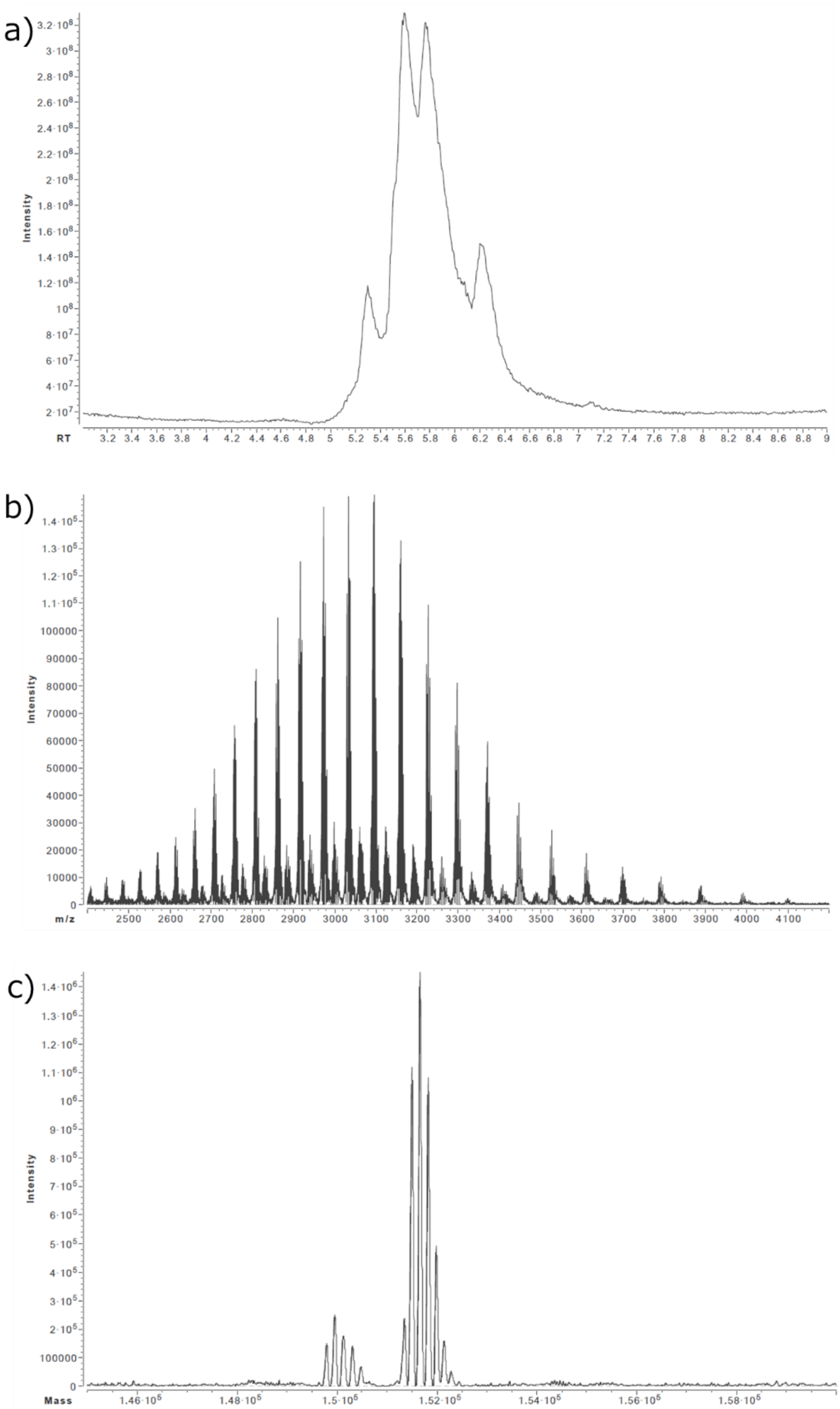

Figure S51. Provide a representative data of the n3 analysis of ADC 6 Day3, a) TIC within a retention time range of 3 to 9 minutes, b) Spectrum of *m/z* 2400 to 4200, c) Deconvoluted spectrum ranging from 1.45e5 to 1.60e5.

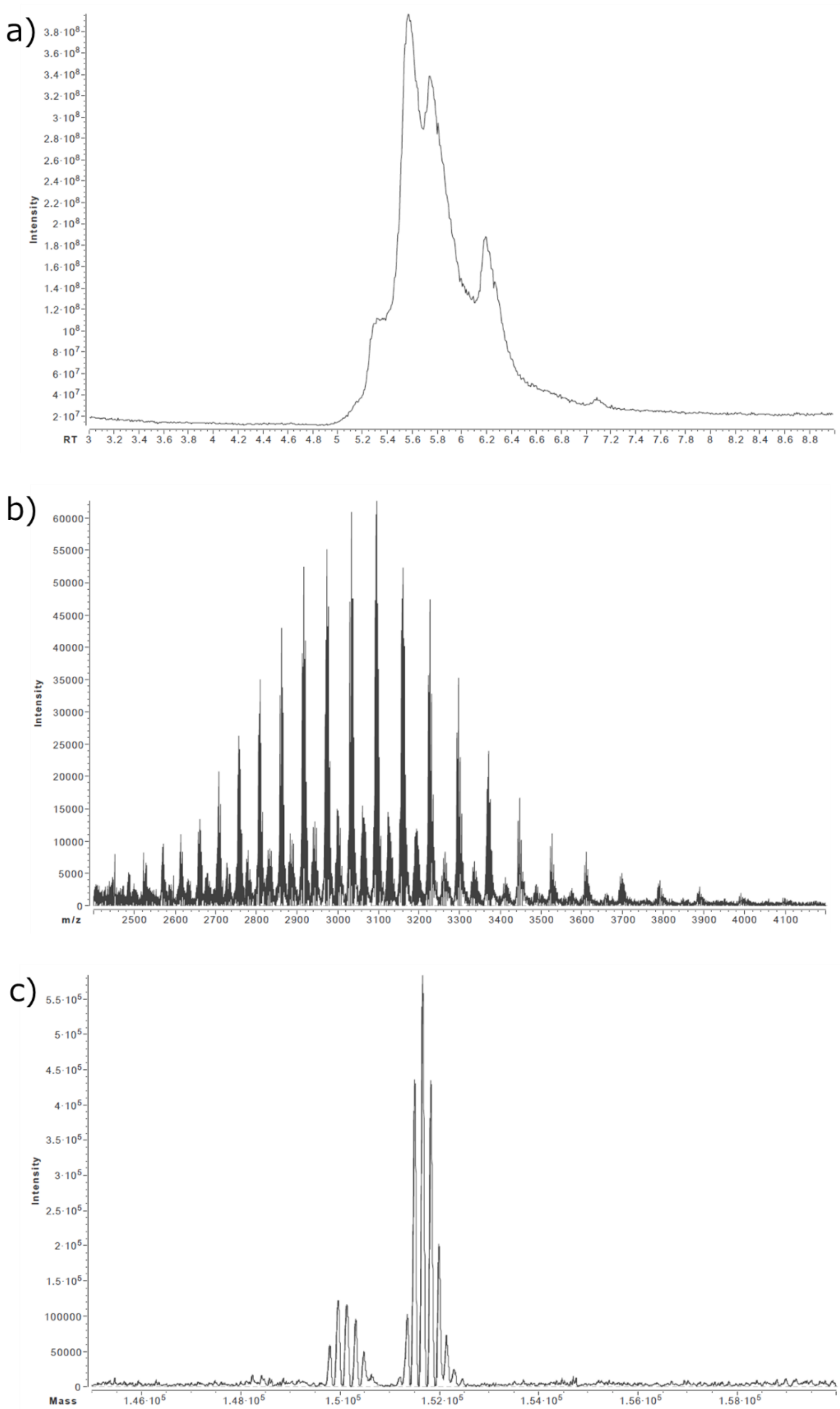

Figure S52. Provide a representative data of the n3 analysis of ADC 6 Day7, a) TIC within a retention time range of 3 to 9 minutes, b) Spectrum of *m/z* 2400 to 4200, c) Deconvoluted spectrum ranging from 1.45e5 to 1.60e5.

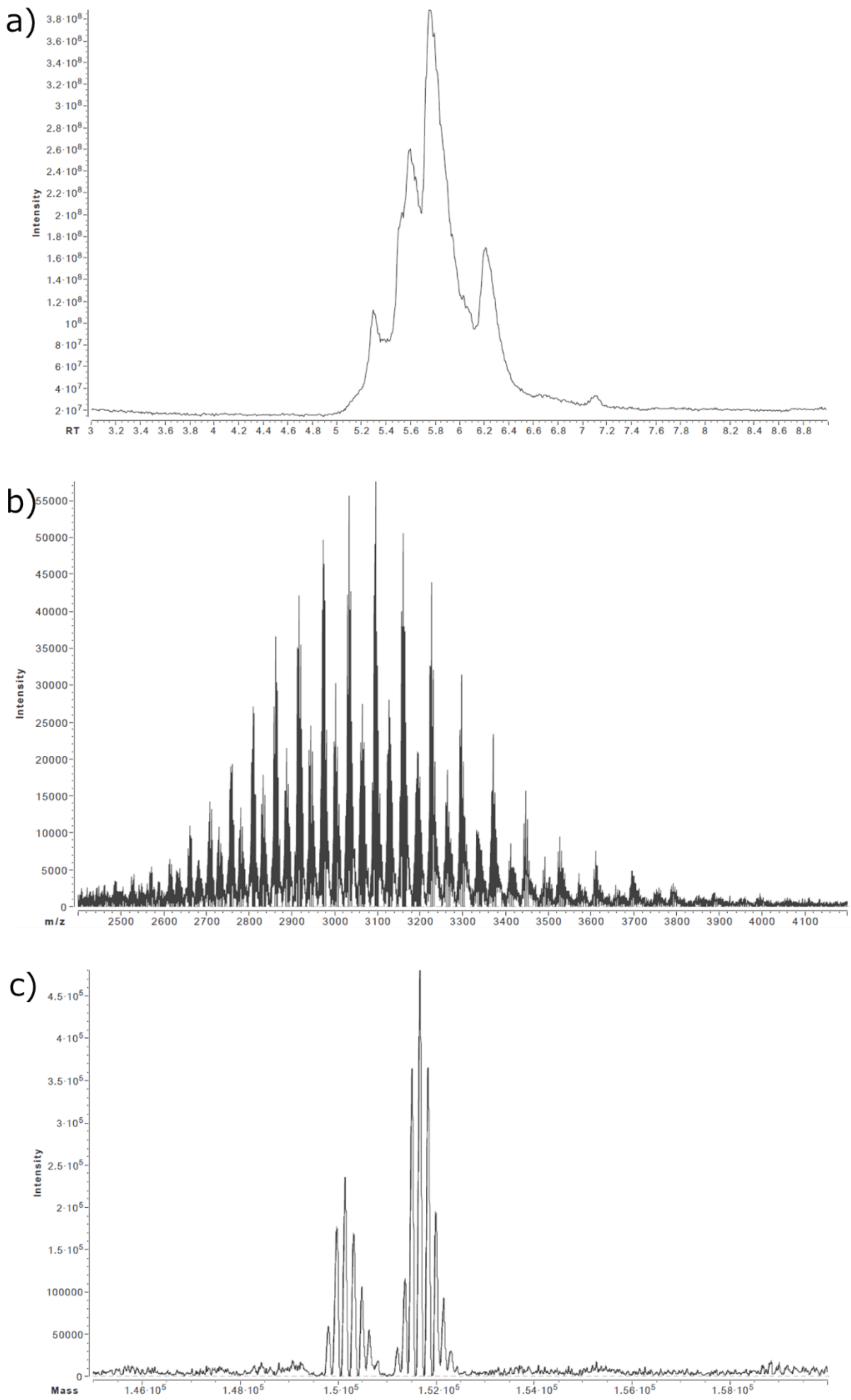

Figure S53. Provide a data of the analysis of ADC 6 Day21, a) TIC within a retention time range of 3 to 9 minutes, b) Spectrum of *m/z* 2400 to 4200, c) Deconvoluted spectrum ranging from 1.45e5 to 1.60e5.

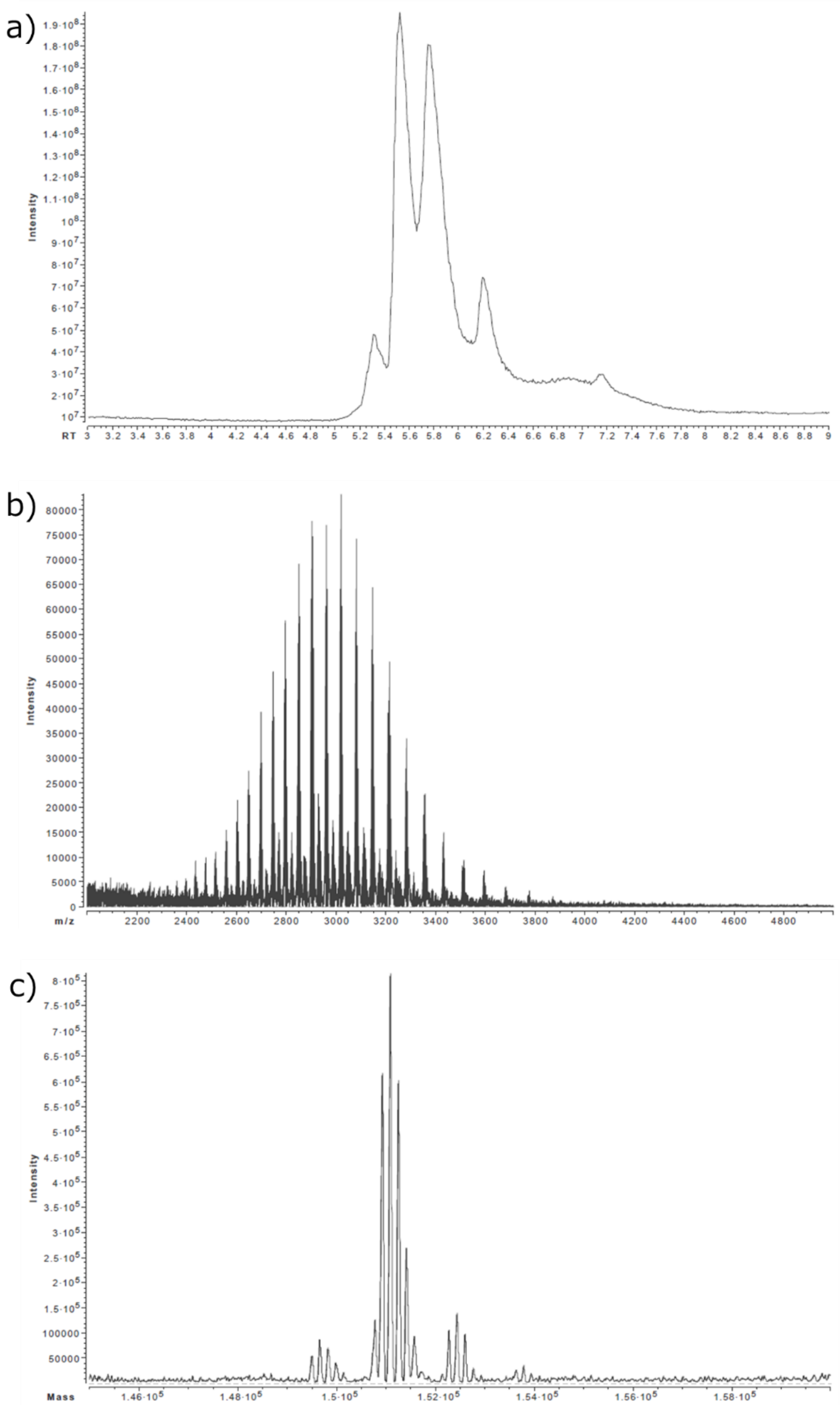

Figure S54. Provide a representative data of the n3 analysis of ADC 7 Day0, a) TIC within a retention time range of 3 to 9 minutes, b) Spectrum of *m/z* 2000 to 5000, c) Deconvoluted spectrum ranging from 1.45e5 to 1.60e5.

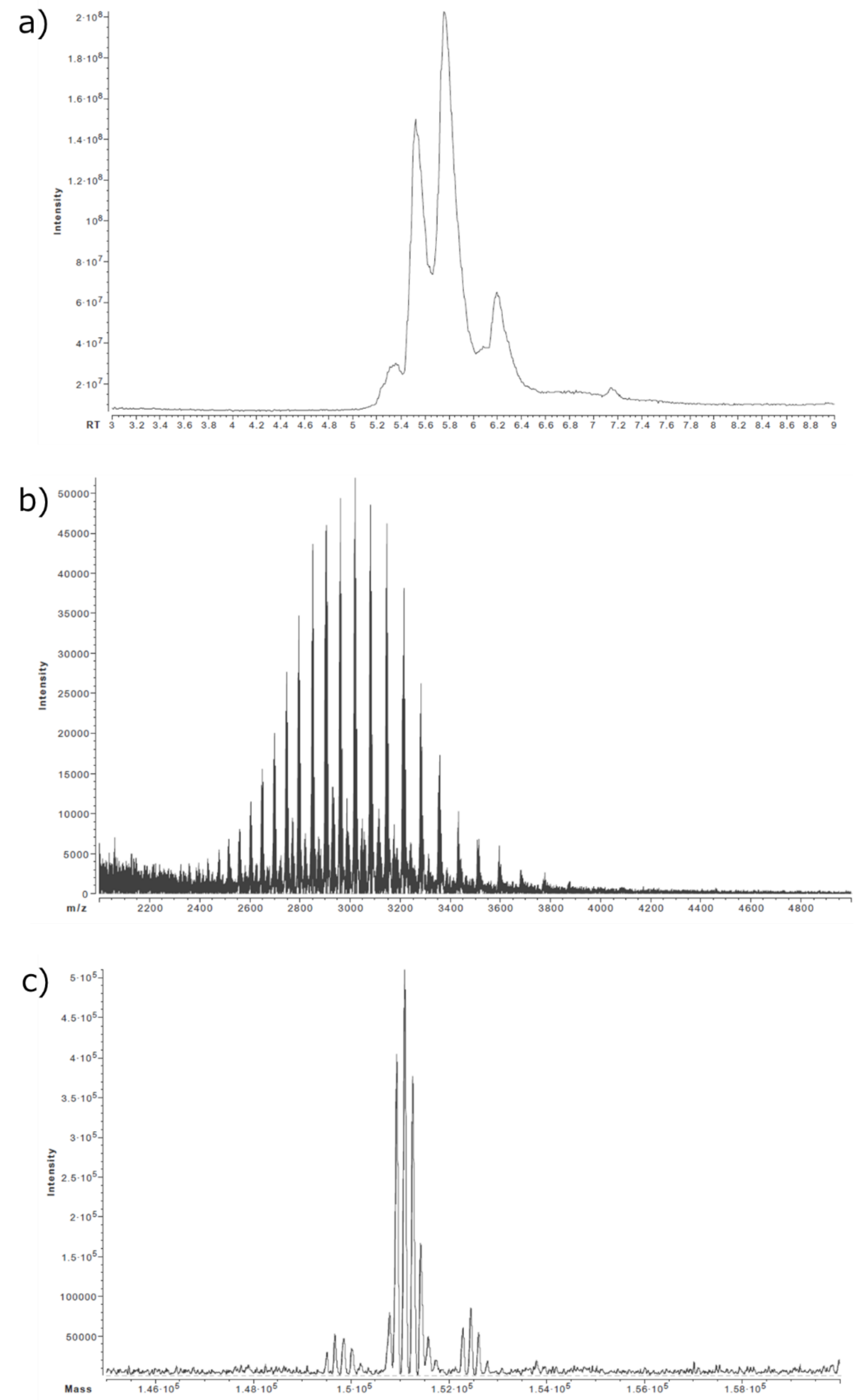

Figure S55. Provide a representative data of the n3 analysis of ADC 7 Day1, a) TIC within a retention time range of 3 to 9 minutes, b) Spectrum of *m/z* 2000 to 5000, c) Deconvoluted spectrum ranging from 1.45e5 to 1.60e5.

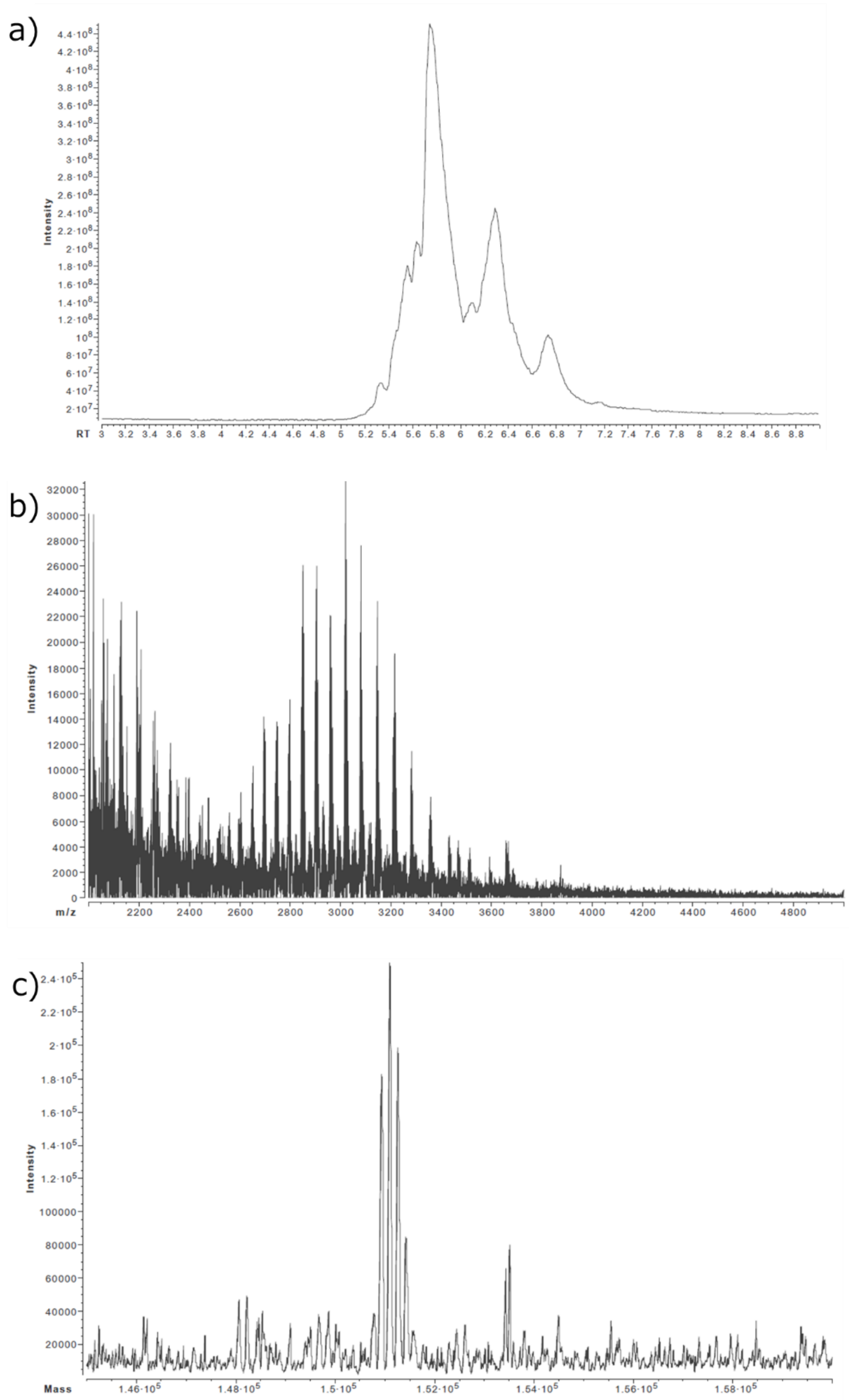

Figure S56. Provide a representative data of the n3 analysis of ADC 7 Day3, a) TIC within a retention time range of 3 to 9 minutes, b) Spectrum of *m/z* 2000 to 5000, c) Deconvoluted spectrum ranging from 1.45e5 to 1.60e5.

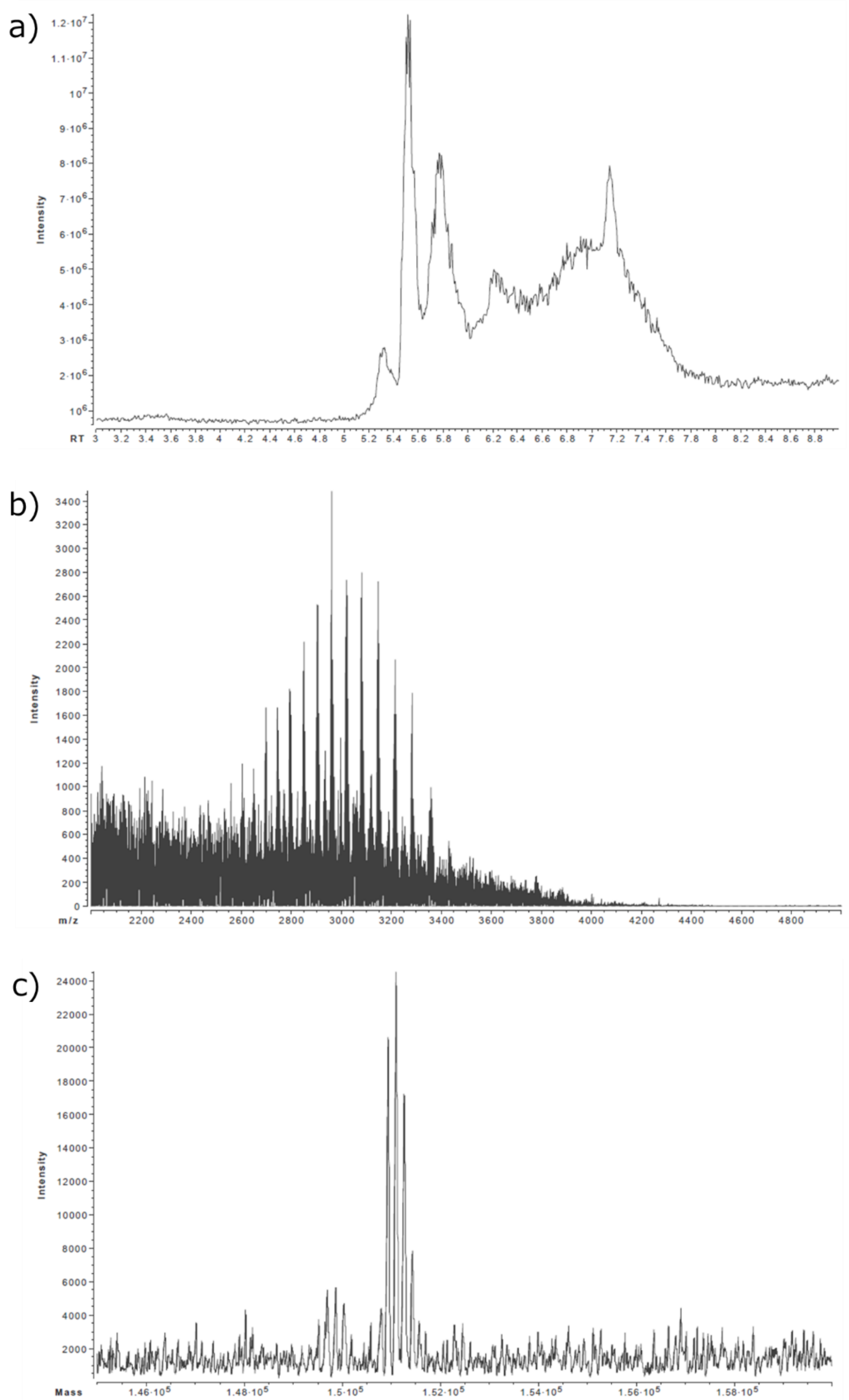

Figure S57. Provide a representative data of the n3 analysis of ADC 7 Day7, a) TIC within a retention time range of 3 to 9 minutes, b) Spectrum of *m/z* 2000 to 5000, c) Deconvoluted spectrum ranging from 1.45e5 to 1.60e5.

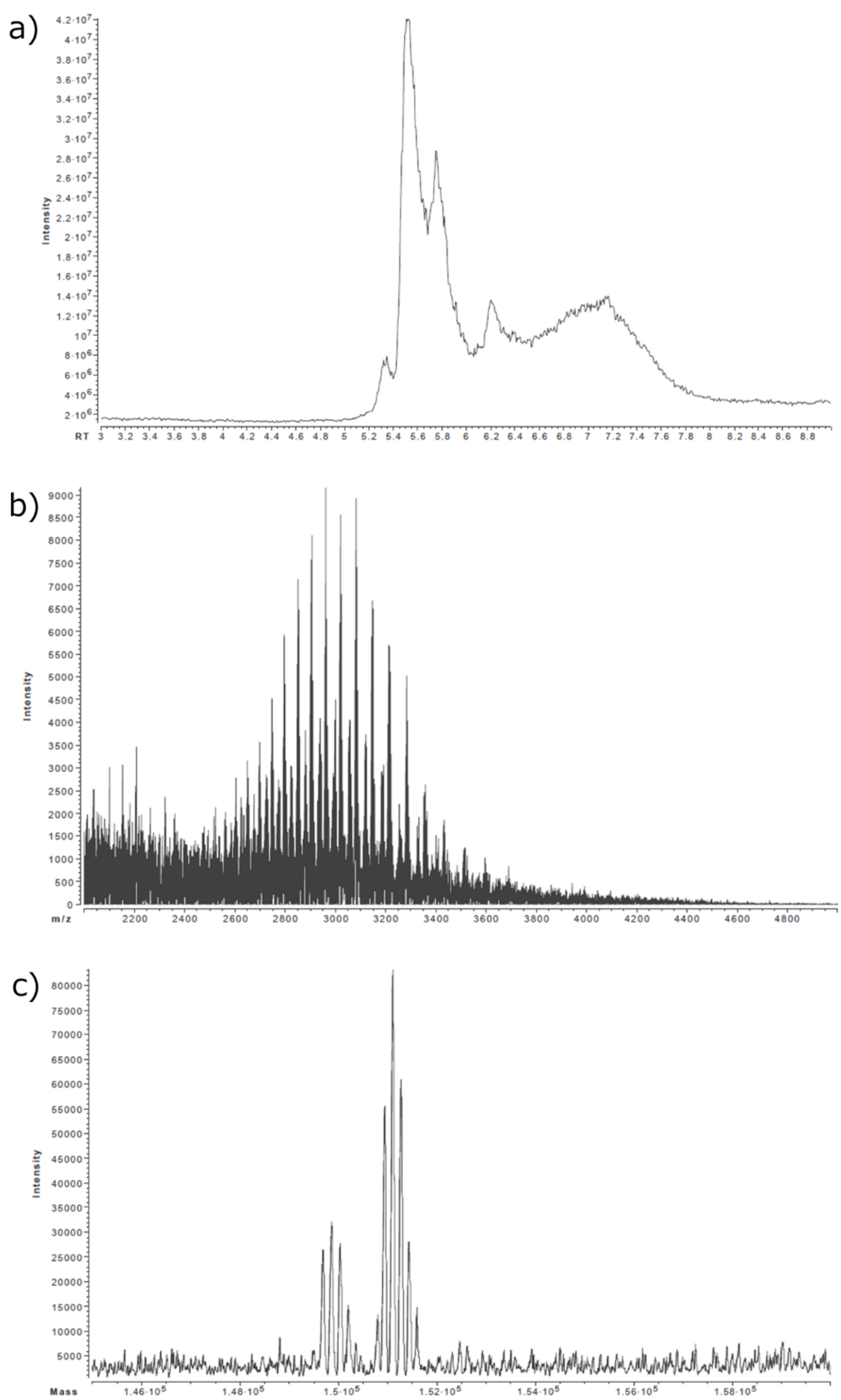

Figure S58. Provide a representative data of the n3 analysis of ADC 7 Day14, a) TIC within a retention time range of 3 to 9 minutes, b) Spectrum of *m/z* 2000 to 5000, c) Deconvoluted spectrum ranging from 1.45e5 to 1.60e5.

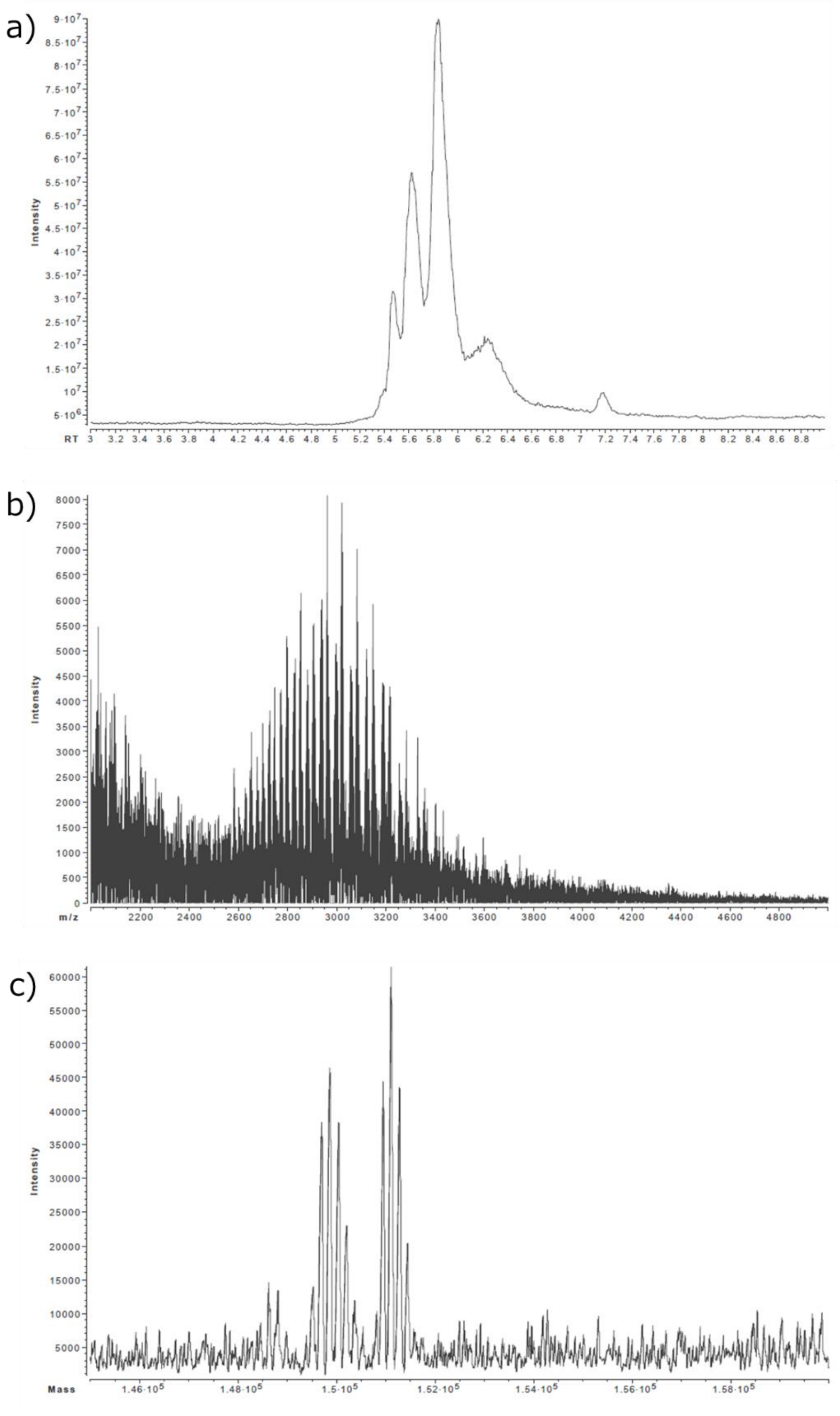

Figure S59. Provide a representative data of the n3 analysis of ADC 7 Day21, a) TIC within a retention time range of 3 to 9 minutes, b) Spectrum of *m/z* 2000 to 5000, c) Deconvoluted spectrum ranging from 1.45e5 to 1.60e5.

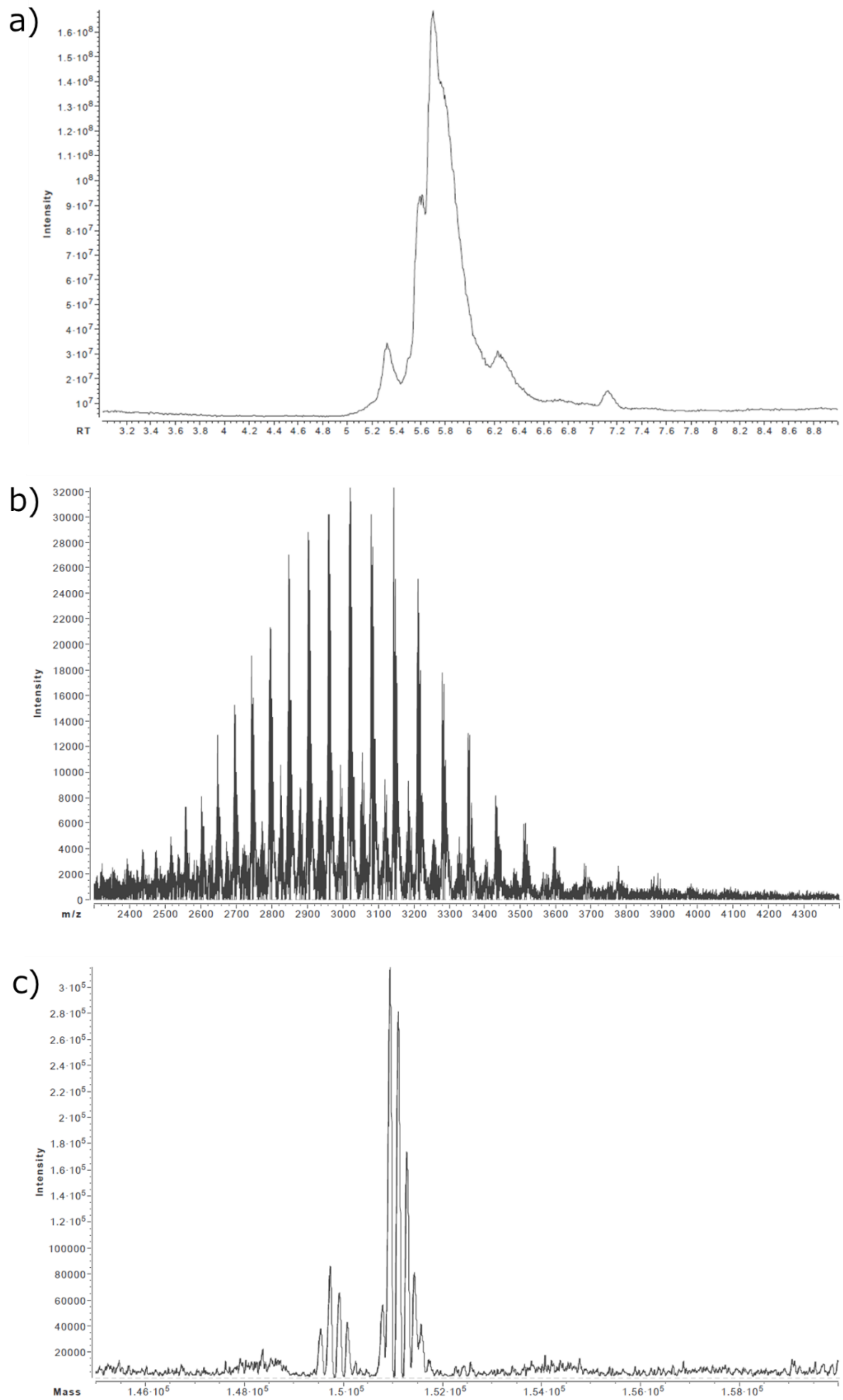

Figure S60. Provide a representative data of the n3 analysis of ADC 11 Day0, a) Total Ion Current (TIC) within a retention time range of 3 to 9 minutes, b) Spectrum of *m/z* 2300 to 4400, c) Deconvoluted spectrum ranging from 1.45e5 to 1.60e5.

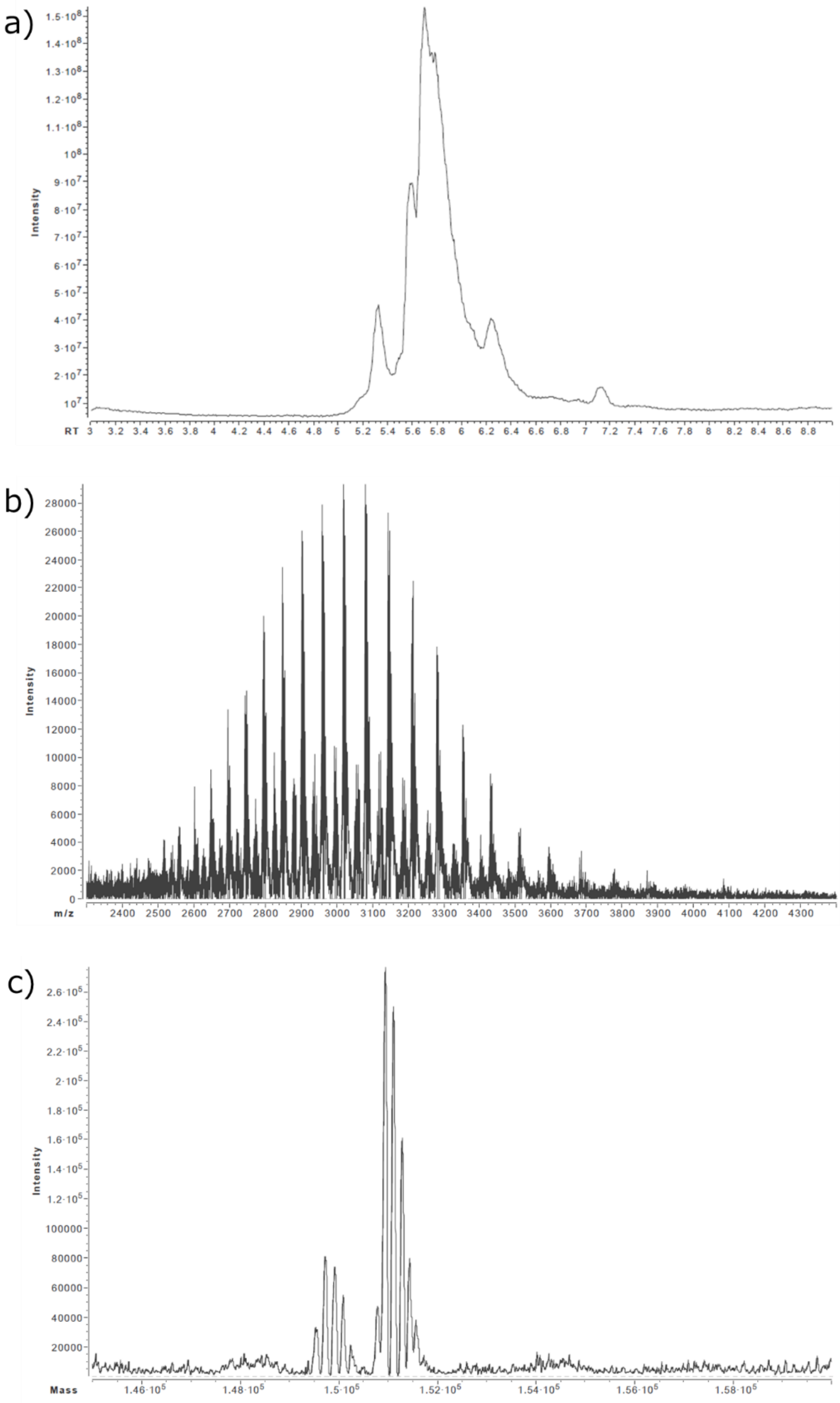

Figure S61. Provide a representative data of the n3 analysis of ADC 11 Day1, a) TIC within a retention time range of 3 to 9 minutes, b) Spectrum of *m/z* 2300 to 4400, c) Deconvoluted spectrum ranging from 1.45e5 to 1.60e5.

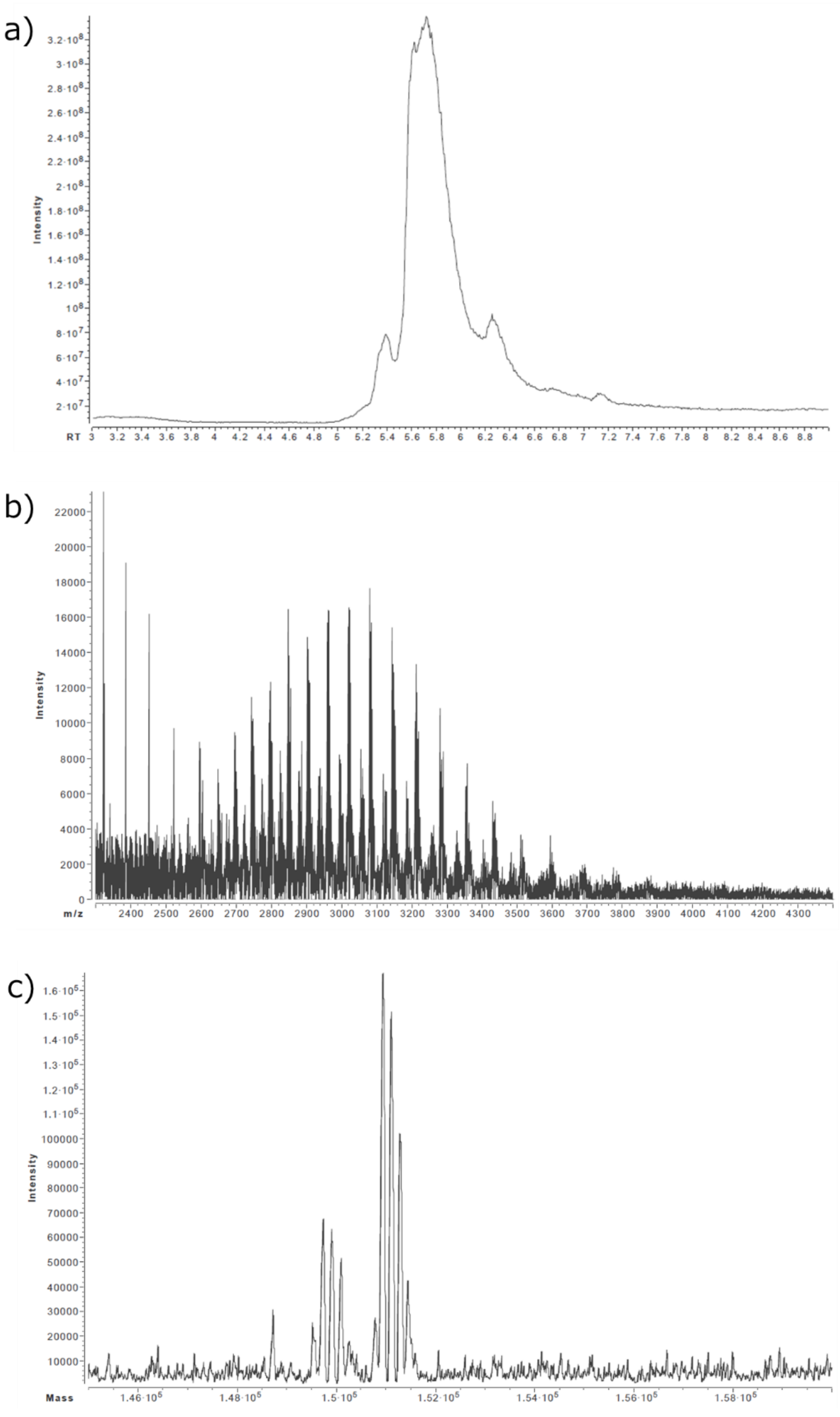

Figure S62. Provide a representative data of the n3 analysis of ADC 11 Day3, a) TIC within a retention time range of 3 to 9 minutes, b) Spectrum of *m/z* 2300 to 4400, c) Deconvoluted spectrum ranging from 1.45e5 to 1.60e5.

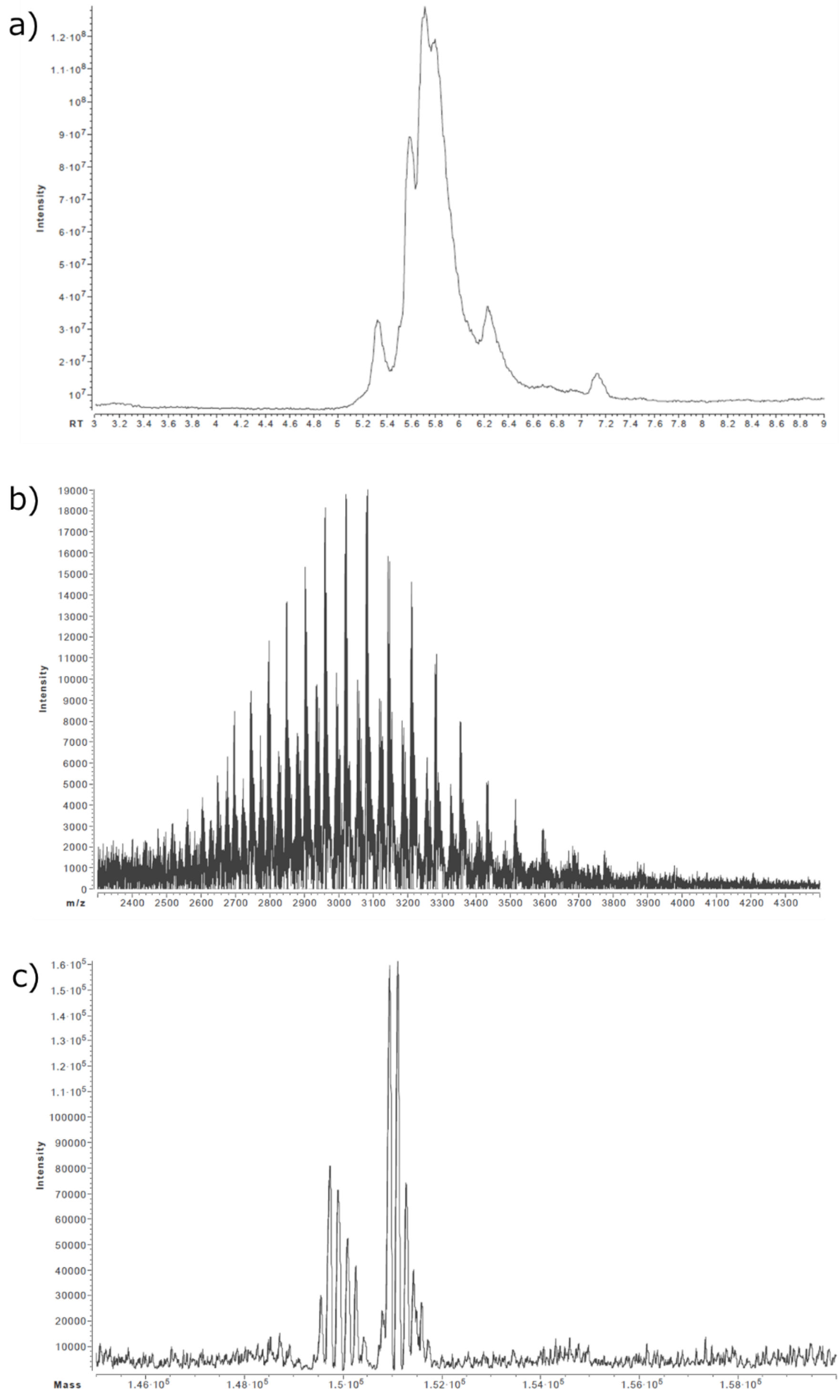

Figure S63. Provide a representative data of the n3 analysis of ADC 11 Day7, a) TIC within a retention time range of 3 to 9 minutes, b) Spectrum of *m/z* 2300 to 4400, c) Deconvoluted spectrum ranging from 1.45e5 to 1.60e5.

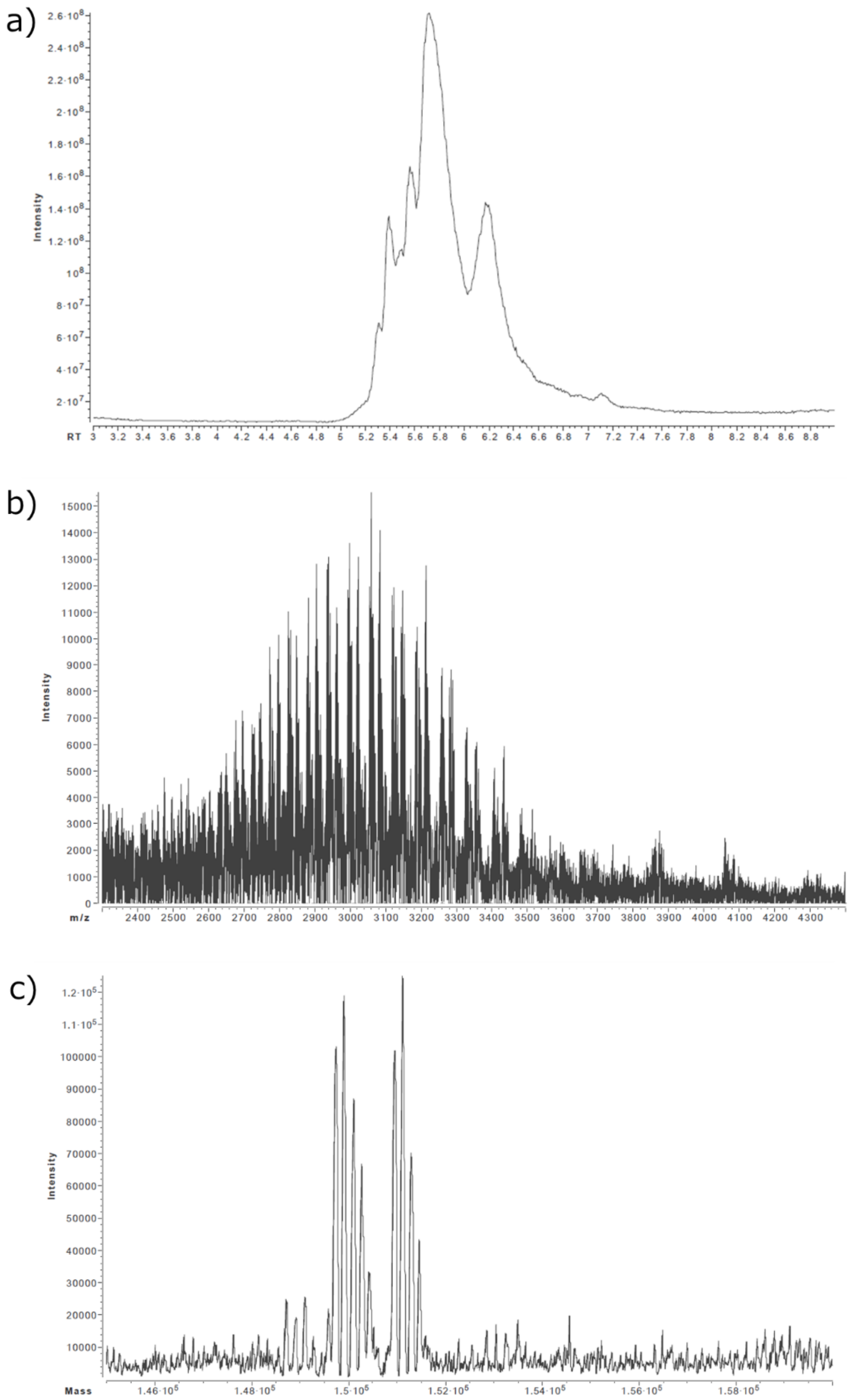

Figure S64. Provide a representative data of the n3 analysis of ADC 11 Day21, a) TIC within a retention time range of 3 to 9 minutes, b) Spectrum of *m/z* 2300 to 4400, c) Deconvoluted spectrum ranging from 1.45e5 to 1.60e5.

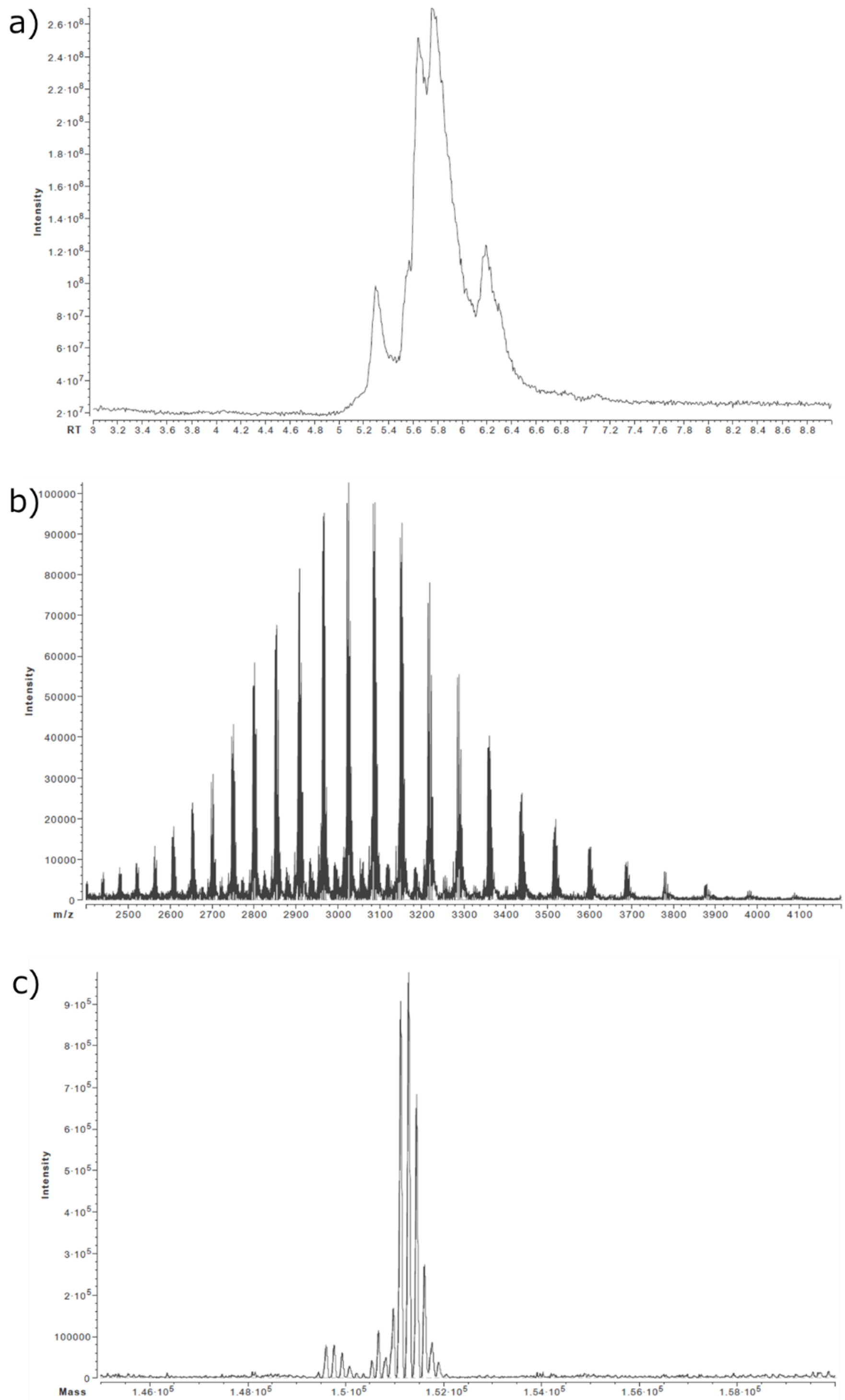

Figure S65. Provide a representative data of the n3 analysis of ADC 12 Day0, a) TIC within a retention time range of 3 to 9 minutes, b) Spectrum of *m/z* 2400 to 4200, c) Deconvoluted spectrum ranging from 1.45e5 to 1.60e5.

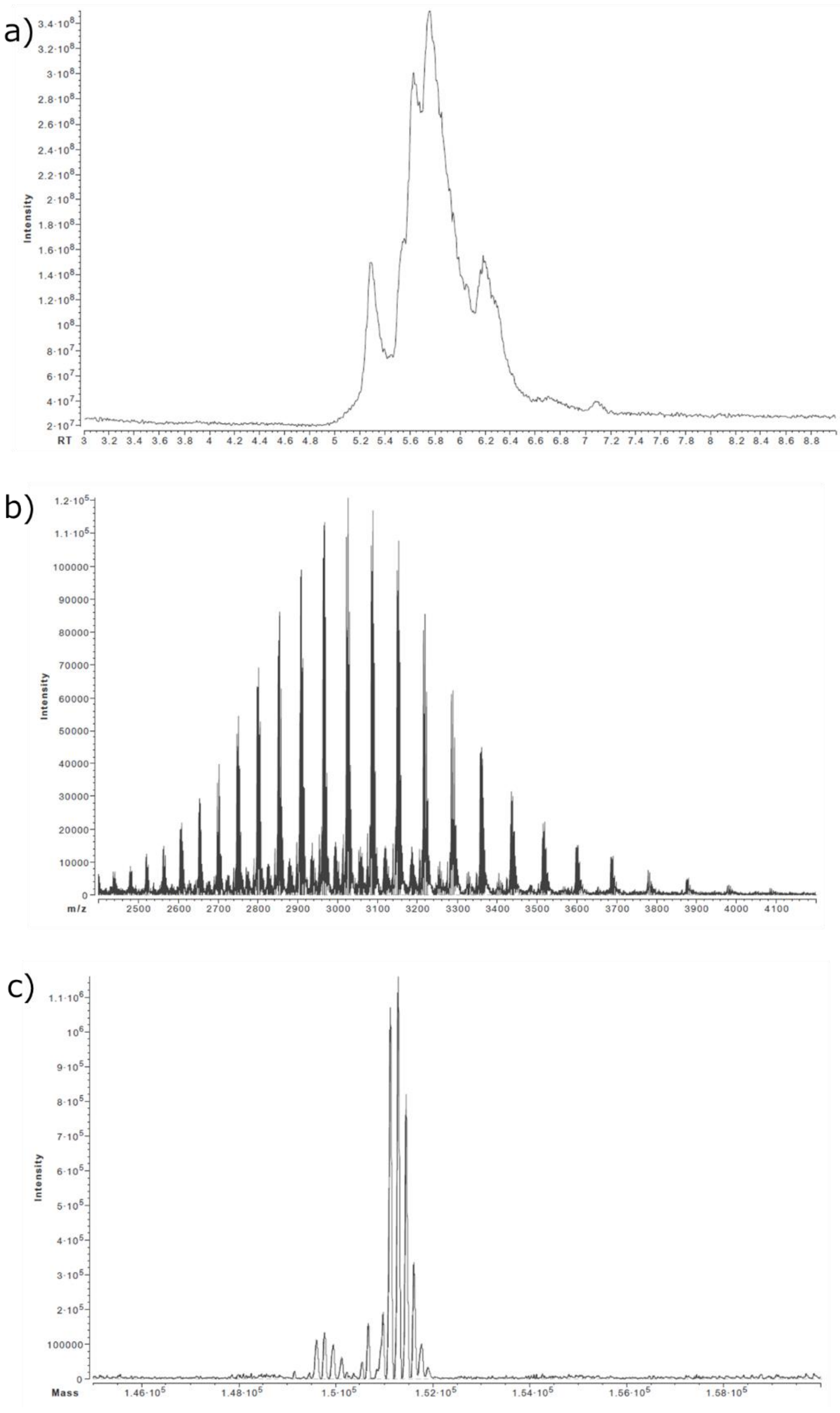

Figure S66. Provide a representative data of the n3 analysis of ADC 12 Day1, a) TIC within a retention time range of 3 to 9 minutes, b) Spectrum of *m/z* 2400 to 4200, c) Deconvoluted spectrum ranging from 1.45e5 to 1.60e5.

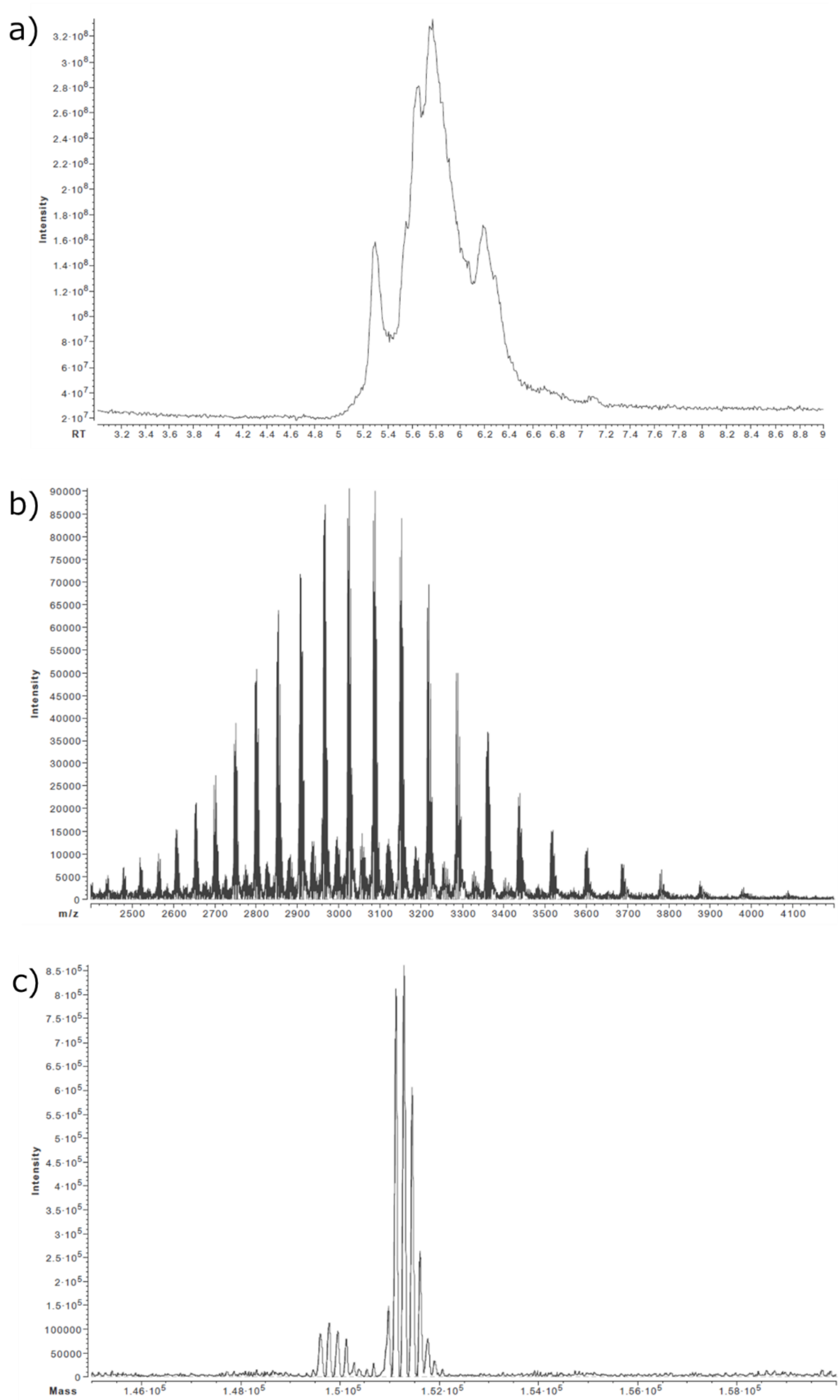

Figure S67. Provide a representative data of the n3 analysis of ADC 12 Day3, a) TIC within a retention time range of 3 to 9 minutes, b) Spectrum of *m/z* 2400 to 4200, c) Deconvoluted spectrum ranging from 1.45e5 to 1.60e5.

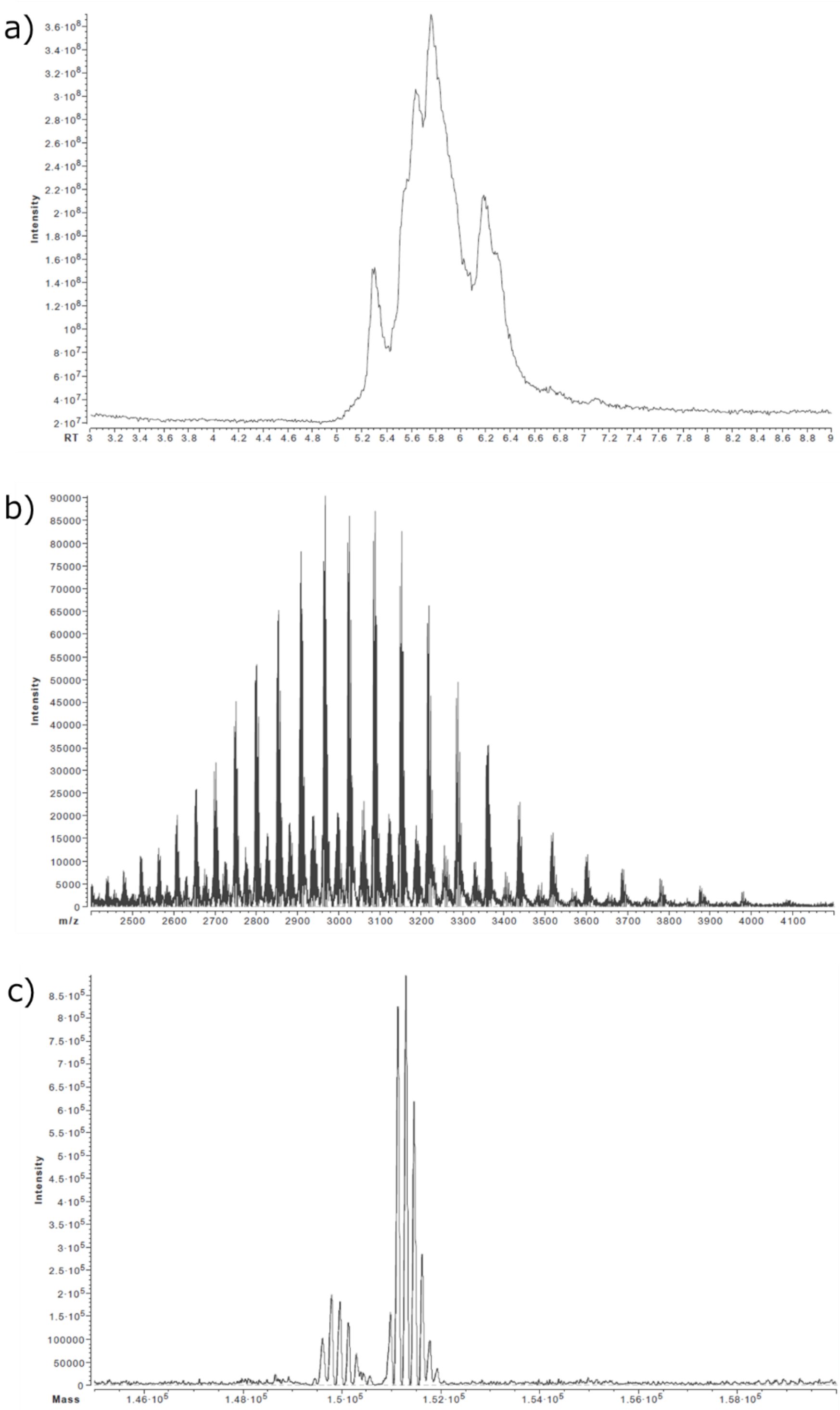

Figure S68. Provide a representative data of the n3 analysis of ADC 12 Day7, a) TIC within a retention time range of 3 to 9 minutes, b) Spectrum of *m/z* 2400 to 4200, c) Deconvoluted spectrum ranging from 1.45e5 to 1.60e5.

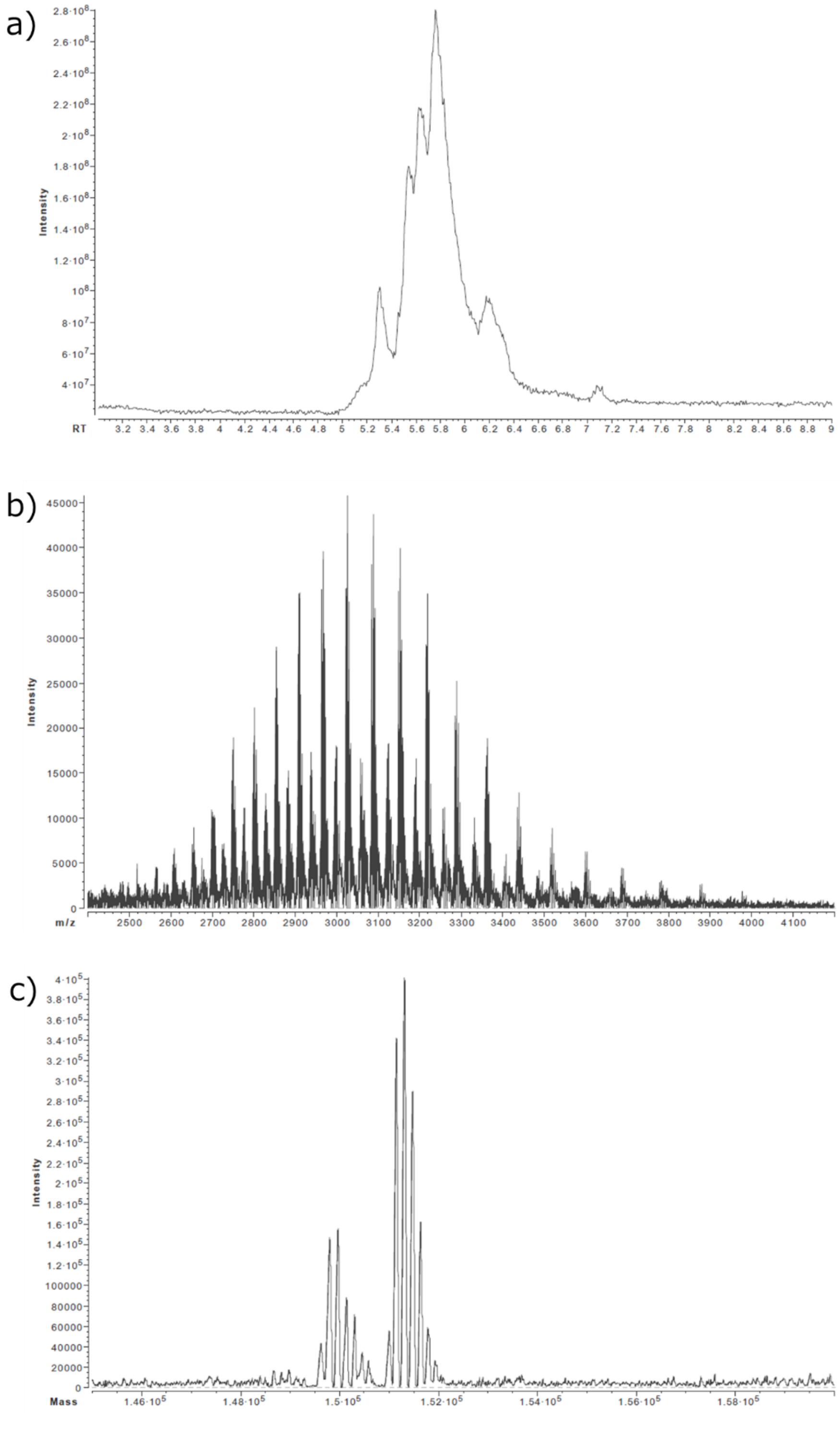

Figure S69. Provide a representative data of the n3 analysis of ADC 12 Day21, a) TIC within a retention time range of 3 to 9 minutes, b) Spectrum of *m/z* 2400 to 4200, c) Deconvoluted spectrum ranging from 1.45e5 to 1.60e5.

5 In vitro human neutrophil assay

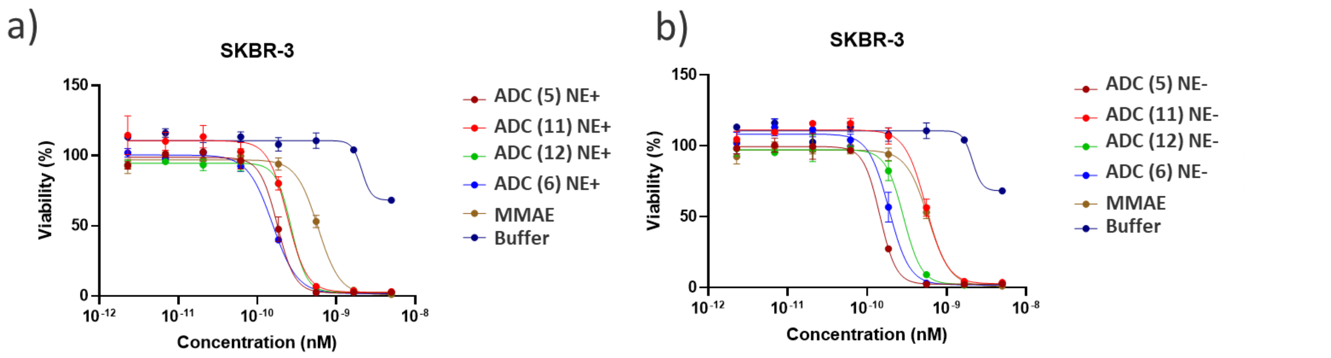

Figure S70 Evaluation of ADCs by *in vitro* cytotoxicity assay with HER2-high expressing SKBR-3 cells: a) NE-treated ADCs for 6 days. b) NE-untreated. The individual values and fitted curves are shown from the results of duplicated experiments.

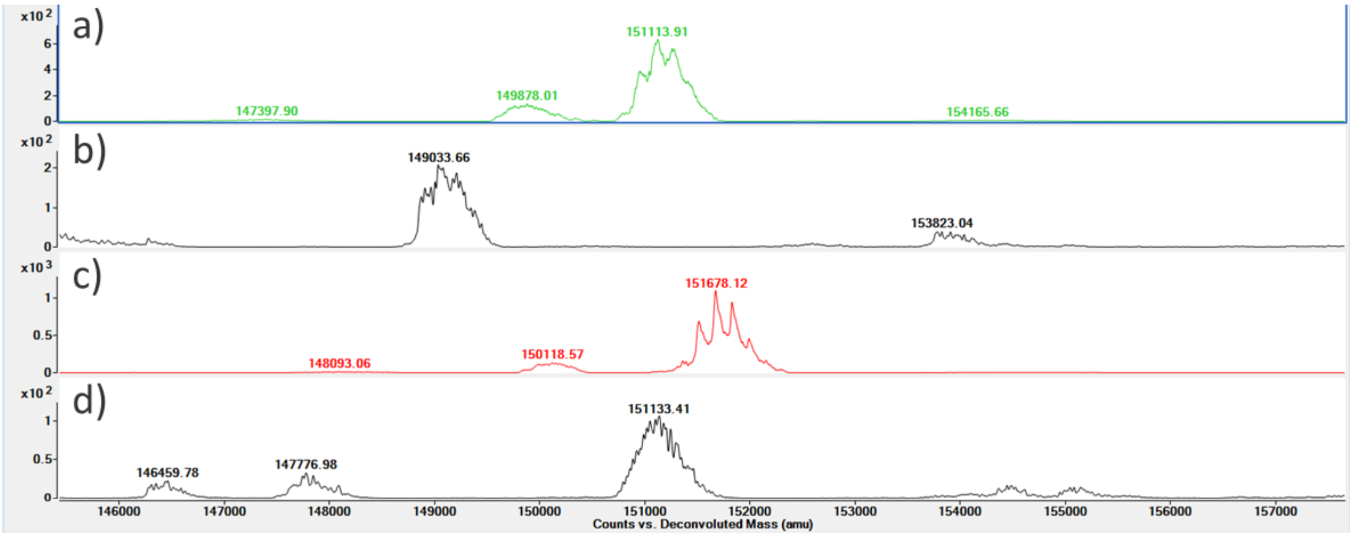

Figure S71 Deconvoluted spectra from Q-TOF MS analysis before and after NE treatment: (a) ADC 5 before treatment, (b) ADC 5 after treatment, (c) ADC 11 before treatment, (d) ADC 11 after treatment.

6 Comparison summary of linear and Exo-EVC Linkers

Table S1. Comparison of linear/Exo-EVC linker.

Antibody Conjugates	Linear VC	Linear EVC	Exo-EVC
Hydrophobicity	✗	✓	✓✓
High DAR applicability	✗	✗	✓✓
In vivo efficacy	✓	✓✓	✓✓
Rat PK	✓✓	✓✓	✓✓
CES1C resistance	✗	✓	✓
NE resistance	✗	✗	✓

[1] P. L. Salomon, E. E. Reid, K. E. Archer, L. Harris, E. K. Maloney, A. J. Wilhelm, M. L. Miller, R. V. J. Chari, T. A. Keating, R. Singh, *Mol Pharm* **2019**, *16*, 4817-4825.

[2] T. Fujii, Y. Matsuda, T. Seki, N. Shikida, Y. Iwai, Y. Ooba, K. Takahashi, M. Isokawa, S. Kawaguchi, N. Hatada, T. Watanabe, R. Takasugi, A. Nakayama, K. Shimbo, B. A. Mendelsohn, T. Okuzumi, K. Yamada, *Bioconjug Chem* **2023**, *34*, 728-738.

[3] Y. Matsuda, M. Leung, Z. Tawfiq, T. Fujii, B. A. Mendelsohn, *Anal Sci* **2021**, *37*, 1171-1176.

[4] Q. Zhu, A. Girish, S. Chattopadhaya, S. Q. Yao, *Chem Commun (Camb)* **2004**, 1512-1513.

[5] Y. Anami, C. M. Yamazaki, W. Xiong, X. Gui, N. Zhang, Z. An, K. Tsuchikama, *Nat Commun* **2018**, *9*, 2512.

[6] S. Yamazaki, N. Shikida, K. Takahashi, Y. Matsuda, K. Inoue, K. Shimbo, Y. Mihara, *Bioorg Med Chem Lett* **2021**, *51*, 128360.

[7] Y. Nakahara, B. A. Mendelsohn, Y. Matsuda, *Organic Process Research & Development* **2022**, *26*, 2766-2770.

[8] Y. Matsuda, T. Seki, K. Yamada, Y. Ooba, K. Takahashi, T. Fujii, S. Kawaguchi, T. Narita, A. Nakayama, Y. Kitahara, B. A. Mendelsohn, T. Okuzumi, *Mol Pharm* **2021**, *18*, 4058-4066.

[9] H. Zhao, S. Gulesserian, M. C. Malinao, S. K. Ganesan, J. Song, M. S. Chang, M. M. Williams, Z. Zeng, M. Mattie, B. A. Mendelsohn, D. R. Stover, F. Donate, *Mol Cancer Ther* **2017**, *16*, 1866-1876.

[10] S. Y. Y. Ha, Y. Anami, C. M. Yamazaki, W. Xiong, C. M. Haase, S. D. Olson, J. Lee, N. T. Ueno, N. Zhang, Z. An, K. Tsuchikama, *Mol Cancer Ther* **2022**, *21*, 1449-1461.

[11] Y. Ogitani, T. Aida, K. Hagihara, J. Yamaguchi, C. Ishii, N. Harada, M. Soma, H. Okamoto, M. Oitate, S. Arakawa, T. Hirai, R. Atsumi, T. Nakada, I. Hayakawa, Y. Abe, T. Agatsuma, *Clin Cancer Res* **2016**, *22*, 5097-5108.
